# A mechanism for the strange metal phase in rare-earth intermetallic compounds

**DOI:** 10.1073/pnas.2116980119

**Published:** 2022-03-01

**Authors:** Jiangfan Wang, Yung-Yeh Chang, Chung-Hou Chung

**Affiliations:** ^a^Department of Electrophysics, National Yang Ming Chiao Tung University, Hsinchu 30010, Taiwan, Republic of China;; ^b^Physics Division, National Center for Theoretical Sciences, Taipei 10617, Taiwan, Republic of China

**Keywords:** strange metal, quantum criticality, heavy fermions

## Abstract

A major mystery in strongly interacting quantum systems is the microscopic origin of the “strange metal” phenomenology, with unconventional metallic behavior that defies Landau’s Fermi liquid framework for ordinary metals. This state is found across a wide range of quantum materials, notably in rare-earth intermetallic compounds at finite temperatures (*T*) near a magnetic quantum phase transition, and shows a quasilinear-in-temperature resistivity and a logarithmic-in-temperature specific heat coefficient. Recently, an even more enigmatic behavior pointing toward a stable strange metal ground state was observed in CePd${}_{1-x}$Ni*_x_*Al, a geometrically frustrated Kondo lattice compound. Here, we propose a mechanism for such phenomena driven by the interplay of the gapless fermionic short-ranged antiferromagnetic spin correlations (spinons) and critical bosonic charge (holons) fluctuations near a Kondo breakdown quantum phase transition. Within a dynamical large-*N* approach to the Kondo–Heisenberg lattice model, the strange metal phase is realized in transport and thermodynamical quantities. It is manifested as a fluctuating Kondo-scattering–stabilized critical (gapless) fermionic spin-liquid metal. It shows ω/T scaling in dynamical electron scattering rate, a signature of quantum criticality. Our results offer a qualitative understanding of the CePd${}_{1-x}$Ni*_x_*Al compound and suggest a possibility of realizing the quantum critical strange metal phase in correlated electron systems in general.

## Significance

The elusive strange metal phase (ground state) was observed in a variety of quantum materials, notably in *f*-electron–based rare-earth intermetallic compounds. Its emergence has remained unclear. Here, we propose a generic mechanism for this phenomenon driven by the interplay of the gapless fermionic short-ranged antiferromagnetic spin correlation and critical bosonic charge fluctuations near a Kondo breakdown quantum phase transition. It is manifested as a fluctuating Kondo-scattering–stabilized critical (gapless) fermionic spin liquid. It shows ω/T scaling in dynamical electron scattering rate, a signature of quantum criticality. Our results on quasilinear-in-temperature scattering rate and logarithmic-in-temperature divergence in specific heat coefficient as temperature vanishes were recently seen in CePd${}_{1-x}$Ni*_x_*Al.

A major mystery in strongly interacting quantum systems is the microscopic origin of “strange metal” behavior, an unstable finite-temperature quantum state with unconventional metallic behavior that defies Landau’s Fermi liquid (FL) framework for ordinary metals. This state is found across a wide range of quantum materials, ranging from cuprate superconductors (1–3), to rare-earth intermetallic compounds (4, 5), to quantum dot (6), to magic-angled twisted bilayer graphene (7). This non-Fermi liquid (NFL) behavior often exists near a magnetic quantum critical point (QCP) (4, 8) and shows a quasilinear-in-temperature resistivity and a logarithmic-in-temperature specific heat coefficient. In particular, a number of rare-earth intermetallic compounds displaying strange metal behavior, including YbRh_2_Si_2_ (9, 10), CeRhIn_5_ (11), and CePdAl (12), also show Kondo breakdown (KB) transition, a mechanism where the conventional quasiparticle description completely breaks down when the Kondo effect is suppressed (13–15). These findings go beyond the standard Hertz–Millis spin-density-wave (SDW) theory (16, 17). It is commonly accepted that the Doniach framework (18), i.e., the competition of Kondo correlations with magnetic long-range interactions, is at the heart of the problem. Previous attempts have been made via different approaches to capture various aspects of the problem (13, 19–24). However, a microscopic understanding of the strange metal behavior is still incomplete.

Recently, an even more intriguing paramagnetic NFL behavior pointing toward a stable strange metal “phase” at ground state was observed in the geometrically frustrated heavy-electron Kondo lattice systems. Examples of such behavior include CePd${}_{1-x}$Ni*_x_*Al (0≤x≤1) near the KB QCP on a kagomé lattice (12, 25–28) and YbAgGe, a triangular lattice system (29). In these materials, the geometrical frustration suppresses the antiferromagnetically (AF) long-range order (LRO) and likely leads to a magnetically short-range–ordered spin-liquid state with fractional spin excitations (or spinons). A similar strange metal phase without long-range order has been observed in unfrustrated Kondo lattice Ge-substituted YbRh_2_Si_2_, where experimental evidence (30, 31) indicates the breakup of the heavy electrons into separate spin and charge parts. The microscopic origin of this emergent quantum phase of matter has remained enigmatic and searching for the mechanism for this exotic phase is essential to establish a general theoretical framework of it. This strange metal phase goes beyond the well-known Hertz–Millis SDW theory (16, 17). We thus expect that a distinct mechanism for the competition of the spin short-range order strange metal phase and the Kondo-screened FL phase is needed.

Motivated by these puzzling experimental observations, we are led to the following fundamental theoretical questions: How can a stable strange metal phase exist in principle given that the strange metal properties mostly appear near an unstable QCP? Can this phase emerge from the competition or collaboration between Kondo-screening and spin-liquid states? Would physical quasiparticles effectively get fractionalized into spin (spinon) and charge (Kondo) excitations and the strange metal phase be closely connected to critical fluctuations of either excitation? Is there ω/T scaling in dynamical observables, a signature of quantum criticality?

In this paper, we develop a controlled method to address these issues based on a dynamical large-*N* approach combined with the idea of heavy-electron fractionalization to the Kondo–Heisenberg (KH) model on a two-dimensional (2D) lattice (22, 23, 32). We discover a NFL strange metal phase near the KB QCP, which separates the Kondo-screened heavy FL and the strange metal spin-liquid states. Therein, the static electron scattering rate at Fermi energy shows a quasilinear-in-*T* behavior, while the dynamical electron scattering rate exhibits an ω/T scaling, a typical signature of quantum criticality. We attribute these features to the interplay of critical bosonic charge (Kondo) fluctuations and gapless fermionic spinons. We clarify the nature of this phase in terms of the fluctuating Kondo-scattering–stabilized critical spin-liquid metal. The specific heat coefficient and spin susceptibility in this phase exhibit NFL logarithmic-in-temperature divergence as T→0. Our results provide a qualitative understanding of the strange metal phase observed in CePd${}_{1-x}$Ni*_x_*Al and suggest a possibility of realizing the quantum critical strange metal phase in correlated electron systems in general.

## Results

### Dynamical Large-*N* Multichannel Approach.

We first develop a controlled large-*N* approach to address the issue on the strange metal phase. Our starting point is the KH model on a 2D square lattice,

[1] $$H=H_{0}+H_{K}+H_{J}.$$

Here, the conduction electron reservoir is described by the sum of independent electron baths with constant density of states, $H_{0}=\sum_{iP\alpha }\varepsilon_{P}\psi_{i\alpha }^{†}(P)\psi_{i\alpha }(P)$ with α=±1 being the SU(2) spin index. Here, $\psi_{i\alpha }=\sum_{P}\psi_{i\alpha }(P)$ destroys a conduction electron on the lattice at site *i* with $\psi_{i\alpha }(P)$ being its Fourier component with momentum ***P*** defined on the “bath lattice” and $\varepsilon_{P}$ being its dispersion. For simplicity, we take the conduction bath to be at half filled, showing particle–hole symmetry. Each local impurity spin at site *i*, $S_{i}$, can be screened by the conduction-electron spin $s_{i}^{c}=(1/2)\sum_{\alpha \beta }\psi_{i\alpha }^{†}\sigma_{\alpha \beta }\psi_{i\beta }$ of the same site via the Kondo coupling: $H_{K}=J_{K}\sum_{i}S_{i}\cdot s_{i}^{c}$. The nearest-neighbor local spins are coupled by an antiferromagnetic Heisenberg term $H_{J}=\sum_{\langle i,j\rangle }J_{H}S_{i}\cdot S_{j}$. The local spin operator is represented by the Abrikosov pseudofermion $S_{i}=\frac{1}{2}\sum_{\alpha \beta }f_{i\alpha }^{†}\sigma_{\alpha \beta }f_{i\beta }$, subject to the local constraint, $n_{f}(i)=\sum_{\alpha }f_{i\alpha }^{†}f_{i\alpha }=Q$. We then generalize the spin-1/2 KH model on a square lattice to the multichannel large-*N* limit by considering *K* independent Kondo-screening channels with channel-asymmetric couplings and *N* flavors of spin species with N,K→∞, while we set *Q* = *K* and fix κ≡K/N. The large-*N* channel-asymmetric multichannel generalization of the Kondo and Heisenberg terms from the single-channel physical SU(2) limit reads (*Materials and Methods*)

[2] $$\begin{array}{l} H_{K}=\left(-\frac{1}{N}\right)\sum_{i,a,\alpha ,\beta }J_{K}^{a}\psi_{ia\alpha }^{†}f_{i\alpha }f_{i\beta }^{†}\psi_{ia\beta },\\ H_{J}=\left(-\frac{J_{H}}{N}\right)\sum_{\langle i,j\rangle ,\alpha \beta }\left(\tilde{\alpha }f_{i\alpha }^{†}f_{j,-\alpha }^{†}\right)\left(\tilde{\beta }f_{j,-\beta }f_{i\beta }\right), \end{array}$$

where α,β=±1,⋯,±N/2 represent the spin flavors, a=1,⋯,K denotes the channel index, $J_{K}^{a}$ is a channel-dependent Kondo coupling, and $\tilde{\alpha }\equiv \text{sgn}(\alpha )$. Here, we employ the fermionic Sp(*N*) generalization of the SU(2) Heisenberg *H_J_* term (33). The Kondo term *H_K_* shows SU(*K*) channel symmetry when $J_{K}^{a=1}=\cdots =J_{K}^{a=K}$; however, in this work, we consider the channel asymmetric limit where $J_{K}^{a=K}\equiv J_{K}\prime >J_{K}^{a<K}\equiv J_{K}$ so that the SU(*K*) channel symmetry is broken down to SU(*K* – 1). Note that for symmetric (bosonic) representations of spins in the large-*N* Kondo models, it is essential to add the extra channels to obtain a Kondo energy extensive in *N* (34). However, for the antisymmetric (fermionic) representation of the spins in such models, one does not need to add additional channels to obtain a good large-*N* expansion (35). Our fermionic large-*N* approach departs from the conventional single-channel large-*N* approach by adding extra Kondo-screening channels for the following advantages: It includes NFL effects in the large-*N* limit, allowing an exploration of possible strange metal physics. By doing so, one can maintain the physical value of κ=1/2 important for a conventional Kondo lattice, while also bringing in non-Fermi liquid physics in the large-*N* limit. In particular, the channel-asymmetric multichannel Kondo lattice model we develop here further advances the previously studied channel-symmetric one (19). It opens up a possibility to have fully Kondo-screened FL and overscreened Kondo NFL phases as well as the possible emergent strange metal phases appearing as the ground state of the generic phase diagram (below). By contrast, the channel-symmetric multichannel Kondo model supports only the overscreened Kondo NFL state, but not the FL state (19). Our approach therefore constitutes a distinct approach to the large-*N* expansion.

**Fig. 1.**
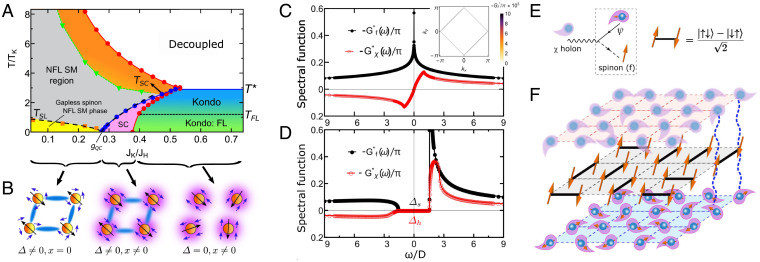
The finite-temperature phase diagram, spectral weight of the spinon and holon fields, and schematic illustration of the composite holon, spin-singlet bond, and the gapless strange metal (SM) phase. (*A*) The finite-temperature phase diagram of the large-*N* channel-asymmetric Kondo lattice model with κ=1/2 and $J_{K}\prime =2J_{K}$. The cross-over scales on both sides of *g_QC_* exhibit power-law behavior in $\left|g-g_{QC}\right|$ (black dashed lines). Here, $T_{K}=D\exp\;(-2D/J_{K})\approx 0.1$ with fixed half-bandwidth $D=1=J_{K}$. (*B*) Schematic plot of different mean-field phases: The black and blue arrows represent the local and conduction-electron spins, respectively. The spin singlet (blue) bonds connect two adjacent local spins. In the Kondo FL phase, RVB singlets are suppressed, and the coherent Kondo screening (purple) cloud is formed at each site. In the RVB-dominated strange metal phase, spinons in the RVB singlets couple to the Kondo fluctuations. In the coexisting superconducting phase, both RVB singlets and coherent Kondo screening are present. (*C*) The spinon and holon spectral function for κ=1/2 displays a power-law behavior at a low-frequency limit. *Inset* shows spinon Fermi surface, $-G_{f}^{\prime \prime }(\omega =0,k)/\pi$, for κ=1/2 at low temperature, $T/T_{K}=0.05$. (*D*) The spectral weight of the holon and spinon fields for κ=0.3 shows a gap at low frequency, denoted as Δ*_h_* and Δ*_s_*. We fix *g* = 0.052 and $T/T_{K}=0.05$ for *C* and *D*. (*E*) *Left*, schematic representation for generating a composite holon (*χ*): *χ*, represented by a blurred blue sphere surrounded by a blurred purple cloud, is generated by creating a spinon (*f*, orange arrow) and annihilating a conduction electron (*ψ*, a solid blue sphere with an orange arrow surrounded by a purple cloud) through the Kondo interaction vertex connecting the wavy, solid, and dashed lines. *Right*, schematic representation of a RVB spin-singlet bond. (*F*) Schematic plot of the gapless strange metal spin-liquid phase in *A*.

To make progress, we work on a simple square lattice where the magnetic frustration via the disorder effect (e.g., random exchange couplings) (36) may lead to a spin-liquid state (24). We expect that our results qualitatively apply for geometrically frustrated lattices to which our large-*N* approach is readily generalized. This expectation is borne out as suggested by the comparison to experiments. This approach is appropriate to study paramagnetic spin-liquid to Kondo quantum phase transition. The AF Heisenberg term with a coupling *J_H_* and Sp(*N*) symmetry (20) is described by Anderson’s AF short-ranged resonating-valence-bond (RVB) spin liquid (37) made of the uniform spin singlets $\Delta_{ij}=\Delta =J_{H}\sum_{\alpha }\langle \text{sgn}(\alpha )f_{i\alpha }^{†}f_{j,-\alpha }^{†}\rangle$. The Kondo hybridization with coupling constants $J_{K},\;J_{K}\prime$ is expressed by the spin-charge–separated fermionic spinons and bosonic (charged) holon (Fig. 1*E*). In particular, holons are composite boson fields *χ_ia_*, consisting of Bose-condensed part $x=(J_{K}\prime /\sqrt{N})\sum_{i\alpha }\langle \psi_{i\alpha ,a=K}^{†}f_{i\alpha }\rangle$ on the *K*th channel and the fluctuating fields ${\hat{\chi }}_{ia}$ on the remaining *K* – 1 channels; i.e., $\chi_{ia}\to x\sqrt{N}\delta_{a,K}+{\hat{\chi }}_{ia}(1-\delta_{a,K})$. We apply the independent conduction bath approximation (38) (*SI Appendix*, sections S.I. and S.II) and set $J_{K}\prime =2J_{K}$. When the Kondo effect dominates, the above channel-asymmetric Kondo couplings lead to a condensation of the bosonic Kondo hybridization field (x≠0) and therefore favor a Kondo-screened Fermi-liquid phase. We solve the self-consistent Dyson-type equations for the dynamical (frequency-dependent) Green’s functions of various fields (*Materials and Methods* and *SI Appendix*, section S.II) (34, 36). This approach allows us to simultaneously explore the Kondo-screened and fermionic spin-liquid phases as well as possible static and dynamical strange metal properties via the interplay of these two, which goes beyond the static large-*N* mean-field theories and offers additional insight among existing approaches (20, 36, 39–41). This additional insight includes the possible NFL strange metal phase, as well as dynamical correlations and quantum critical ω/T scaling of physical observables therein. The fluctuating $\hat{\chi }$ field plays a crucial role in the development of the strange metal phase. To stabilize the strange metal phase, we find it is essential to preserve the particle–hole symmetry of the pseudofermion (*f* field); but this symmetry is not required for the conduction-electron bath (see below and *SI Appendix*, sections S.IV and S.IX).

### Finite-Temperature Phase Diagram.

Fig. 1*A* shows the finite-temperature phase diagram of this model for κ=1/2, in which the half-filled *f* electrons show particle–hole symmetry (*SI Appendix*, section S.IV), as a function of $g=J_{K}/J_{H}$ and dimensionless temperature $T/T_{K}$ with *T_K_* being the single-impurity Kondo temperature. The RVB spin-liquid metal phase dominates for small values of *g* (Δ≠0, *x* = 0; yellow, gray, and orange in Fig. 1*A*), while the Kondo-screened paramagnetic heavy-electron phase prevails at large *g* (Δ  =  0, x≠0; green and blue in Fig. 1*A*). A coexisting phase, an extended *s*-wave superconducting phase when electron baths are connected, is found at intermediate *g* (Δ≠0, x≠0; pink in Fig. 1*A*) (40, 42). A high-temperature decoupled phase is reached when Δ=x=0.

The $T^{*}$ (blue in Fig. 1*A*) line sets the boundary between *x* = 0 and x≠0. In the pure Kondo regime, $T^{*}$ corresponds to the mean-field Kondo coherence temperature below which the Bose-condensed Kondo hybridization develops phase coherence over the lattice, with a value $T^{*}>T_{K}$ (43). At lower temperatures $T<T_{FL}$, the system becomes a Fermi liquid where the specific heat coefficient reaches a constant at $T∼T_{FL}$ (*SI Appendix*, section S.IX).

In Fig. 1*A*, a quantum critical region (gray area) emerges due to the QCP at $g=g_{QC}$, characterized by a non-Fermi liquid behavior (below). The signature of quantum-critical behavior near *g_QC_* is supported by the power-law-in-$\left|g-g_{QC}\right|$ cross-overs on both sides of *g_QC_* (black-dashed lines in Fig. 1*A*): $T_{SL}∼|g-g_{QC}{|}^{m}$ and $T_{SC}∼{\left(g-g_{QC}\right)}^{n}$ with m≈0.6 and n≈0.8. For $g<g_{QC}$ and at temperatures below *T_SL_* (Fig. 1*A*), the thermodynamical observables and transport show an exotic NFL strange metal phase as T→0 (below).

### The Fluctuating Kondo-Scattering–Stabilized Gapless Spin-Liquid Strange Metal Phase.

Interestingly, due to particle–hole symmetry of our model, the spectral functions of the spinon [$-G_{f}^{\prime \prime }(\omega )/\pi$: imaginary part of the spinon Green’s function $-G_{f}(\omega )$] and that of the holon [$-G_{\chi }^{\prime \prime }(\omega )/\pi$] in the strange metal phase become gapless. In particular, we find a power-law singular (pseudogap vanishing) spinon (holon) spectral function near Fermi energy (Fig. 1*C*), respectively, indicating an exotic Kondo-scattering–stabilized gapless (critical) fermionic spin-liquid phase (27, 44). The spectral function of the *f* electron $\left(-1/\pi )G_{f}^{\prime \prime }(\omega =0,k\right)$ reveals a diamond-shaped spinon Fermi surface with gapless spinon excitations (Fig. 1 *C*, *Inset*). The effective Hamiltonian of the spin liquid in the strange metal phase can then be described as

[3] $${\tilde{H}}_{f}^{SM}=\sum_{k\alpha }\varepsilon_{\gamma }(k)\gamma_{k\alpha }^{†}\gamma_{k\alpha },$$

where the spinon dispersion $\varepsilon_{\gamma }(k)=\Delta (\cos\;k_{x}+\cos\;k_{y})$ (the lattice constant is set to be the unit of length) shows gapless lines along $|k_{x}\pm k_{y}|=\pi$ and Bogoliubov diagonalized spinon field $\gamma_{k\alpha }=(f_{k\alpha }+f_{-k,-\alpha }^{†})/\sqrt{2}$. The singular spinon spectral function at the low-frequency regime in Fig. 1*C* can be understood via the Van Hove singularity of the above gapless fermionic spinons at half filling.

This strange metal phase is a gapless RVB spin liquid, distinct from the previously studied spin liquids, such as the algebraic gapless spin liquid in the frustrated Heisenberg antiferromagnet (44, 45) and flux phase in the *t*-*J* model (46). In this phase, the quasiparticles break up into fermionic spinons and bosonic holons (Fig. 1*E*). In the absence of Kondo fluctuation, the system is in the fractionized Fermi liquid (FL${}^{⋆}$) phase, reminiscent of that described in ref. 20, consisting of gapped fermionic spinons and a decoupled conduction band. Note, however, that our FL${}^{⋆}$ phase exhibits a staggered local U(1) gauge symmetry: $f_{i\alpha }\to f_{i\alpha }e^{i\theta_{i}},\;f_{j\alpha }\to f_{j\alpha }e^{i\theta_{j}}$ with $\theta_{i}=-\theta_{j}$ for *i*, *j* being nearest-neighbor sites, while it is a *Z*_2_ spin liquid on a frustrated lattice (e.g., triangular lattice) (20). Fluctuating Kondo hybridizations play an essential role to suppress the spinon gap. As illustrated in Fig. 1*F*, the local spinon (*f*) in RVB singlets interacts with conduction electrons (*ψ*) via the composite holons (*χ*) through the fluctuating Kondo hybridization. The spinons gain kinetic energy by coupling to the fluctuating Kondo hybridizations (40). The total free energy of the system and consequently the spinon gap are reduced. At κ=1/2, a full suppression of the spinon gap is reached due to 1) particle–hole symmetry in the spinon (*f*) sector (*SI Appendix*, section S.IV), 2) the SU(*K* – 1) channel-symmetric fluctuating Kondo hybridization, and 3) the nature of the particle–particle pairing of fermionic Sp(*N*) RVB singlets. This is reminiscent of the Kondo-stabilized spin-liquid state in the context of the large-*N* mean-field approach to heavy-fermion superconductivity in ref. 40. The difference is that the spinons in ref. 40 couple to mean-field Kondo hybridization, while here they couple to Kondo fluctuations in the absence of Bose-condensed mean-field Kondo hybridization. This emergent unexplored quantum phase of matter is effectively a critical Bose–Fermi Kondo lattice system with gapless fermionic spinons and bosonic holons. This mechanism is generic and applicable to other types of 2D lattices where different spinon dispersions are expected to only quantitatively change the power-law exponents of the strange metal properties. Below, we discuss transport and thermodynamical observables in this NFL strange metal phase.

### Scattering *T* Matrix.

The conduction-electron *T* matrix is defined as $T(\omega )=\Sigma_{c}(\omega )/\left(1-G_{c0}(\omega )\Sigma_{c}(\omega )\right)$ with Σ*_c_* being the self-energy of the conduction-electron bath and $G_{c0}$ the Green’s function of the independent conduction bath (*Materials and Methods*). In the large-*N* limit, it reduces to $\Sigma_{c}(\omega )∼O(1/N)$ (*SI Appendix*, sections S.II and S.III). The *T* matrix provides insight into the local transport properties. It is proportional to the local density of states of the conduction bath and can be compared to the low-temperature scanning tunneling microscope measurement. By allowing the conduction electrons in the local baths to hop, the strange metal feature of the *T* matrix implies the same qualitative behavior in electrical resistivity $\Delta \rho (T)=\rho (T)-\rho_{0}$ with ρ(T) being the total resistivity and *ρ*_0_ being the residual resistivity at *T* = 0. In the strange metal phase, via the interplay of the (gapless) power-law spinon and holon spectral functions of Fig. 1*F*, we find that the static *T* matrix, corresponding to the scattering rate, $\tau^{-1}(\omega =0,T)=-NT^{\prime \prime }(\omega =0,\;T)$, shows a strange metal feature with superlinear-in-*T* power-law behavior

**Fig. 2.**
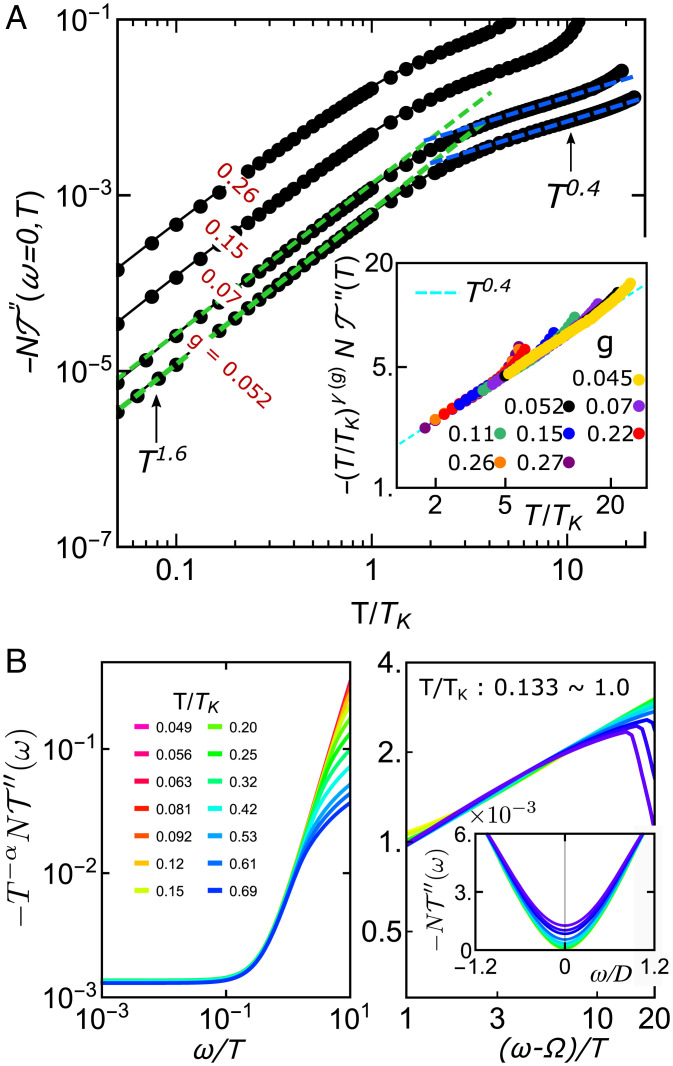
The scattering *T* matrix and its ω/T scaling. (*A*) *T* matrix with different values of *g* at κ=1/2. *Inset* shows scaling of *A* with γ(g) being a nonuniversal constant. (*B*) ω/T scaling of *T* matrix for ω/T<1 with α∼1.75 (*Left*) and for ω/T>1 with α=0.37 (*Right*) for *g* = 0.052. Ω is a fitting parameter. *Inset* shows the curves of the unscaled *T* matrix.

[4] $$-NT\prime \prime (\omega =0,T)\propto T^{1+p}\;\text{with}\;p\approx 0.6$$

as T→0 over a wide range in *g* and $T/T_{K}$ (Fig. 2*A*), signaling a NFL strange metal phase (yellow region in Fig. 1*A*). This power-law exponent is well accounted for by the power-law behavior of spinon and holon spectral functions for T→0 (Fig. 1*C* and *SI Appendix*, section S.IX). Moreover, this exotic phase shows a quantum critical nature, supported by the ω/T scaling of $-T^{-\alpha }NT^{\prime \prime }(\omega ,T)$ over a wide range in ω/T and *g* (Fig. 2*B* and *SI Appendix*, section S.IX):

[5] $$-T^{-\alpha }NT^{\prime \prime }(\omega ,T)\propto \Phi (\omega /T)$$

with Φ(ω/T) being a universal function that is shown in Fig. 2*B*. Here, we realize a distinct type of critical spin liquid with strange metal features mediated by the Kondo fluctuations. By contrast, the critical (gapless) spin-liquid state realized in AF Heisenberg models arises from magnetic frustration (44, 45). Furthermore, a NFL strange metal region (gray area in Fig. 1*A*) centered at the KB QCP ($g=g_{QC}$) at *T* = 0 shows features of the quantum critical region with a different NFL behavior in the *T* matrix, $-NT^{\prime \prime }(\omega =0,T)∼T^{1-p}$ with p≈0.6, associated with quantum-critical scaling (Fig. 2 *A*, *Inset*).

### Thermodynamical Observables.

**Fig. 3.**
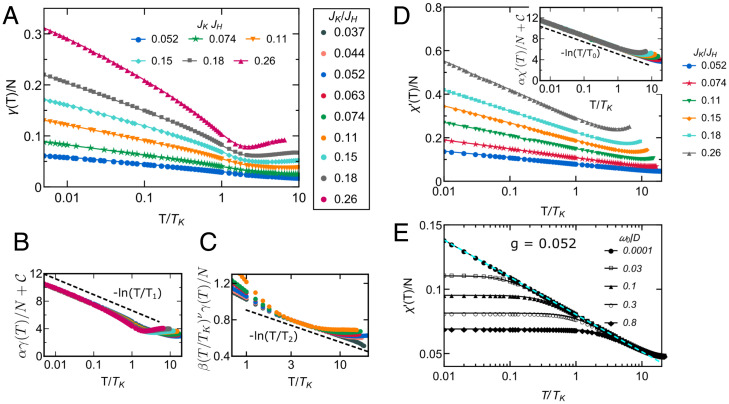
Specific heat coefficient, spin susceptibility, and their NFL strange metal behavior. (*A*) The specific heat coefficient is plotted as a function of $T/T_{K}$. (*B*) γ(T) of the low-*T* regime is rescaled and fitted to a *T*-logarithmic function (black dashed line). (*C*) γ(T) of the NFL strange metal region is rescaled and fitted to a *T*-logarithmic function (black dashed line). The plot keys for *B* and *C* are shown in *A*, *Right*. In *B* and *C*, *α*, *C*, *β*, C′, *T*_1_, and *T*_2_ are nonuniversal constants. (*D*) Static uniform spin susceptibility χ′(T)/N as a function of $T/T_{K}$. In *Inset*, χ′(T)/N of the low-*T* regime is rescaled and fitted to a *T*-logarithmic function (black dashed line). Here, *α*, *C*, and *T*_0_ are nonuniversal constants. (*E*) Dynamical uniform spin susceptibility $\chi \prime (\omega_{0},T)/N$ with different values of frequency *ω*_0_ (in units of half-bandwidth *D*) for $J_{K}/J_{H}=0.052$ fixed. The cyan dashed line is a fit to a *T*-logarithmic function.

We further find the strange metal phase also shows NFL behavior in thermodynamical quantities. The temperature-dependent specific heat coefficient γ(T) exhibits a logarithmic-in-*T* divergence both in the low-*T* limit and in the quantum critical regime (Fig. 3 *A*–*C*):

[6] $$\gamma (T)=-a\ln\;(T/T_{1,2}),$$

with *a* and $T_{1,2}$ being nonuniversal constants. Similar −ln (T) dependence is found in both static and dynamical spin susceptibility (Fig. 3 *D* and *E*). Interestingly, the dynamical spin susceptibility at a fixed frequency tends to saturate at low temperatures, reminiscent of the Pauli spin susceptibility (Fig. 3*E*), suggesting the fermionic nature of the critical spin liquid (47).

### The Quantum Critical Strange Metal Phase.

**Fig. 4.**
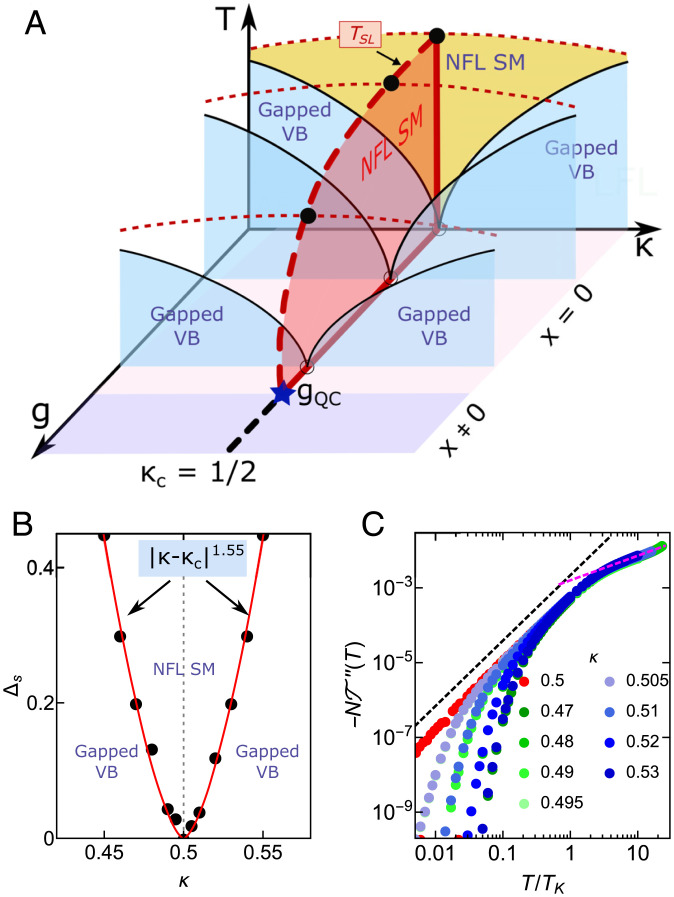
Strange metal features with different values of *κ*. (*A*) Schematic phase diagram as functions of *g*, *κ*, and *T* of our model. On the *κ*-*T* plane for fixed $0<g<g_{QC}$, the strange metal phase at $\kappa_{c}=1/2$ (black dashed line) is a QCP separating two gapped valence-bond (VB) phases for κ≶1/2. The quantum critical fan (yellow region) is centered at *κ_c_*, where the strange metal features extend over a finite region for $0<g<g_{QC}$ (the red shaded area bounded by the red dashed and solid lines). (*B*) Spinon gap Δ*_s_* versus *κ* for *g* = 0.052. The spinon gap on both sides of *κ_c_* exhibits a power-law behavior in $\left|\kappa -\kappa_{c}\right|$ (red curve), suggesting *κ_c_* is a QCP. (*C*) Plot of the *T* matrix for different values of *κ* near $\kappa_{c}=1/2$ with *g* = 0.052 fixed.

Since the strange metal phase shows NFL behaviors and dynamical ω/T scaling typically appearing near a QCP, we investigate further the existence and the nature of this alluded to QCP on more general grounds. As mentioned above, we find the strange metal phase is protected by both SU(*K* – 1) channel symmetry and particle–hole symmetry in the spinon (*f*) part of the Hamiltonian. In a more general parameter space of (*κ*, *g*), the strange metal phase is located at an unstable spinon (*f*) particle–hole and SU(*K* – 1) channel-symmetric QCP (19) at $\kappa_{c}=1/2$ for a given $g<g_{QC}$ (Fig. 4*A*). This QCP is then extended to a quantum critical line (or a quantum critical phase for $T<T_{SL}$) for $0<g<g_{QC}$ as *g* is tuned (the red shaded region bounded by the red solid and dashed *T_SL_* lines in Fig. 4*A*).

The stability of the strange metal phase against particle–hole asymmetry is analyzed below. We find our results are robust when the particle–hole symmetry of the conduction bath is broken (*SI Appendix*, section S.IX). For highly frustrated local spins with κ<1/2, which breaks particle–hole symmetry of *f* electrons, the system develops gaps (Δ*_s_*, Δ*_h_*) in both the spinon and holon spectral functions in the spin-liquid phase (Fig. 1*D*) where the temperature-dependent observables show an exponential decay as T→0 (*SI Appendix*, section S.IX). Moreover, we find $\Delta_{s,h}$ both vanish in a power-law fashion as *κ* approaches the QCP at $\kappa_{c}=1/2$ (Fig. 4*B*). The strange metal features in this phase extend to a finite range of the quantum critical fan at finite temperatures as *κ* is tuned slightly away from particle–hole symmetry (Fig. 4*A*), as indicated in the *T* matrix (Fig. 4*C*).

We make some remarks here. Although the existence of the strange metal phase requires the particle–hole symmetry of the spinons at $\kappa_{c}=1/2$, our results have broad applications. They are generic features of a large class of intermetallic compounds described by the *S* = 1/2 KH model and are robust against particle–hole asymmetry of the conduction band. These features also survive at finite temperatures even when the *f* spinons are away from the particle–hole symmetric point at $\kappa =\kappa_{c}$ (Fig. 4*A*). We discuss below the application of our results for the strange metal phase recently observed in a frustrated Kondo lattice compound and the implication of our results in the context of high-*T_c_* cuprate superconductors (*Discussion*).

### Benchmarking the Strange Metal Phase.

To demonstrate the unique particle–hole symmetry protected strange metal phase we find here for κ=1/2, we benchmark our results by comparing its singular NFL properties with that of the Jones–Varma fixed point in the two-impurity Kondo model (48, 49), also known to be sensitive to particle–hole asymmetry. At the Jones–Varma QCP of the two-impurity Kondo model, while the specific heat coefficient shows a similar −ln (T) divergence as observed in our strange metal phase, uniform spin susceptibility does not diverge as opposed to the *T*-logarithmic divergence in the strange metal phase. Moreover, the superlinear-in-*T* ($∼T^{1.6}$) electron scattering rate (associated with resistivity) in our strange metal phase is distinct from the two-channel Kondo-like $\sqrt{T}$-dependent resistivity found near the QCP of the two-impurity Kondo model in an AF-coupled double-quantum dot system (50), equivalent to the Jones–Varma QCP.

The above differences between the strange metal phase and the Jones–Varma QCP suggest that they correspond to distinct fixed points. In the context of renormalization group analysis, the Jones–Varma QCP of the two-impurity Kondo model and the quantum-critical strange metal phase we find here are indeed distinct fixed points and therefore belong to two different university classes although they both sensitively depend on particle–hole symmetry: The Jones–Varma QCP of the two-impurity Kondo model is an unstable critical fixed point by tuning both $g=J_{K}/J_{H}$ and the particle–hole asymmetry, while the strange metal phase consists of a line of fixed points (a phase instead of a single quantum critical “point”) that are stable along *g* for $0<g<g_{QC}$ but unstable against particle–hole asymmetry (Fig. 4*A*). Note that the sensitive dependence of the Jones–Varma QCP on particle–hole symmetry comes as a result of the specific form of the Kondo couplings, where the two impurity spins are coupled through the Kondo term to the same (single) conduction-electron bath. Consequently, any potential scattering term that destroys particle–hole symmetry will smear out the transition (51). A slightly different two-impurity Kondo model was studied in the context of a double quantum dot setup where two magnetic impurities couple independently to two separate conduction baths (50). Interestingly, the QCP therein is robust against particle–hole asymmetry, and it is unstable only against direct charge transfer between the impurities.

**Fig. 5.**
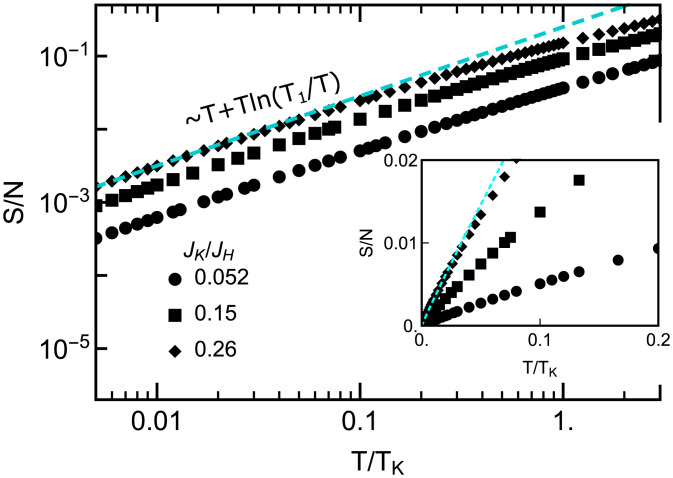
Entropy with different values of $J_{K}/J_{H}$ in the strange-metallic RVB spin-liquid phase of the channel-asymmetric large-*N* case. The entropy decreases as $T+T\ln\;(T_{1}/T)$ with decreasing temperature (the cyan dashed line). *Inset* displays the same plot in the linear scales.

Clear evidence confirming the distinction between the strange metal and Jones–Varma fixed points of the two-impurity Kondo model comes from the different values of the zero-temperature entropy *S*_0_ in these two cases. At the Jones–Varma QCP, one has $S_{0}=(1/2)\ln\;2$, identical to the residual entropy of a two-channel Kondo fixed point. By contrast, the entropy S(T)/N in the strange metal phase decreases as $S(T)/N\propto T+T\ln\;(T_{1}/T)$ with decreasing temperatures (Fig. 5). Due to the quantum critical nature of the strange metal phase, we expect the entropy will saturate at a value much smaller than (1/2)ln 2 in the T→0 limit. However, it goes beyond our present numerical capability to confirm this expectation. This quantum-critical strange metal phase is also different from the gapped-valence-bond (local spin-singlet) phase of the two-impurity Kondo model. The latter always has a finite spinon (holon) gap at the Fermi energy due to the local (zero-dimensional) nature of the dispersionless spinon. We find the key factor giving rise to the strange metal feature is the gapless fermionic spinon and bosonic holon excitations in a 2D critical spin-liquid phase.

As a consistency check, we apply our method to the two-impurity Kondo model. Similar to the lattice case, the resulting phase diagram contains four phases: a gapped-valence-bond (local spin-singlet) phase, a Kondo singlet phase, a decoupled phase, and a coexisting phase (*SI Appendix*, section S.XII). Within our numerical accuracy, the coexisting region can be largely suppressed by fine tuning *J_K_* and *J_H_*, leading to a QCP between the valence-bond phase and the Kondo singlet phase. We found a finite zero-temperature entropy slightly less than ln 2 around the critical value of $T_{K}/J_{H}$ (34) (*SI Appendix*, section S.XII), resembling the Jones–Varma fixed point of the two-impurity Kondo model. The QCP of our two-site problem shows distinct NFL behavior from that in the quantum-critical strange metal phase on the 2D lattice (*SI Appendix*, section S.XII), as expected. Note that, different from the Jones–Varma two-impurity Kondo model where two local-impurity spins share a single electron bath, our model assumes two independent electron baths, coupled separately to the two impurity spins. This setup forbids charge transfer between the two impurity sites, allowing the QCP to persist even without particle–hole symmetry (50).

### Application for CePd${}_{1-x}$Ni*_x_*Al.

The compound CePd${}_{1-x}$Ni*_x_*Al is known as a partially frustrated Kondo lattice system (12, 26–28, 52). The Ce atoms on the geometrically frustrated kagomé lattice show Kondo hybridization between the effective *S* = 1/2 local *f*-electron spins and that of mobile *d* electrons. In the absence of field and pressure, the system shows AF LRO below 2.7 K and an effective single-impurity Kondo temperature $T_{K}\approx 5$ K (53). Note that here *T_K_* is different from the coherent lattice Kondo temperature ($T^{*}$). Due to geometrical frustration, only two-thirds of spin moments participate in the magnetic order (52). The magnetic interactions are of the Ising type with AF couplings along the *c* axis and ferromagnetic (antiferromagnetic) couplings between nearest-neighbor (next-nearest-neighbor) spins in the *a*-*b* kagomé plane, respectively. Chemical substitution of Ni effectively introduces a positive pressure to the system.

With increasing field and pressure, the system undergoes two phase transitions: Due to frustration, the long-range magnetic order is first suppressed at a lower critical field ($B_{c1}$) and pressure ($p_{c1}$), leading to a paramagnetic metallic spin-liquid state (12, 27). At higher field and pressure, the system goes from a paramagnetic spin-liquid phase to a heavy Fermi-liquid phase via the KB transition, characterized by a small-to-large the Fermi surface volume jump as T→0 at a higher field ($B_{c2}$), observed in Hall coefficient measurement (12). This is reminiscent of the Fermi surface jump observed in YbRh_2_Si_2_ (9, 10). In the paramagnetic phase of pure CePdAl under fields, the alternating current (AC) susceptibility shows a pronounced increase upon cooling and a Pauli-like saturation at low temperatures, consistent with a fermionic spin liquid (12). The fermionic RVB spin-liquid state we propose here is a possible realization of it. In the paramagnetic spin-liquid phase, the features of a stable strange metal phase are observed, including quasilinear-in-*T* resistivity and −ln (T) dependence in specific heat coefficient as T→0 (27, 28).

Although magnetic interactions in the AF-ordered phase are three-dimensional, signatures of effective two-dimensional nature in specific heat coefficient have been observed in the absence of AF LRO, showing logarithmic divergence in $\left|B-B_{c1}\right|$ at the edge of antiferromagnetism (54) and *T*-logarithmic singularity in the frustration-induced spin-liquid phase (27, 54). Meanwhile, geometrical frustration lying in the kagomé plane plays a crucial role to give rise to the spin-liquid phase as suggested by the abovementioned spin-liquid behavior in AC spin susceptibility (12) and the enhanced low-temperature entropy (25) in this region, making the material in the absence of magnetic LRO an effective “two-dimensional” Kondo lattice system.

The conventional Hertz–Millis-type SDW theory is known to capture the magnetic transitions at the edge of the LRO phase, such as in itinerant ferromagnetic metals (4). The SDW in CePdAl was observed in the form of amplitude modulations of the Ising spins along the crystallographic *c* axis in the LRO phase (55). This scenario was reported to capture the magnetic transition at CePd${}_{1-x}$Ni*_x_*Al at $B_{c1}$ ($p_{c1}$) due to proximity to the magnetic long-range order (25, 27). However, the SDW scenario is unlikely to account for the dominating features of the strange metal phase and the paramagnetic spin-liquid-to-FL phase transition at $B_{c2}$ ($p_{c2}$) of this material for the following reasons: 1) The finite-temperature cross-over line in Fermi volume for a typical SDW QCP does not converge to the QCP at *T* = 0, leading to a smooth evolution of Fermi surface across the SDW transition (14, 56, 57), inconsistent with the KB scenario where the finite-temperature cross-over line merges with the QCP and the jump in Fermi volume at T→0 as was observed in CePd${}_{1-x}$Ni*_x_*Al (12). 2) The KB QCP occurring at $B_{c2}$ ($p_{c2}$) is detached from the AF LRO phase transition at $B_{c1}$ ($p_{c1}$). The SDW fluctuations, more relevant in the LRO phase and near $B_{c1}$ ($p_{c1}$), are expected to be strongly suppressed in the strange metal phase and near $B_{c2}$ ($p_{c2}$) where the critical charge fluctuations become essential. This expectation was demonstrated experimentally in Ge-substituted YbRh_2_Si_2_, where SDW is unable to explain the thermodynamic properties in the paramagnetic spin-disordered NFL strange metal state (30, 31). By contrast, the mechanism based on the KB scenario offers qualitative understanding of these phenomena (24). Nevertheless, since these two transitions are close by, SDW is expected to survive but to play only a subleading role in the strange metal phase and near $B_{c2}$ ($p_{c2}$).

Based on these observations, the interplay of Kondo and RVB spin liquid analyzed above offers an attractive mechanism for a qualitative understanding of the strange metal phase, despite the different type (Heisenberg as opposed to Ising) of AF coupling in our model. We expect that this difference may lead to a quantitative change in the power-law exponents of the strange metal phase. Note that critical exponents of the classical AF Ising model are the same as for the AF Heisenberg model (58). The ratio $g\equiv J_{K}/J_{H}$ is expected to increase with increasing field or pressure (24). The calculated strange metal feature in the *T* matrix (Eq. **4**) and the dynamical spin susceptibility are qualitatively in good agreement with the quasilinear-in-*T* resistivity persistent to the lowest temperature, observed in both its pure and Ni-doped forms (27), as well as the dynamical spin susceptibility measurement for its pure form (12, 26, 27), respectively. The *T*-logarithmic divergence in specific heat coefficient (Eq. **6**) we find in the strange metal phase bears a striking similarity to that observed near critical Ni doping (27, 28).

## Discussion

Due to the existence of an extended spinon Fermi surface, spinons are deconfined despite the U(1) gauge field in the spin-liquid dominating regions and strange metal phase (*SI Appendix*, section S.V) (59). Our results are therefore stable against U(1) gauge field fluctuations. However, a confining spin-liquid phase for κ<1/2 will give way to the translational symmetry-breaking valence-bond state due to the confining U(1) gauge force (Fig. 4*A*) (60). While spinon and holon Green’s functions are gauge dependent, physical observables are gauge-invariant combinations of these Green’s functions (*SI Appendix*, section S.VIII). We have checked that the qualitative features of the strange metal phase and quantum-critical strange metal regions in the large-*N* limit persist at finite *N* and *K*, including in the physical SU(2) ≡ Sp(2) (N=2, K=1) limit (*SI Appendix*, section S.XI). Note that, in the physical limit, following the way we decouple the Kondo hybridization (holon) field in the multichannel case (*Results*), the *χ_ia_* field in the single-channel case here is either a Bose-condensed mean field ($\chi_{ia}=x$) in the Kondo and coexisting phases or a pure fluctuating quantum field $\chi_{ia}={\hat{\chi }}_{ia}$ in the other phases or regions. While the local bath approximation corresponds to the single-site approximation of the self-energies of fields, it is already sufficient to effectively capture the important aspects of the KB QCP since the critical modes are dominated by local charge (Kondo) fluctuations (13, 38). When the channel symmetry is preserved ($J_{K}\prime =J_{K}$), we find the Kondo-screened Fermi-liquid and the coexisting phases are unstable against the overscreened NFL ground state (the decoupled phase), consistent with ref. 19 on the large-*N* approach to the multichannel single-impurity Kondo model. In this case, only the RVB spin-liquid strange metal and the decoupled phases are stable ground states. To describe the residual resistivity *ρ*_0_ as T→0 in realistic materials, one needs to also include scattering of conduction electrons by local defect (nonmagnetic) impurities, which are inevitably present there (61). In our case, the *T* matrix, proportional to the local scattering rate, depends on energy (or frequency *ω*) and not on momentum. The residual scattering rate in the *T* = 0 limit is just a constant added to the total scattering rate.

Our results shed light on the strange metal state in cuprate superconductors. The Fermi surface reconstruction has been observed near the optimal-doped cuprates where strange metal phenomena live (1). The presence of a small electron pocket slightly below the optimal doping has been interpreted as Fermi surface reconstruction induced by the charge-density-wave state, distinct from the SDW state that already vanishes at a lower doping (1, 62). This suggests a link between strange metal state and the (critical) charge fluctuations near Fermi surface reconstruction, reminiscent of the critical charge fluctuations near the KB transition of our system. While signatures of the QCP hidden inside the cuprate superconducting dome were reported (1, 62), a recent experiment reveals the strange metal regime persisting over a finite doping range as T→0 (63), indicating an exotic quantum-critical strange metal phase. Whether the strange metal state in cuprates is linked to a quantum critical point or a phase is under intense investigation (64). On theoretical grounds, it has been known that the *S* = 1/2 KH model is related to the *S* = 1/2 *t*-*J* model, appropriate for describing cuprates (65, 66). In particular, the authors in ref. 66 map the slave-boson *t*-*J* model onto an effective KH model, where the hoping (*t*) term (Heisenberg *J* term) of the *t*-*J* model is equivalent to the Kondo (Heisenberg) term of the KH model, respectively. At the mean-field level, this effective KH Hamiltonian supports the pseudogap, strange metal, superconducting, and normal FL phases. Within this effective KH model, the system can undergo a phase transition from the strange metal phase to a normal FL metal phase via a “KB”-like transition in the context of critical charge fluctuations and Fermi surface reconstructions. This transition is expected to be characterized by Bose condensing the slave bosons of the hoping term (or the effective Kondo term) in the *t*-*J* model, leading to the jump in Fermi volume from a smaller value in the strange metal phase to a larger value in the Fermi liquid metal phase. Our approach and results therefore offer a unique perspective to address this well-known problem.

Before we summarize, we discuss a few issues when comparing our model and results to CePdAl. First, due to the lack of inversion symmetry and the existence of spin–orbit coupling of Ce atoms, Dzyaloshinskii–Moriya interaction (DMI) in principle exists in this material with the following Hamiltonian: $D_{ij}\cdot (S_{i}\times S_{j})$, where the $D_{ij}$ vector is related to the spin–orbit coupling. However, due to the Ising-like local spins whose spin moments are predominantly parallel to each other, the DMI is therefore strongly suppressed since $S_{i}\times S_{j}\ll 1$. Nevertheless, we expect that the DMI does exist but plays only a subleading role here since 1) a strong magnetic anisotropy from the spin susceptibility measurements for B⊥c and B∥c is present (28, 67), indicating the material is not a pure Ising model system although it is almost Ising-like, and 2) the admixture of doublet ground states of *J* = 5/2 Ce atoms implies a finite spin–orbit coupling (68), giving rise to $D_{ij}\neq 0$. To date, there is no experimental report on the existence of DMI in CePd${}_{1-x}$Ni*_x_*Al. Further experimental confirmation on the signatures of DMI is needed. Second, the splitting of the energy levels due to the crystal electric effect (CEF) for CePdAl has been observed at a rather high temperature, *T*  >  240 K (69). However, we checked that, based on the realistic estimation of Kondo coupling $\rho J_{K}\approx 0.3$ and bandwidth D∼0.2 eV (70), the Kondo temperature is estimated as $T_{K}=D\exp\;\left(-2/\rho J_{K}\right)\approx 3$ K, giving rise to the cross-over temperature $T^{*}\approx 30T_{K}\approx 90$ K of the Kondo phase to occur at a temperature well below the temperature scale of CEF effect T∼240 K. Therefore, we do not expect significant modifications for our results due to CEF splitting. The estimated Kondo temperature $T_{K}\approx 3$ K here is in reasonable agreement with experiments in ref. 53, where $T_{K}\approx 5$ K at ambient pressure. Finally, the predicted coexisting superconducting phase in our model calculations has not been observed in this material although it commonly appears near a QCP in various fermionic large-*N* approaches to the KH lattice models (20, 42, 71). We think the possible explanations for this discrepancy are the following: 1) The size of the coexisting region we find here is overestimated since the fluctuations of RVB spin-liquid order parameter Δ are not included in our calculations. By including these fluctuations, the cross-over temperature of the coexisting phase is expected to get further reduced, and a full suppression of the coexisting region may be possible. 2) The material may show superconductivity at a much lower temperature than the temperature range in the existing experiments. For example, the superconducting phase in YbRh_2_Si_2_ was recently discovered at a much lower temperature range (72) than that in previous measurements where superconductivity was not observed for more than a decade (73). Meanwhile, we further explored the phase diagram in a wider range in the multidimensional parameter space of ($T,\;J_{K}^{a},\;J_{H}$). We find that the size of this coexisting phase in our model calculations can be varied. It can be sizable, negligible, or even fully suppressed, depending on the individual values of Kondo and Heisenberg couplings and how these couplings are approached to the KB QCP. Our phase diagram in Fig. 1*A* is obtained with channel-asymmetric Kondo couplings $J_{K}^{\prime }=2J_{K}$ by fixing $J_{K}/D=1$ while changing the value of *J_H_*. However, when *J_K_* and *J_H_* are tuned in a different way across the KB QCP, we find a negligible and even full suppression of the coexisting region with further decreasing channel asymmetry to $J_{K}\prime /J_{K}\to 1^{+}$ (*SI Appendix*, section S.XII). Although it is not clear how the Kondo and Heisenberg couplings are varied in experiments of CePd${}_{1-x}$Ni*_x_*Al across the KB QCP, by fine-tuning $J_{K}\prime /J_{K}$ and $J_{K}/J_{H}$, we do find a full suppression of coexisting phase. More importantly, the qualitative features of our main results on the strange metal non-Fermi liquid phase in the RVB spin-liquid–dominated region are generic, robust, and relevant for a broad range of rare-earth intermetallic compounds regardless of the existence or the size of the coexisting superconducting phase.

In summary, by constructing a controlled dynamical large-*N* approach to the 2D Kondo–Heisenberg model, we have identified a mechanism of realizing a strange metal phase applicable to paramagnetic rare-earth intermetallic compounds. This phase is stabilized by the interplay of the short-range antiferromagnetic spin-liquid and critical charge (Kondo) fluctuations near the Kondo breakdown quantum critical point. We clarify the nature of this phase in terms of the fluctuating Kondo scattering-stabilized quantum critical spin-liquid metal. The quantum critical nature of this phase is manifested in ω/T scaling of the dynamical electron scattering rate. Our results in quasilinear-in-temperature conduction-electron scattering rate and logarithmically in-temperature divergent specific heat coefficient as T→0 were recently observed in CePd${}_{1-x}$Ni*_x_*Al. Our results serve as a basis of realizing emergent quantum critical strange metal phases in correlated electron systems in general.

## Materials and Methods

### The Large-*N* Multichannel Generalization of the Kondo Lattice Model.

Starting from Eq. **1**, the *H_K_* and *H_J_* in the single-channel physical SU(2) limit can be written as

[7] $$\begin{array}{l} H_{K}=\left(-\frac{J_{K}}{2}\right)\sum_{i,\alpha ,\beta }\psi_{i\alpha }^{†}f_{i\alpha }f_{i\beta }^{†}\psi_{i\beta },\\ H_{J}=\left(-\frac{J_{H}}{2}\right)\sum_{\langle i,j\rangle ,\alpha \beta }\left(\tilde{\alpha }f_{i\alpha }^{†}f_{j,-\alpha }^{†}\right)\left(\tilde{\beta }f_{j,-\beta }f_{i\beta }\right), \end{array}$$

where α,β=±1 are the SU(2) spin indexes and $\tilde{\alpha }(\tilde{\beta })$ is defined in Eq. **2**. The bath lattice is orthogonal to the impurity lattice. We then generalize Eq. **7** to the large-*N* channel-asymmetric multichannel KH model by allowing *N* spin flavors (α,β=±1,⋯,±N/2) and *K* Kondo screening channels ($\psi_{i\alpha }\to \psi_{i\alpha a}$) with a=1,⋯,K and channel-dependent Kondo coupling $J_{K}^{a}$, as shown in Eq. **2** (19, 33). Next, we introduce two auxiliary bosonic fields, the Kondo hybridization (holon) field *χ_ia_* and the RVB spin-singlet pairing field Δ*_ij_*, to factorize *H_K_* and *H_J_* of Eq. **2** via Hubbard–Stratonovich transformation (see *SI Appendix*, section S.I for details). This generalized large-*N* channel-asymmetric multichannel KH model with $J_{K}^{a=K}\equiv J_{K}\prime >J_{K}^{a<K}\equiv J_{K}$ shows a total symmetry of Sp(*N*) × SU(*K* – 1). The effective action of this model reads (see *SI Appendix*, section S.I for derivations)

[8] $$\begin{array}{l} S=-\sum_{ka\alpha }\psi_{ka\alpha }^{*}G_{c0}^{-1}(k)\psi_{ka\alpha }-\sum_{k\alpha }f_{k\alpha }^{*}\left[i\omega +\lambda -x^{2}G_{c0}(k)\right]f_{k\alpha }\\ +\beta NN_{s}\left(\frac{2\Delta^{2}}{J_{H}}+\lambda \frac{K}{N}+\frac{x^{2}}{J_{K}\prime }\right)+\sum_{k,a}\frac{{|{\hat{\chi }}_{ka}|}^{2}}{J_{K}}\\ +[\frac{x}{\sqrt{N}}\sum_{ka\alpha }f_{k\alpha }^{*}\psi_{ka\alpha }+\frac{1}{\sqrt{N}}\sum_{kp\alpha a}{\hat{\chi }}_{p-k,a}f_{p\alpha }^{*}\psi_{ka\alpha }\\ +\Delta \sum_{k,\alpha }\tilde{\alpha }f_{k\alpha }f_{-k,-\alpha }\xi_{k}+\text{c}.\text{c}.], \end{array}$$

where a∈[1,K−1] and $k\equiv (\omega_{n},\;k)$ with *ω_n_* being the Matsubara frequency. In Eq. **8**, $G_{c0}(i\omega )=\sum_{P}{\left(i\omega -\varepsilon_{P}\right)}^{-1}$ is the bare Green’s function of local electron baths. Here, *N_s_* denotes the number of impurity lattice sites, the Lagrange multiplier *λ* is imposed to capture the local constraint of $f_{i\alpha },\;\langle n_{f}(i)\rangle =Q$, and *x* represents the Bose-condensed part of the Kondo hybridization field *χ_ia_*. Also, Δ represents the uniform mean-field value of the RVB spin-singlet pairing field Δ*_ij_*: $\Delta =(J_{H}/N)\sum_{p\alpha }\text{sgn}(\alpha )\xi_{p}\langle f_{p\alpha }^{†}f_{-p,-\alpha }^{†}\rangle$ with extended *s*-wave form factor $\xi_{p}=\cos\;p_{x}+\cos\;p_{y}$.

### Self-Consistent Equations.

In the large-*N* limit, Eq. **8** is solved by a set of self-consistent equations. Due to the independent electron bath approximation, the *χ_ia_* field is completely local; it therefore gives rise to the momentum-independent self-energies, and only the momentum-integrated Green’s functions are involved (*SI Appendix*, section S.II):

[9] $$\begin{array}{l} G_{\chi }(i\nu )={\left[-J_{K}^{-1}-\Sigma_{\chi }(i\nu )\right]}^{-1},\\ G_{f}(i\omega )=\sum_{p}\frac{\gamma (-i\omega )}{\gamma (i\omega )\gamma (-i\omega )+4\Delta_{p}^{2}}, \end{array}$$

where $\gamma (i\omega )\equiv i\omega +\lambda -|x{|}^{2}G_{c0}(i\omega )-\Sigma_{f}(i\omega )$ and $\Delta_{p}\equiv \Delta \xi_{p}$ is the form factor of the extended *s*-wave pairing. Note that this local approximation is sufficient to effectively capture important aspects of the local KB QCP since the critical modes are dominated by local charge (Kondo) fluctuations (13, 23, 32, 38). By neglecting the O(1/N) vertex corrections (*SI Appendix*, section S.II), the leading O(1) Dyson–Schwinger equations for the self-energies read

[10] $$\begin{array}{l} \Sigma_{\chi }(i\nu )=\sum_{\omega }G_{f}(i\omega +i\nu )G_{c0}(i\omega ),\\ \Sigma_{f}(i\omega )=-\kappa \sum_{\nu }G_{\chi }(i\nu )G_{c0}(i\omega -i\nu ). \end{array}$$

The self-energy of the conduction electrons is of order O(1/N) and is therefore neglected in the large-*N* limit.

We solve the Green’s functions Eq. **9** and self-energies Eq. **10** self-consistently subject to the saddle-point equations of the three mean-field variables λ, Δ, x through minimizing the free energy of Eq. **8** with respect to λ, Δ, x. We provide the details of the saddle-point equations in *SI Appendix*, section S.II.

## Supplementary Material

Supplementary File

## Data Availability

Original data and codes created for the study have been deposited in the online Zenodo repository (DOI: 10.5281/zenodo.5914650).
