# Supplementary material for: A mechanism for the strange metal phase in rare-earth intermetallic compounds
Source: Proc Natl Acad Sci U S A. 2022 Mar 1;119(10):e2116980119. doi: 10.1073/pnas.2116980119 (PMC8915960; doi:10.1073/pnas.2116980119)
Supplement: Supplementary File [file pnas.2116980119.sapp.pdf]

Supplementary Information for

A mechanism for the strange metal phase in rare-earth inter-metallic compounds

Jiangfan Wang, Yung-Yeh Chang and Chung-Hou Chung

Corresponding Author: Chung-Hou Chung
E-mail: chung0523@nycu.edu.tw

This PDF file includes:

Figs. S1 to S23 (not allowed for Brief Reports)
SI References

In this Supplementary Information, we provide additional details and calculations that are not included in the main text.

S.I. Action

In this section, we define the action of the large-N multichannel Kondo-Heisenberg (KH) Hamiltonian H with the pseudofermion representation of impurity spins under the local bath approximation. The model Hamiltonian H is defined as

$$
\begin{aligned}
H &= \sum_i H_0(i) + \sum_i H_K(i) + \sum_{\langle ij\rangle} H_J(i,j),\\
H_0(i) &= \sum_{\boldsymbol{P}\alpha} \varepsilon_{\boldsymbol{P}} \psi^\dagger_{i\alpha}(\boldsymbol{P})\psi_{i\alpha}(\boldsymbol{P}),\\
H_K(i) &\equiv J_K \mathbf{S}_i \cdot \mathbf{s}^c_i, \quad H_J(i,j) \equiv J_H \mathbf{S}_i \cdot \mathbf{S}_j.
\end{aligned}
\tag{S1}
$$

Here, $\psi^\dagger_{i\alpha}(\boldsymbol{P})$ creates a conduction electron within the local electron bath that only couples to the impurity spin ($\boldsymbol{S}_i$) at site i. Each local bath is defined on an "artificial lattice" with momentum $\boldsymbol{P}$, which is orthogonal to the real 2D lattice labeled by i. $\varepsilon_{\boldsymbol{P}}$ is the electron dispersion within each local bath. The conduction electron spin density is defined as $\boldsymbol{s}^c_i = \frac{1}{2}\sum_{\alpha\beta}\psi^\dagger_{i\alpha}\boldsymbol{\sigma}_{\alpha\beta}\psi_{i\beta}$, where $\psi_{i\alpha} \equiv \sum_{\boldsymbol{P}}\psi_{i\alpha}(\boldsymbol{P})$ is the local electron annihilation operator, and α and β are the spin indices.

Before we proceed, it is worthwhile of justifying the inclusion of the antiferromagnetic RKKY interaction H_J in our model under the framework of local bath approximation and the large-N limit. Firstly, we would like to point out the ordering of our theoretical analysis. We start from the general Kondo-Heisenberg Hamiltonian [see, for instance, Eq. (1) of Ref. (1)], where the electron baths are all connected and the RKKY coupling is already present (or generated). We then approximate the Kondo effect with local bath approximation and solve the problem in the large-N limit. Note that the RKKY interaction is generated via Kondo coupling perturbatively before the local bath approximation is imposed (see explanations in the next paragraph). It is a well-defined model and procedure to make approximation; it has been widely applied in the literature of heavy-fermion Kondo lattice problem. See, for example, Refs. (2–4), and the Supplementary Methods of Ref. (5). In other words, when the local bath approximation is imposed, the RKKY coupling is already present.

To be more specific, one may think of the RKKY term H_J arising from a more general Hamiltonian $H_0 + H_K$ through integrating out the additional conduction electron degrees of freedom. Equivalently, one can imagine that we employ a Wilson renormalization group (RG) procedure on the Hamiltonian $H_0 + H_K$. By reducing the RG cut-off scale, one generates the RKKY coupling. To facilitate further discussion of the phase diagram, in the literature (see Ref. (1)) as well as here, the RKKY coupling is treated as an independent coupling since it is more convenient to tune the ratio J_H/J_K. It accesses the quantum phase transition that otherwise would have been induced in the tuning of J_H/J_K through the variation of the ratio of J_K to the conduction electron bandwidth. One needs to be sure that there is no double counting of the explicit and generated contributions to J_H, and this can be consistently done in practice. For a technical discussion of this point, see Ref. (6).

Meanwhile, we are also aware of the disadvantage of the large-N approach regarding the RKKY interaction. Within the large-N theory, the RKKY interaction is a sub-leading term relative to the Kondo effect since the RKKY coupling J_H obeys $J_H \sim J_K^2\rho_0 \sim O(1/N^2)$ via second-order perturbation in J_K. As a result, J_H is negligible in the large-N limit (see, for example, Ref. (7)). Nevertheless, as we argue above, on a general ground, the Kondo-Heisenberg Hamiltonian is a well-defined model Hamiltonian in its own right to qualitatively describe the quantum phases and phase transitions associated with the heavy-fermion systems. The large-N approach to Kondo-Heisenberg model has been extensively studied in the literature. At the level of model calculations, in the literature (see Ref. (1)) as well as here, the Kondo and Heisenberg coupling constants are treated as two independent coupling constants, which survive in the large-N limit. Alternatively, the Heisenberg term in our Hamiltonian can be also derived from the Hubbard-U term of the f-electrons by projecting out the empty and doubly occupied states, in parallel to the derivation of t-J model from Hubbard model. In this sense, we can circumvent this disadvantage.

We use Abrikosov pseudofermions to represent the local spin, $\boldsymbol{S}_i = \frac{1}{2}f^\dagger_{i\alpha}\boldsymbol{\sigma}_{\alpha\beta}f_{i\beta}$. At the same time, we allow the conduction electrons to have an additional channel index, $a = 1, \cdots, K$. Then, we have the following multichannel large-N extension of the three terms of Eq. (S1):

$$
\begin{aligned}
H_0 &= \sum_{i\boldsymbol{P}a\alpha} \varepsilon_{\boldsymbol{P}}\psi^\dagger_{ia\alpha}(\boldsymbol{P})\psi_{ia\alpha}(\boldsymbol{P}),\\
H_K &= \left(-\frac{1}{N}\right)\sum_{ia\alpha\beta} J^a_K \psi^\dagger_{ia\alpha} f_{i\alpha} f^\dagger_{i\beta}\psi_{ia\beta},\\
H_J &= \left(-\frac{J_H}{N}\right)\sum_{\langle i,j\rangle,\alpha\beta}\left(\tilde{\alpha} f^\dagger_{i\alpha} f^\dagger_{j,-\alpha}\right)\left(\tilde{\beta} f_{j,-\beta} f_{i\beta}\right).
\end{aligned}
\tag{S2}
$$

Here, the Heisenberg term has been written in a Sp(N)-invariant form with α, $\beta = \pm 1, \cdots, \pm N/2$ and $\tilde{\alpha} = \mathrm{sgn}(\alpha)$. In addition, we allow the Kondo coupling (J^a_K) to explicitly depends on channel. A previously studied multichannel large-N Kondo model corresponds to channel-symmetric Kondo couplings (8), i.e., $J^a_K = J_K$. The Kondo term therefore shows a SU(K)

channel symmetry. However, such channel-symmetric large-N theory will always give rise to non-Fermi liquid ground states associated with overscreened Kondo effects, as studied in Ref. (8). In the sense of RG, the overscreened Kondo fixed point is unstable against an infinitesimal channel asymmetry. Therefore, in order to obtain a fully Kondo screened Fermi liquid ground state, we explicitly break the SU(K) channel symmetry down to SU($K-1$) by assuming $J_K^{a=K} = J'_K$, $J_K^{a\neq K} = J_K$ with $J'_K > J_K$. Our main results in the main text focus on the special case $J'_K = 2J_K$. We should further introduce a Lagrange multiplier term to ensure the local constraint of the f fermions, $\sum_i \lambda_i(\sum_\alpha f^\dagger_{i\alpha} f_{i\alpha} - Q)$. In principle, Q and K are two independent parameters both scaled with N. In this paper, we intentionally choose $Q = K \equiv \kappa N$ as a mathematical simplification to reduce the parameter space.

Via Hubbard-Stratonovich transformation, we introduce two auxiliary boson fields, χ and Δ to decompose H_K and H_J:

$$H_K \to \sum_{ia}\left(\frac{1}{\sqrt{N}}\sum_\alpha \chi_{ia} f^\dagger_{i\alpha}\psi_{ia\alpha} + H.c. + \frac{|\chi_{ia}|^2}{J_K^a}\right),$$
$$H_J \to \sum_{\langle i,j\rangle}\left(\sum_\alpha \tilde{\alpha}\Delta_{ij} f_{j,-\alpha} f_{i\alpha} + H.c. + \frac{N|\Delta_{ij}|^2}{J_H}\right), \tag{S3}$$

where χ_i and Δ_{ij} characterize the Kondo hybridization field and the singlet RVB between adjacent spins, respectively. Due to the local constraint, the theory has a U(1) gauge symmetry associated with the following gauge transformation:

$$f_{i\alpha} \to f_{i\alpha} e^{i\theta_i}, \quad \chi_{ia} \to \chi_{ia} e^{i\theta_i},$$
$$\Delta_{ij} \to \Delta_{ij} e^{-i(\theta_i+\theta_j)}, \quad \lambda_i \to \lambda_i - i\partial_\tau \theta_i. \tag{S4}$$

To capture the paramagnetic Kondo-screened Fermi liquid and the antiferromagnetic states, we allow χ_i and Δ_{ij} to get Bose-condensed. In this work, we consider the case of extended s-wave pairing for the AF short-ranged RVB order parameter Δ_{ij} in Eq. (S3), corresponding to $\Delta_{i,i+\hat{x}} = \Delta_{i,i+\hat{y}} = \Delta$. This leads to the following decomposition of H_J:

$$H_J \to \Delta \sum_{\boldsymbol{k},\alpha}(\tilde{\alpha} f_{\boldsymbol{k}\alpha} f_{-\boldsymbol{k},-\alpha}\xi_{\boldsymbol{k}} + H.c.) + \frac{2N_s N|\Delta|^2}{J_H} \tag{S5}$$

with $\xi_{\boldsymbol{k}} = \cos k_x + \cos k_y$ and N_s is the number of sites. The fluctuation of Δ is of order of $O(1/N)$, hence not considered in our calculations.

Based on our assumption of channel-asymmetric Kondo coupling ($J_K^{a=K} = J'_K > J_K^{a\neq K} = J_K$), we allow for a Bose condensation of the Kondo hybridization field χ_{ia} on the $a = K$ channel, namely

$$\chi_{ia}(\tau) \to x\sqrt{N}\delta_{a,K} + \hat{\chi}_{ia}(\tau)(1-\delta_{a,K}). \tag{S6}$$

Here $x\sqrt{N}$ is the static uniform condensation of χ_{ia}. Unlike the Δ field, the fluctuation of χ_{ia} is of order of unity due to the additional $K-1$ channels. Consequently, the Kondo term reads

$$H_K \to \frac{1}{\sqrt{N}}\sum_{a=1}^{K-1}\sum_{i,\alpha}[\hat{\chi}_{ia} f^*_{i\alpha}\psi_{ia\alpha} + H.c.] + \sum_{a=1}^{K-1}\sum_i \frac{|\hat{\chi}_{ia}|^2}{J_K} + \left[x\sum_{i,\alpha} f^*_{i\alpha}\psi_{i,a=K,\alpha} + H.c.\right] + \beta N_s N\frac{x^2}{J'_K}. \tag{S7}$$

Now, the K-th channel of H_K is quadratic, thus we can integrate out $\psi_{iK\alpha}$. The effective action of the remaining $K-1$ channels after integrating out $\psi_{iK\alpha}$, shown in Eq. (8) of the main text, is written as $\mathcal{S} = \mathcal{S}_0 + \mathcal{S}'$ with $\mathcal{S}_0$ being the mean-field part, given by

$$\mathcal{S}_0 = -\sum_{ka\alpha}\psi^*_{ka\alpha} G^{-1}_{c0}(k)\psi_{ka\alpha} - \sum_{k\alpha} f^*_{k\alpha}\left[i\omega + \lambda - |x|^2 G_{c0}(k)\right] f_{k\alpha} + \left[\Delta^*\sum_{k,\alpha}\tilde{\alpha} f_{k\alpha} f_{-k,-\alpha}\xi_{\boldsymbol{k}} + H.c.\right]$$
$$+\beta N N_s\left(\frac{2|\Delta|^2}{J_H} + \lambda\frac{K}{N} + \frac{|x|^2}{J'_K}\right), \tag{S8}$$

where $a \in [1,\, K-1]$ in Eq. (S8), $k \equiv (\omega_n,\, \boldsymbol{k})$ with ω_n being the Matsubara frequency, and a uniform Lagrange multiplier $\lambda_i = \lambda$ has been assumed. The fluctuating part of the action is given by

$$\mathcal{S}' = \sum_{k,a\in[1,\cdots,K-1]}\frac{|\hat{\chi}_{ka}|^2}{J_K} + \frac{1}{\sqrt{N}}\sum_{k,p,\alpha}\sum_{a\in[1,\cdots,K-1]}\hat{\chi}_{p-k,a} f^*_{p\alpha}\psi_{ka\alpha} + \text{H.c.}. \tag{S9}$$

The partition function of the model is written as

$$Z = \int \mathcal{D}[\psi, f, \hat{\chi}] e^{-\mathcal{S}[\psi,f,\hat{\chi},\lambda,\Delta,x]}, \tag{S10}$$

where the mean-field variables λ, Δ, x are determined by the saddle point equations,

$$\frac{\partial \ln Z}{\partial \lambda} = \frac{\partial \ln Z}{\partial \Delta} = \frac{\partial \ln Z}{\partial x} = 0. \tag{S11}$$

S.II. Derivation of self-consistent equations

In this section, we give a detailed derivation of the large-N, K self-consistent Dyson-Schwinger equations, similar to that within the non-crossing approximation (NCA) (9), via taking functional derivatives of the action with respect to the external source fields.

We first introduce three external source fields, I, J, K to the effective action Eq. (8) of the main text. The partition function with the inclusion of the source fields is expressed as $Z[I,J,K]=\int_{[\psi,f,\hat{\chi}]}\exp\left[-\mathcal{S}+\tilde{\mathcal{S}}\right]$, where $\tilde{\mathcal{S}}\equiv\psi^\dagger\cdot I+f^\dagger\cdot J+\hat{\chi}^\dagger\cdot K+c.c.$ is the source term. Here, $\psi^\dagger\cdot I$ stands for $\sum_{\boldsymbol{p},\omega,a,\alpha}\psi^*_{\boldsymbol{p}\omega a\alpha}I_{\boldsymbol{p}\omega a\alpha}$, similar for $f^\dagger\cdot J$ and $\hat{\chi}^\dagger\cdot K$.

The Dyson-Schwinger equations are obtained by taking functional derivatives of the equations of motion (obtained by $0=\frac{\delta Z}{\delta\psi}=\frac{\delta Z}{\delta\hat{\chi}}=\frac{\delta Z}{\delta f}=\frac{\delta Z}{\delta f^*}$) with respect to the source fields, with the results:

$$\begin{aligned}1=&G_{c0}^{-1}(i\omega)G_c(\boldsymbol{p},i\omega)+\frac{1}{\sqrt{N}}\sum_{\boldsymbol{k},\nu}\left\langle f_{\boldsymbol{p}+\boldsymbol{k},\omega+\nu,\sigma}\hat{\chi}^*_{\boldsymbol{k}\nu a}\psi^*_{\boldsymbol{p}\omega a\sigma}\right\rangle,\\ 1=&-\frac{G_\chi(\boldsymbol{p},i\nu)}{J_K}+\frac{1}{\sqrt{N}}\sum_{\boldsymbol{k},\omega,\sigma}\left\langle\psi^*_{\boldsymbol{k}\omega a\sigma}f_{\boldsymbol{p}+\boldsymbol{k},\omega+\nu,\sigma}\hat{\chi}^*_{\boldsymbol{p}\nu a}\right\rangle,\end{aligned}\tag{S12}$$

and

$$\begin{aligned}1=&\left[i\omega+\lambda-x^2G_{c0}(i\omega)\right]G_f(\boldsymbol{p},i\omega)-2\Delta\xi_{\boldsymbol{p}}F_f(\boldsymbol{p},i\omega)+\frac{1}{\sqrt{N}}\sum_{\boldsymbol{k},\nu,a}\left\langle\hat{\chi}_{\boldsymbol{k}\nu a}\psi_{\boldsymbol{p}-\boldsymbol{k},\omega-\nu,a\sigma}f^*_{\boldsymbol{p}\omega\sigma}\right\rangle,\\ 0=&\left[-i\omega+\lambda-x^2G_{c0}(-i\omega)\right]F_f(\boldsymbol{p},i\omega)+2\Delta\tilde{\alpha}\xi_{\boldsymbol{p}}G_f(\boldsymbol{p},i\omega)-\frac{1}{\sqrt{N}}\sum_{\boldsymbol{k},\nu,a}\tilde{\alpha}\left\langle\psi^*_{-\boldsymbol{p}-\boldsymbol{k},-\omega-\nu,a,-\sigma}\hat{\chi}^*_{\boldsymbol{k}\nu a}f^*_{\boldsymbol{p}\omega\sigma}\right\rangle.\end{aligned}\tag{S13}$$

Here, the Green's functions are defined as

$$\begin{aligned}G_c(\boldsymbol{k},i\omega)=&-\langle\psi_{\boldsymbol{k}\omega a\alpha}\psi^*_{\boldsymbol{k}\omega a\alpha}\rangle,\\ G_f(\boldsymbol{k},i\omega)=&-\langle f_{\boldsymbol{k}\omega\alpha}f^*_{\boldsymbol{k}\omega\alpha}\rangle,\\ F_f(\boldsymbol{k},i\omega)=&-\tilde{\alpha}\langle f^*_{\boldsymbol{k}\omega\alpha}f^*_{-\boldsymbol{k}-\omega-\alpha}\rangle,\\ G_\chi(\boldsymbol{k},i\omega)=&-\langle\hat{\chi}_{\boldsymbol{k}\omega a}\hat{\chi}^*_{\boldsymbol{k}\omega a}\rangle.\end{aligned}\tag{S14}$$

The three-field correlators in Eqs. (S12) and (S13) can be written as simple multiplications of Green's functions due to the absence of vertex corrections at large-N. For example, the three-field correlator in the second line of Eq. (S12) can be written as

$$\frac{1}{\sqrt{N}}\sum_{\boldsymbol{p}\nu}\langle\hat{\chi}^*_{\boldsymbol{p}-\boldsymbol{k},\nu-\omega,a}f_{\boldsymbol{p}\nu\alpha}\psi^*_{\boldsymbol{k}a\omega\alpha}\rangle=\frac{-1}{N}\sum_{\boldsymbol{p}\nu\alpha'}G_\chi(\boldsymbol{p}-\boldsymbol{k},\nu-\omega)G_f(\boldsymbol{p},\nu)G_c(\boldsymbol{k},\omega).\tag{S15}$$

In this way, Eq. (S12) is reduced to

$$\begin{aligned}G_c^{-1}(\boldsymbol{p},i\omega)&=G_{c0}^{-1}(i\omega)-\Sigma_c(\boldsymbol{p},i\omega),\\ G_\chi^{-1}(\boldsymbol{p},i\nu)&=-J_K^{-1}-\Sigma_\chi(\boldsymbol{p},i\nu),\end{aligned}\tag{S16}$$

where

$$\begin{aligned}\Sigma_c(\boldsymbol{p},i\omega)\equiv&-\frac{1}{N}\sum_{\boldsymbol{k},\nu}G_\chi(\boldsymbol{k},i\nu)G_f(\boldsymbol{p}+\boldsymbol{k},i\omega+i\nu),\\ \Sigma_\chi(\boldsymbol{p},i\nu)\equiv&\sum_{\boldsymbol{k},\omega}G_f(\boldsymbol{p}+\boldsymbol{k},i\omega+i\nu)G_c(\boldsymbol{k},i\omega).\end{aligned}\tag{S17}$$

Since $\Sigma_c\propto\mathcal{O}(N^{-1})$, the self-energy of the c-electrons can be ignored in the large-N limit. Therefore, we replace $G_c(\boldsymbol{k},i\omega)$ by $G_{c0}(i\omega)$ in our calculations. As a result, G_χ becomes local:

$$G_\chi^{-1}(i\nu)=-J_K^{-1}-\Sigma_\chi(i\nu),\tag{S18}$$

where we denote the local self-energy of the χ field as $\Sigma_\chi(i\nu)\equiv\sum_\omega G_f(i\omega+i\nu)G_{c0}(i\omega)$, with $G_f(i\omega)\equiv\sum_{\boldsymbol{k}}G_f(\boldsymbol{k},i\omega)$ being the local Green's function of the f electrons.

The regular and the anomalous Green's functions of f field can be obtained via Eq. (S13) as

$$\begin{pmatrix}G_f(\boldsymbol{p},i\omega)\\ F_f(\boldsymbol{p},i\omega)\end{pmatrix}=\frac{1}{\Gamma(i\omega)+4\Delta^2\xi_{\boldsymbol{p}}^2}\begin{pmatrix}\gamma(-i\omega)\\ -2\Delta\xi_{\boldsymbol{p}}\end{pmatrix},\tag{S19}$$

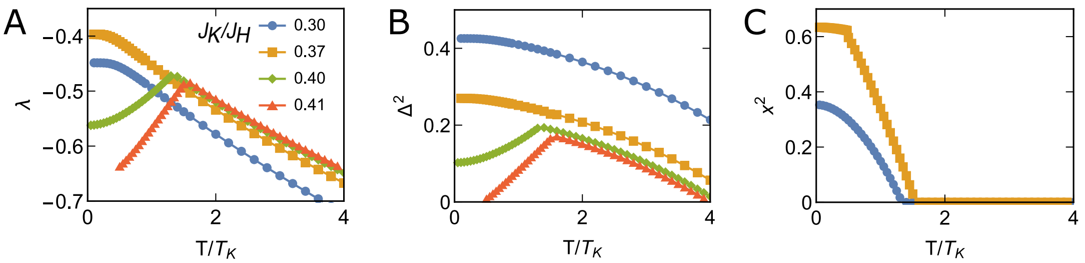

Fig. S1. Mean-field variables as a function of temperature. Temperature dependence of (*A*) λ, (*B*) Δ^2, and (*C*) x^2 with different values of $g \equiv J_K/J_H$ of the channel-asymmetric Kondo lattice model. Here, we choose $J_K' = 2J_K$ and $J_K/D = 1$ fixed.

where $\gamma(i\omega) \equiv i\omega + \lambda - x^2 G_{c0}(i\omega) - \Sigma_f(i\omega)$ and $\Gamma(i\omega) \equiv \gamma(i\omega)\gamma(-i\omega)$. Here, Σ_f is expressed as

$$\Sigma_f(i\omega) = -\frac{K-1}{N}\sum_{\boldsymbol{k},\nu} G_\chi(\boldsymbol{k}, i\nu)G_{c0}(i\omega - i\nu) \\ = -\kappa \sum_{\nu} G_\chi(i\nu)G_{c0}(i\omega - i\nu), \qquad \text{[S20]}$$

which is also momentum-independent.

Momentum and frequency sum. We next perform the momentum sum of G_f analytically and obtain

$$G_f(\omega) = \frac{2}{\pi\gamma(\omega)} E_K\left[-\frac{16\Delta^2}{\gamma(\omega)\gamma^*(-\omega)}\right], \qquad \text{[S21]}$$

where $G_f(\omega)$ here is a function of *real* frequency and $E_K(z)$ is one type of the elliptic integrals, defined as $E_K[z] = \int_{x=0}^{\pi/2} dx(1 - z\sin^2 x)^{-1/2}$. Note that the analytic continuation of $\gamma(i\omega)$, i.e. $\gamma(i\omega) \to \gamma(\omega + i0^+) = \gamma^*(\omega - i0^+)$, has been used in Eq. (S21). The following identity has been also applied in performing the momentum sum of G_f:

$$I_1(A) \equiv \sum_{\boldsymbol{p}\in\Omega} \frac{1}{(\cos p_x + \cos p_y)^2 + A} = \frac{2}{\pi A} E_K\left[-\frac{4}{A}\right], \qquad \text{[S22]}$$

where Ω denotes the first Brillouin zone here. The Matsubara (frequency) sum of Σ_f and Σ_χ can be evaluated using the following identity

$$\frac{1}{\beta}\sum_{\omega} f(i\omega) = \sum_{poles \in f(z)} \frac{\eta}{e^{\beta z} + \eta} \mathrm{Res}\,[f(z)] \qquad \text{[S23]}$$

with $\eta = \pm 1$ for fermion/boson. We obtain

$$\Sigma_f(\omega) = -\kappa \int \frac{dx}{\pi}\left[\frac{1}{e^{\beta x} - 1} G_{c0}(\omega - x)G''_\chi(x) + \frac{1}{e^{-\beta x} + 1} G_\chi(\omega - x)G''_{c0}(x)\right], \\ \Sigma_\chi(\nu) = -\int \frac{dx}{\pi}\frac{1}{e^{\beta x} + 1}\left[G_f(\nu + x)G''_{c0}(x) + G^*_{c0}(x - \nu)G''_f(x)\right], \qquad \text{[S24]}$$

where G'' denotes the imaginary part of the Green's function. Note that, in Eq. (S24), the arguments of the Green's functions and self energies all contain an infinitesimal imaginary part, $i0^+$, due to analytic continuation.

The constraints. The minimization equations (S11) lead to three constraints:

$$\sum_{\boldsymbol{p},\omega} G_f(\boldsymbol{p}, i\omega) = \kappa, \quad \sum_{\boldsymbol{p},\omega} F_f(\boldsymbol{p}, i\omega)\xi_{\boldsymbol{p}} = -\frac{2\Delta}{J_H}, \\ x\sum_{\boldsymbol{p},\omega} G_{c0}(i\omega)G_f(\boldsymbol{p}, i\omega) = -\frac{x}{J_K'}. \qquad \text{[S25]}$$

The summation of F_f over momentum in the above equation can be analytically carried out, with the result

$$\sum_{\boldsymbol{p}} F_f(\boldsymbol{p}, i\omega)\xi_{\boldsymbol{p}} = \frac{1}{-2\Delta}\left\{1 - \frac{2}{\pi}E_K\left[-\frac{16\Delta^2}{\gamma(i\omega)\gamma(-i\omega)}\right]\right\}. \qquad \text{[S26]}$$

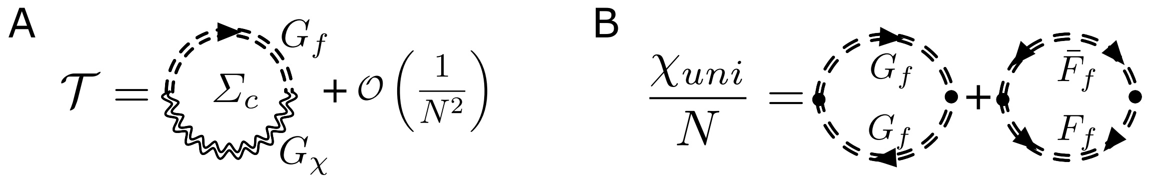

Fig. S2. Feynman diagrams. (*A*), (*B*) The Feynman diagrams of the T-matrix and the uniform spin susceptibility. The solid, dashed, and wavy lines denote the Green's function of the conduction electron, the local electron, and the χ field.

After performing the Matsubara sum, Eq. (S25) can be rewritten as integrals over real frequency:

$$\begin{aligned} \pi\kappa &= -\int_\omega n_F(\omega) G_f''(\omega), \\ \frac{1}{J_H} &= \frac{1}{2\pi^2\Delta^2}\int_\omega n_F(\omega) E_K''\left[\frac{-16\Delta^2}{\gamma(\omega)\gamma^*(-\omega)}\right], \\ \frac{\pi}{J_K'} &= \int_\omega n_F(\omega)\mathrm{Im}\Big[G_f(\omega)G_{c0}(\omega)\Big], \end{aligned} \qquad \text{[S27]}$$

where $n_F(x) = (e^{\beta x}+1)^{-1}$ denotes the Fermi-Dirac distribution with $\beta = 1/T$ ($k_B = 1$ is used). Fig. S1 shows the self-consistent solutions of Δ^2 and x^2 v.s. temperature for different values of $g \equiv J_K/J_H$ with $J_K' = 2J_K$.

Note that multiple solutions in general can be found from Eq. (S25). For example, $\Delta = 0$ is always a solution of the second equation of Eq. (S25), and $x = 0$ is always a solution of the third equation of Eq. (S25). Therefore, in the case of $\Delta = x = 0$, one simply solve the self-consistent equations with the first constraint of Eq. (S27). Similarly, for the case of $\Delta = 0$, $x \neq 0$ and $\Delta \neq 0$, $x = 0$, one only has to solve two of the three constraints. Only in the case of $\Delta \neq 0$, $x \neq 0$, one needs to solve all the three constraints at the same time.

S.III. Scattering T-matrix

The c-electron scattering T-matrix is defined as

$$\mathcal{T}(\omega) = \frac{\Sigma_c(\omega)}{1 - G_{c0}(\omega)\Sigma_c(\omega)}, \qquad \text{[S28]}$$

where Σ_c is the self energy of the conduction electrons,

$$\Sigma_c(i\omega) = -\frac{1}{N}\sum_\nu G_\chi(i\nu)G_f(i\omega + i\nu), \qquad \text{[S29]}$$

which is of order of $O(1/N)$. Therefore, in the large-N limit, T-matrix reduces to $\Sigma_c(\omega)$, which is diagrammatically represented by Fig. S2(*A*). Using Eq. (S23), we can analytically perform the frequency sum,

$$\Sigma_c(\omega) = -\frac{1}{N}\int \frac{dx}{\pi}\left[\frac{1}{e^{\beta x}-1}G_f(x+\omega)G_\chi''(x) - \frac{1}{e^{\beta x}+1}G_\chi^*(x-\omega)G_f''(x)\right]. \qquad \text{[S30]}$$

The imaginary part of the T-matrix in the static limit, $-N\mathcal{T}''(\omega = 0, T)$ corresponds to the scattering rate $\tau^{-1}(\omega = 0, T) = -N\mathcal{T}''(\omega = 0, T)$.

S.IV. Particle-hole symmetry and gapless spectrum

In this section, we examine how the KH Hamiltonian behaves under the particle-hole (PH) transformation, and show that it has PH symmetry at $\kappa = 1/2$. We will further prove that the f and $\hat{\chi}$ fields become gapless when the particle-hole symmetry is present.

We start from the unfactorized Hamiltonian of the Kondo-Heisenberg lattice, given by

$$H = \sum_{i\boldsymbol{P}a\alpha}\varepsilon_{\boldsymbol{P}}\psi_{ia\alpha}^\dagger(\boldsymbol{P})\psi_{ia\alpha}(\boldsymbol{P}) - \sum_{ia\alpha\beta}\frac{J_K^a}{N}\psi_{ia\alpha}^\dagger f_{i\alpha}f_{i\beta}^\dagger\psi_{ia\beta} - \frac{J_H}{N}\sum_{\langle i,j\rangle,\alpha\beta}\left(\tilde{\alpha}f_{i\alpha}^\dagger f_{j,-\alpha}^\dagger\right)\left(\tilde{\beta}f_{j,-\beta}f_{i\beta}\right) + \lambda\sum_{i\alpha}(f_{i\alpha}^\dagger f_{i\alpha} - \kappa), \qquad \text{[S31]}$$

where $\varepsilon_{\boldsymbol{P}} = -2t(\cos P_x + \cos P_y) - \mu$ is the dispersion relation of the conduction electron within each isolated conduction bath, and μ is its chemical potential. Now we perform the PH transformation, defined as

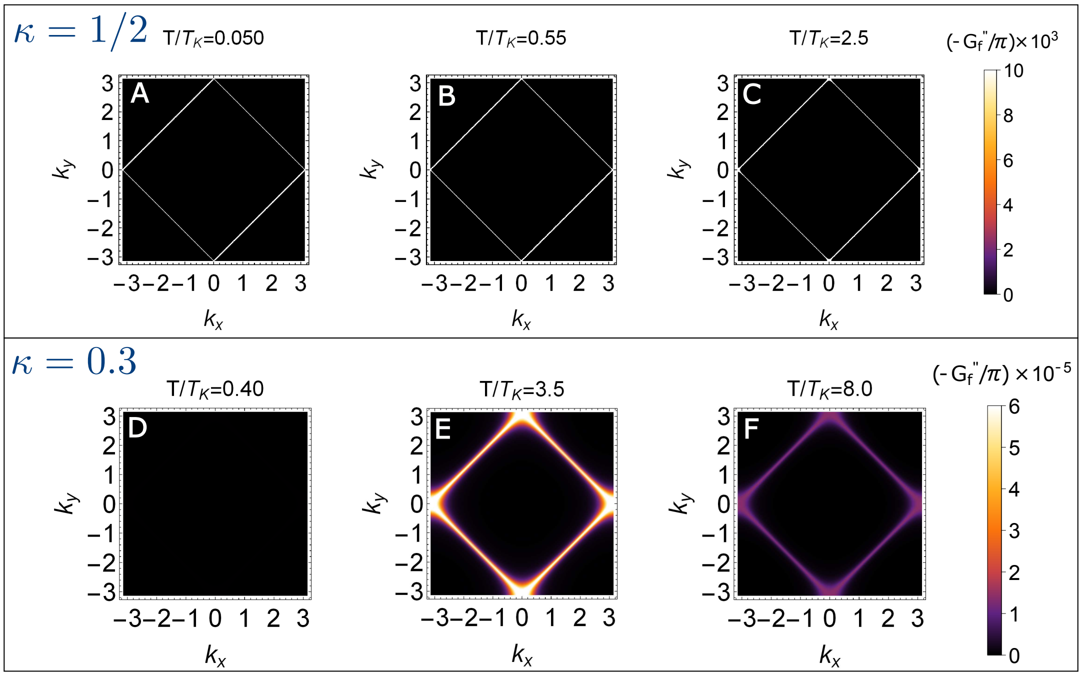

Fig. S3. The spinon Fermi surface at different temperatures for (*A*)-(*C*) $\kappa = 1/2$ and (*D*)-(*F*) $\kappa = 0.3$.

$$\begin{aligned} \psi_{ia\alpha}(\boldsymbol{P}) &\to \psi^\dagger_{ia\alpha}(\tilde{\boldsymbol{P}}), \quad f_{i\alpha} \to f^\dagger_{i\alpha}, \\ \psi^\dagger_{ia\alpha}(\boldsymbol{P}) &\to \psi_{ia\alpha}(\tilde{\boldsymbol{P}}), \quad f^\dagger_{i\alpha} \to f_{i\alpha}, \end{aligned} \tag{S32}$$

where $\tilde{\boldsymbol{P}}$ is the momentum satisfying the relation $\varepsilon_{\boldsymbol{P}} = -\varepsilon_{\tilde{\boldsymbol{P}}}$. For $\mu = 0$, every momentum $\boldsymbol{P}$ in the Brillouin Zone has its PH correspondence $\tilde{\boldsymbol{P}}$. Then we have

$$H_0 \xrightarrow{P.H.} \sum_{i\tilde{\boldsymbol{P}}a\alpha} (-\varepsilon_{\tilde{\boldsymbol{P}}})\psi_{ia\alpha}(\tilde{\boldsymbol{P}})\psi^\dagger_{ia\alpha}(\tilde{\boldsymbol{P}}) = \sum_{i\tilde{\boldsymbol{P}}a\alpha} \varepsilon_{\tilde{\boldsymbol{P}}} \left(\psi^\dagger_{ia\alpha}(\tilde{\boldsymbol{P}})\psi_{ia\alpha}(\tilde{\boldsymbol{P}}) - 1\right) = H_0, \tag{S33}$$

where $\sum_{\tilde{\boldsymbol{P}}} \varepsilon_{\tilde{\boldsymbol{P}}} = 0$ in the case of $\mu = 0$. The Kondo term transforms as

$$\begin{aligned} H_K \xrightarrow{P.H.} &- \sum_{ia\alpha\beta} \frac{J_K^a}{N} \psi_{ia\alpha} f^\dagger_{i\alpha} f_{i\beta} \psi^\dagger_{ia\beta} = - \sum_{ia\alpha\beta} \frac{J_K^a}{N} \psi^\dagger_{ia\beta} f_{i\beta} f^\dagger_{i\alpha} \psi_{ia\alpha} + \frac{J_K^a}{N} \sum_{ia\alpha} \psi^\dagger_{ia\alpha}\psi_{ia\alpha} - \frac{\sum_a J_K^a}{N} \sum_{i\alpha} f^\dagger_{i\alpha} f_{i\alpha} \\ &= H_K + H_0' - \left(\frac{J_K'}{N} + \kappa J_K\right) \sum_{i\alpha} f^\dagger_{i\alpha} f_{i\alpha}, \end{aligned} \tag{S34}$$

where the term $H_0' = \frac{J_K^a}{N} \sum_{ia\alpha} \psi^\dagger_{ia\alpha}\psi_{ia\alpha}$ is sub-leading in the large-N limit as compared to H_0, thus can be dropped. The factor J_K'/N of the third term is negligible as compared with κJ_K. The Heisenberg term transforms as

$$H_J \xrightarrow{P.H.} -\frac{J_H}{N} \sum_{\langle i,j\rangle,\alpha\beta} \left(\tilde{\alpha} f_{i\alpha} f_{j,-\alpha}\right) \left(\tilde{\beta} f^\dagger_{j,-\beta} f^\dagger_{i\beta}\right) = -\frac{J_H}{N} \sum_{\langle i,j\rangle,\alpha\beta} \left(\tilde{\beta} f^\dagger_{i\beta} f^\dagger_{j,-\beta}\right) \left(\tilde{\alpha} f_{j,-\alpha} f_{i\alpha}\right) = H_J. \tag{S35}$$

Finally, the Lagrange multiplier term transforms as

$$\lambda \sum_{i\alpha} (f^\dagger_{i\alpha} f_{i\alpha} - \kappa) \xrightarrow{P.H.} \lambda \sum_{i\alpha} (f_{i\alpha} f^\dagger_{i\alpha} - \kappa) = \lambda \sum_{i\alpha} (1 - \kappa - f^\dagger_{i\alpha} f_{i\alpha}). \tag{S36}$$

Combining Eq. (S33)-(S36), we have

$$H = \tilde{H} + \lambda \sum_{i\alpha} f^\dagger_{i\alpha} f_{i\alpha} - \lambda N_s N \kappa \xrightarrow{P.H.} \tilde{H} - (\lambda + \kappa J_K) \sum_{i\alpha} f^\dagger_{i\alpha} f_{i\alpha} + \lambda N_s N (1 - \kappa), \tag{S37}$$

where $\tilde{H} = H_0 + H_K + H_J$. Note that the constraint also transforms under PH transformation:

$$\left\langle f_{i\alpha}^\dagger f_{i\alpha} \right\rangle = \kappa \xrightarrow{P.H.} \left\langle f_{i\alpha}^\dagger f_{i\alpha} \right\rangle = 1 - \kappa. \quad [S38]$$

Therefore,the Hamiltonian is invariant (up to a constant) under the PH transformation only at $\kappa = 1/2$, with the condition

$$\lambda = -\frac{\kappa J_K}{2}. \quad [S39]$$

Our numerical calculations indeed find $\lambda = -J_K/4$ for $\kappa = 1/2$.

An alternative approach to the derivation of particle-hole symmetry of our model. Here, we present an alternative approach to the derivation of particle-hole symmetry of our model based on the Hubbard-Stratonovich factorized form of the Kondo-Heisenberg Hamiltonian, given by

$$H = \sum_{i\boldsymbol{P}\alpha a} \varepsilon_{\boldsymbol{P}} \psi_{i\alpha a}^\dagger(\boldsymbol{P}) \psi_{i\alpha a}(\boldsymbol{P}) - \lambda \sum_{i\alpha} f_{i\alpha}^\dagger f_{i\alpha} + N N_s \kappa \lambda + \frac{1}{\sqrt{N}} \sum_{ia\alpha} \left(\chi_{ia}^\dagger f_{i\alpha}^\dagger \psi_{ia\alpha} + \psi_{ia\alpha}^\dagger f_{i\alpha} \chi_{ia} \right) + \sum_{i,a} \frac{|\chi_{ia}|^2}{J_K^a}$$
$$+ \sum_{\langle i,j \rangle} \left(\Delta_{ij} \sum_\alpha \tilde{\alpha} f_{j,-\alpha} f_{i\alpha} + H.c. + \frac{N|\Delta_{ij}|^2}{J_H} \right). \quad [S40]$$

The PH transformation now involves in the two auxiliary bosonic fields,

$$\left[f_{i\alpha}^\dagger,\, \psi_{i\alpha a}^\dagger(\boldsymbol{P}),\, \chi_{ia}^\dagger,\, \Delta_{ij}^\dagger \right] \xrightarrow{P.H.} \left[f_{i\alpha},\, \psi_{i\alpha a}(\tilde{\boldsymbol{P}}),\, -\chi_{ia},\, -\Delta_{ji} \right], \quad [S41]$$

where $\tilde{\boldsymbol{P}}$ is the momentum satisfying the relation $\varepsilon_{\boldsymbol{P}} = -\varepsilon_{\tilde{\boldsymbol{P}}}$.

Note that, under particle-hole transformation, the Kondo hybridization field χ_{ia} and the RVB spin-singlet field Δ_{ij} pick a minus sign. This result can be easily understood if we evaluate particle-hole transformation of their mean-field values. Let us start from the mean-field value of χ_{ia},

$$\langle \chi_{ia} \rangle = -\frac{1}{\sqrt{N}} \sum_\alpha J_K^a \langle f_{i\alpha}^\dagger \psi_{ia\alpha} \rangle, \quad \langle \chi_{ia}^\dagger \rangle = -\frac{1}{\sqrt{N}} \sum_\alpha J_K^a \langle \psi_{ia\alpha}^\dagger f_{i\alpha} \rangle. \quad [S42]$$

Under PH transformation, we have

$$-\frac{1}{\sqrt{N}} \sum_\alpha J_K^a \langle f_{i\alpha}^\dagger \psi_{ia\alpha} \rangle \xrightarrow{P.H.} -\frac{1}{\sqrt{N}} \sum_\alpha J_K^a \langle f_{i\alpha} \psi_{ia\alpha}^\dagger \rangle = -\langle \chi_{ia}^\dagger \rangle. \quad [S43]$$

We thus conclude $\chi_{ia} \xrightarrow{P.H.} -\chi_{ia}^\dagger$ for every channels. Following a similar approach, we can also obtain $\Delta_{ij} \xrightarrow{P.H.} -\Delta_{ji}^\dagger$.

The PH transformation on H_0 and the local constraint term has been shown above. Below, we only evaluate the PH transformation on H_K and H_J.

Upon performing the PH transformation on the Kondo term, we have

$$\frac{1}{\sqrt{N}} \sum_{ia\alpha} \left(\chi_{ia}^\dagger f_{i\alpha}^\dagger \psi_{ia\alpha} + \psi_{ia\alpha}^\dagger f_{i\alpha} \chi_{ia} \right) + \sum_{ia} \frac{|\chi_{ia}|^2}{J_K} \xrightarrow{P.H.} -\frac{1}{\sqrt{N}} \sum_{ia\alpha} \left(\chi_{ia} f_{i\alpha} \psi_{ia\alpha}^\dagger + \psi_{ia\alpha} f_{i\alpha}^\dagger \chi_{ia}^\dagger \right) + \sum_{ia} \frac{\chi_{ia} \chi_{ia}^\dagger}{J_K}. \quad [S44]$$

In Eq. (S44), we need to know the commutation relation of the Kondo hybridization boson field χ at the Hamiltonian level to deal with e.g. the $\chi_{ia}\chi_{ia}^\dagger$ term. However, there is no well-defined commutation relation for the χ field as it is a "boson field" (a c-number), originally introduced within the path-integral formalism, instead of a canonical boson operator. We first need to promote the χ_{ia} onto a second quantized operator. Here, we take

$$\chi_{ia} = -\frac{J_K^a}{\sqrt{N}} \sum_\alpha f_{i\alpha}^\dagger \psi_{ia\alpha}, \qquad \chi_{ia}^\dagger = -\frac{J_K^a}{\sqrt{N}} \sum_\alpha \psi_{ia\alpha}^\dagger f_{i\alpha}. \quad [S45]$$

The above equation satisfies Eq. (S41) to Eq. (S43). Let $J_K^{a<K} = J_K$ and $J_K^{a=K} = J_K'$, the quadratic-term-in-χ in Eq. (S44) becomes

$$\sum_{ia} \frac{\chi_{ia}\chi_{ia}^\dagger}{J_K} = \sum_{ia} \frac{\chi_{ia}^\dagger \chi_{ia}}{J_K} + \left(\frac{K-1}{N} J_K + \frac{J_K'}{N} \right) \sum_{i\alpha} f_{i\alpha}^\dagger f_{i\alpha}, \quad [S46]$$

while the three-field term of Eq. (S44) after the PH transformation is

$$\frac{1}{\sqrt{N}}\sum_{ia\alpha}\left(\chi_{ia}^{\dagger}f_{i\alpha}^{\dagger}\psi_{ia\alpha}+\psi_{ia\alpha}^{\dagger}f_{i\alpha}\chi_{ia}\right)-2\left(\frac{K-1}{N}J_K+\frac{J_K'}{N}\right)\sum_{i\alpha}f_{i\alpha}^{\dagger}f_{i\alpha}. \tag{S47}$$

Following a similar approach, the Heisenberg term can be shown to be invariant under PH transformation

$$\begin{aligned}\sum_{\langle i,j\rangle,\alpha}\left(\Delta_{ij}\tilde{\alpha}f_{j,-\alpha}f_{i\alpha}+H.c.+\frac{N|\Delta_{ij}|^2}{J_H}\right)&\xrightarrow{P.H.}-\sum_{\langle i,j\rangle,\alpha}\left(\Delta_{ji}^{\dagger}\tilde{\alpha}f_{j,-\alpha}^{\dagger}f_{i\alpha}^{\dagger}+H.c.+\frac{N\Delta_{ji}\Delta_{ji}^{\dagger}}{J_H}\right)\\&=\sum_{\langle i,j\rangle,\alpha}\left(\Delta_{ij}^{\dagger}\tilde{\alpha}f_{i\alpha}^{\dagger}f_{j,-\alpha}^{\dagger}+H.c.+\frac{N|\Delta_{ij}|^2}{J_H}\right).\end{aligned} \tag{S48}$$

Taking the large-N, K limit and combining Eqs. (S41)-(S48), the total Hamiltonian after the PH transformation reads

$$H\xrightarrow{P.H.}H_0+H_K+H_J-(\lambda+\kappa J_K)\sum_{i\alpha}f_{i\alpha}^{\dagger}f_{i\alpha}+\lambda N_sN(1-\kappa), \tag{S49}$$

identical to that of the previous approach. Comparing the non-interacting term of the f spinon, the PH symmetry can only occur if $-(\lambda+\kappa J_K)=\lambda$, indicating

$$\lambda=-\frac{\kappa J_K}{2}, \tag{S50}$$

consistent with the previous result. Note that there is a constant term $\lambda N_sN(1-\kappa)$ which is different from that of the original Hamiltonian. This constant term affects the local constraint and we will show that this imposes another condition for the occurrence of PH symmetry for H: the constraint before and after the PH transformation can be shown as

$$\left\langle f_{i\alpha}^{\dagger}f_{i\alpha}\right\rangle=\kappa\xrightarrow{P.H.}\left\langle f_{i\alpha}^{\dagger}f_{i\alpha}\right\rangle=1-\kappa. \tag{S51}$$

The PH symmetry can only occur if the constraint after PH transformation remains unchanged, that is $\kappa=1/2$.

To conclude, we have provided another approach to show the PH transformation of the Kondo-Heisenberg lattice model. Based on the above derivation, PH symmetry connects $H(\kappa)$ and $H(1-\kappa)$ via $H(\kappa)\xrightarrow{P.H.}H(1-\kappa)$. This implies that the particle excitations of $H(\kappa)$ are equal to the hole excitations of $H(1-\kappa)$. In particular, for $\kappa=1/2$, the particle excitation of $H(\kappa=1/2)$ is equal to its hole excitations due to $H(1/2)\xrightarrow{P.H.}H(1/2)$. This is manifested by the spectral function of the f field, that is $-G_f''(\omega)/\pi=-G_f''(-\omega)/\pi$, see Fig. 1($C$) of the main text. In this section, we examine how the KH Hamiltonian of the ψ and f-electron sectors behaves under the particle-hole (PH) transformation with different values of κ. We will also show that the f and $\hat{\chi}$ fields become gapless when the particle-hole symmetry is preserved for $\kappa=1/2$.

The gapless spectrum. Next, we prove that the spectral functions of the f and χ fields are gapless at $\omega,T\to0$ when the particle-hole symmetry is present ($\kappa=1/2$). Note that the following derivations for the gapless spectral functions require only the particle-hole symmetry of the spinon (f) sector but they do not require the PH symmetry of conduction band. Using the fact that $\Sigma_f''(\omega=0)=0$ at zero temperature, the Green's function of f fields at low frequencies can be approximated as

$$G_f(\boldsymbol{p},\omega)=\frac{\omega-\lambda+\Sigma_f'(0)+i\delta}{[\omega+\lambda-\Sigma_f'(0)+i\delta][\omega-\lambda+\Sigma_f'(0)+i\delta]-4\Delta^2\xi_{\boldsymbol{p}}^2},\quad\text{for }\omega\approx0, \tag{S52}$$

where $\Sigma_f'(0)$ is the real part of f-spinon self-energy at $\omega=0$. The dispersion of f-spinons is determined by the pole of $G_f(\boldsymbol{p},\omega)$ shown above, leading to

$$\omega=\pm\sqrt{(\lambda-\Sigma_f'(0))^2+4\Delta^2\xi_{\boldsymbol{p}}^2}, \tag{S53}$$

which becomes gapless if $\lambda=\Sigma_f'(0)$. According to Eq. (S24), we have

$$\Sigma_f'(0)=-\kappa\int\frac{dx}{\pi}\left[\frac{1}{e^{\beta x}-1}G_{c0}'(-x)G_\chi''(x)+\frac{1}{e^{-\beta x}+1}G_\chi'(-x)G_{c0}''(x)\right]. \tag{S54}$$

A simple demonstration of $\Sigma_f'(0)=\lambda=-\kappa J_K/2$ in the presence of PH symmetry can be achieved by a replacement of the full Green's function $G_\chi(x)$ with its bare form $G_{\chi,0}(x)=-J_K$ in the above equation, which leads to

$$\Sigma_f'(0)=\kappa J_K\int\frac{dx}{\pi}\frac{1}{e^{-\beta x}+1}G_{c0}''(x)=-\frac{\kappa J_K}{2}. \tag{S55}$$

Using the full Green's function $G_\chi(x)$, our numerical calculations found that the above identity is always preserved at $\kappa = 1/2$, which gives rise to a gapless spectral function of f.

Below, we provide another equivalent approach to demonstrate that $\Xi = \lambda - \Sigma'_f(0) = 0$ for $\kappa = 1/2$. Note that the following derivations for the gapless spectral functions require only the particle-hole symmetry of the spinon (f) sector but they do not require the PH symmetry of conduction band. Since $H(\kappa = 1/2) \xrightarrow{P.H.} H(\kappa = 1/2)$, we analytically show below that this indicates that $\Xi = 0$, which perfectly agrees with what we find by numerically solving the self-consistent equations. To derive this result, we perform PH transformation on the effective Hamiltonian for the f-electron in the strange metal phase, $\tilde{H}_f^{SM}$, after integrating out the $\hat{\chi}$ and ψ fields. The effective Hamiltonian $\tilde{H}_f^{SM}$ reads

$$\tilde{H}_f^{SM} = \sum_{\boldsymbol{p}\alpha} \Xi f^\dagger_{\boldsymbol{p}\alpha} f_{\boldsymbol{p}\alpha} + \sum_{\boldsymbol{p}\alpha} [\tilde{\alpha}\Delta_{\boldsymbol{p}} f_{\boldsymbol{p}\alpha} f_{-\boldsymbol{p},-\alpha} + H.c.] = \sum_{\boldsymbol{p}\alpha} \varepsilon_\gamma(\boldsymbol{p}) \gamma^\dagger_{\boldsymbol{p}\alpha}\gamma_{\boldsymbol{p}\alpha} \quad \text{[S56]}$$

with $\gamma_{\boldsymbol{p}\alpha} = u^*_{\boldsymbol{p}} f_{\boldsymbol{p}\alpha} + v_{\boldsymbol{p}} f^\dagger_{-\boldsymbol{p},-\alpha}$ being the Bogoluibov quasiparticle of spinons and $\varepsilon_\gamma(\boldsymbol{p}) = \sqrt{\Xi^2 + \Delta^2_{\boldsymbol{p}}}$ being the quasiparticle dispersion. Here, u and v above are the coherent factors, given by

$$|u_{\boldsymbol{p}}|^2 = \frac{1}{2}\left(1 + \frac{\Xi}{\varepsilon_\gamma(\boldsymbol{p})}\right), \quad |v_{\boldsymbol{p}}|^2 = \frac{1}{2}\left(1 - \frac{\Xi}{\varepsilon_\gamma(\boldsymbol{p})}\right). \quad \text{[S57]}$$

Upon acting particle-hole transformation, $\tilde{H}_f^{SM}$ becomes

$$\tilde{H}_f^{SM} \xrightarrow{P.H.} \sum_{\boldsymbol{p}\alpha} \Xi f_{-\boldsymbol{p}\alpha} f^\dagger_{-\boldsymbol{p}\alpha} - \sum_{\boldsymbol{p}\alpha} [\tilde{\alpha}\Delta_{\boldsymbol{p}} f_{\boldsymbol{p}\alpha} f_{-\boldsymbol{p},-\alpha} + H.c.] = \sum_{\boldsymbol{p}\alpha} \Xi + \tilde{H}_f^{SM}. \quad \text{[S58]}$$

Since the whole H is invariant after PH transformation for $\kappa = 1/2$, we also require $\tilde{H}_f^{SM} \xrightarrow{P.H.} \tilde{H}_f^{SM}$. As a result, we have $\Xi = 0$, see Eq. (S58). Note that in this case, we have $u_{\boldsymbol{p}} = v_{\boldsymbol{p}} = 1/\sqrt{2}$.

As a consequence, we find that the spectral function of f-spinons at $\omega = 0$ displays a Van-Hove singularity,

$$-\frac{1}{\pi} G''_f(\omega = 0) = \frac{1}{2\pi^2 \Delta} \int_0^{\pi/2} dx \csc x \to \infty. \quad \text{[S59]}$$

In the RVB phase where the holon field χ is not Bose-condensed, we can further prove that $-G''_\chi(\omega = 0) = 0$ by applying Lehmann representation for the spectral function of the bosonic χ field:

$$-\frac{1}{\pi} G''_\chi(\boldsymbol{k}, \omega) = \sum_\nu |\langle \nu; \mathcal{N}+1|\chi^\dagger_{\boldsymbol{k}}|0; \mathcal{N}\rangle|^2 \delta(\omega - \omega_\nu^{\mathcal{N}+1}) - \sum_\nu |\langle \nu; N-1|\chi_{\boldsymbol{k}}|0; \mathcal{N}\rangle|^2 \delta(\omega + \omega_\nu^{\mathcal{N}-1}), \quad \text{[S60]}$$

where $\omega_\nu^{\mathcal{N}\pm 1}$ represents the excitation energy between the state $|\nu; \mathcal{N}\pm 1\rangle$ (labeled by ν) with particle number $\mathcal{N}\pm 1$ and energy level $E_\nu^{\mathcal{N}\pm 1}$ and the ground state $|0; \mathcal{N}\pm 1\rangle$ with particle number $\mathcal{N}\pm 1$ and energy level $E_0^{\mathcal{N}\pm 1}$, namely $\omega_\nu^{\mathcal{N}\pm 1} \equiv E_\nu^{\mathcal{N}\pm 1} - E_0^{\mathcal{N}\pm 1} \geq 0$. If the χ field does not get Bose-condensed, there is no gapless Goldstone excitation, thus $\omega_\nu^{\mathcal{N}\pm 1} \neq 0$. It is then straightforward to show that $-G''_\chi(\boldsymbol{k}, \omega = 0) = 0$ via Eq. (S60):

$$-\frac{1}{\pi} G''_\chi(\boldsymbol{k}, \omega = 0) = \sum_\nu |\langle \nu; \mathcal{N}+1|\chi^\dagger_{\boldsymbol{k}}|0; \mathcal{N}\rangle|^2 \times \delta(-\omega_\nu^{\mathcal{N}+1}) - \sum_\nu |\langle \nu; N-1|\chi_{\boldsymbol{k}}|0; \mathcal{N}\rangle|^2 \times \delta(\omega_\nu^{\mathcal{N}-1}) = 0 \quad \text{[S61]}$$

as the δ functions are equal to zero. Furthermore, for $\omega \neq 0$, we will prove that $-G''_\chi(\omega \neq 0) \neq 0$ as follows: First, $\Sigma''_\chi(\omega \neq 0)$ can be proved to be nonzero if $G_f(\omega)$ is gapless, via Eq. (S24) of Section S.II:

$$-\Sigma''_\chi(\omega) = \rho_0 \int_{-D}^{D} dx \, [n_f(x+\omega) - n_f(x)] \, G''_f(\omega + x) \neq 0, \quad \text{[S62]}$$

where we have used the following result:

$$\begin{aligned} G''_{c0}(\omega) =& \rho_0 \left[\tan^{-1}\left(\frac{\omega - D}{\delta}\right) - \tan^{-1}\left(\frac{\omega + D}{\delta}\right)\right] \\ =& \begin{cases} -\pi\rho_0, & -D < \omega < D \\ 0, & \text{other} \end{cases} \end{aligned} \quad \text{[S63]}$$

with D being the half-bandwidth of conduction electron and $\rho_0 \sim D^{-1}$ being the density of states of conduction electrons at the Fermi energy. Eq. (S62) also indicates that $-G''_\chi(\omega \neq 0)$ is nonzero via

$$-G''_\chi(\omega) = \frac{-\Sigma''_\chi(\omega)}{\left[J_K^{-1} + \Sigma'_\chi(\omega)\right]^2 + \Sigma''^{\,2}_\chi(\omega)}. \quad \text{[S64]}$$

Based on Eqs. (S61)-(S64), we conclude that the χ field is gapless (semi-metallic) at $\kappa = 1/2$.

The above derivation and proof offer an analytical understanding of our self-consistent solutions obtained numerically on the gapless spinon and holon spectral functions for $\kappa = 1/2$. Though our calculations of a gapless fermionic spin liquid phase are based on the 2D square lattice, we believe that this mechanism is a generic feature of our approach, not restricted to the square lattice. It is applicable for other 2D lattices with or without geometrical frustration.

S.V. Stability of the RVB phases against U(1) gauge fluctuations and the spinon Fermi surface

In this section, we study the stability of our RVB phases we found in our phase diagrams against the U(1) gauge-field fluctuations.

In Ref. (10), the authors demonstrated via renormalization group (RG) analysis that, in a 2D spin-liquid state with gapless Dirac spinons and infinitely many spin flavors, the spinon excitations are not confined by the U(1) gauge force. In this case, the spin-liquid state with discrete points on the Fermi surface can be thus a stable ground state in the large-N limit. Subsequently, the author of Ref. (11) generalized the work of Ref. (10) to the case that the gapless spinon modes form an extended Fermi surface with infinitely many spinon modes on it, and studied the stability of the gapless spin-liquid state against the U(1) gauge-field fluctuations. The author of Ref. (11) obtained similar results to Ref. (10): The U(1) gauge-field fluctuations fail to confine the spinons. These results can be further applied to the finite-N case.

Here, to support the stability of the RVB phases against the U(1) gauge-field fluctuations, we provide numerical evidences that both the gapless non-Fermi liquid (NFL) spin liquid phase for $\kappa = 1/2$ and the NFL region in the spin-liquid side for $\kappa \neq 1/2$ in our phase diagram acquire an extended Fermi surface. The spectral functions of Fig. S3 are numerically evaluated from the imaginary part of the spinon Green's function from Eq. (S19), $(-1/\pi)G''_f(\omega = 0, \boldsymbol{k})$, at zero frequency $\omega = 0$ over the first Brillouin zone of a 2D square lattice $\boldsymbol{k} \in$ FBZ. The results of the spinon Fermi surface are illustrated in Fig. S3. Alternatively, the finite spinon Fermi surface at zero temperature for $\kappa = 1/2$, similar to the low-temperature one of Fig. S3(A), can also be analytically evaluated from Eq. (S19): In the spin-liquid side for $\kappa = 1/2$ at zero temperature and zero frequency, the Kondo hybridization field is not condensed, i.e. $x = 0$. The spectral weight of G_f, defined as $-G''_f(\omega = 0, \boldsymbol{k})/\pi$, takes the form

$$-\frac{1}{\pi}G''_f(\omega = 0, \boldsymbol{k}) = \frac{\eta/\pi}{\eta^2 + 4\Delta^2\xi^2_{\boldsymbol{k}}}. \qquad \text{[S65]}$$

Eq. (S65) implies that nonzero spectral weights of spinon excitation for $\eta \to 0$ and $\Delta \neq 0$ occurs at the certain values of quasi-momentum $\boldsymbol{k} = \boldsymbol{k}_0 = (k_{x0},\, k_{y0})$ in the First Brillouin zone at which $\xi_{\boldsymbol{k}_0} = \cos k_{x0} + \cos k_{y0} = 0$. Those quasi-momentums $\boldsymbol{k}_0$ form a spinon Fermi surface at zero temperature and at zero frequency, and its shape is identical to the Fermi surface of the nearest-neighbor tight-binding model at half-filling on a square lattice.

We provide numerical evidences for spinon Fermi surface in Figs. S3(A)-S3(C) that our large-N and finite-N results for the strange metal phase ($\kappa = 1/2$) are qualitatively reliable despite the presence of U(1) gauge fluctuations. For $\kappa \neq 1/2$, we separate the discussions into two parts: At low temperatures, the spinons are gapped. This leads to confinement via the instanton effect of the U(1) gauge field and the true ground state is dominated by the valence bond solid (VBS) state, see Refs. (12–14). At relatively higher temperatures, the spinons are effectively deconfined and, according to the works of Refs. (10, 11), our calculations for the strange metal spin-liquid region [gray area of the phase diagram Fig. 1(A) of the main text] still remain qualitatively unchanged as the spinons in this regime acquire a finite Fermi surface [see Figs. S3(E) and S3(F)], suggesting the U(1) gauge-field fluctuations are not harmful for these phases/regions.

S.VI. Entropy and Specific heat coefficient

The entropy is derived using the following formula from Ref. (15):

$$\frac{S}{N} = -\int \frac{dz}{\pi}\left\{K\frac{dn_F}{dT}\left(\mathrm{Im}\ln\left[-G_c^{-1}\right] + \Sigma''_c G'_c\right) + \frac{dn_F}{dT}\left(\frac{1}{2}\mathrm{Tr}\sum_{\boldsymbol{p}}\mathrm{Im}\ln\left[-\mathbf{G}_f^{-1}(\boldsymbol{p})\right] + \Sigma''_f G'_f\right)\right.$$
$$\left. +\kappa\frac{dn_B}{dT}\left(\mathrm{Im}\ln\left[-G_\chi^{-1}\right] + \Sigma''_\chi G'_\chi\right)\right\}, \qquad \text{[S66]}$$

where z does not explicitly shown in the integrand. The momentum sum of the above equation can be performed as follows:

$$\frac{1}{2}\mathrm{Tr}\sum_{\boldsymbol{p}}\mathrm{Im}\ln\left[-\mathbf{G}_f^{-1}(\boldsymbol{p})\right] = \frac{1}{2\pi}\mathrm{Im}\mathcal{G}^{2,2}_{3,3}\left(\frac{16\Delta^2}{\gamma(z)\gamma^*(-z)}\Big|^{1/2,1/2,1}_{0,0,0}\right), \qquad \text{[S67]}$$

where the identity $\mathrm{Tr}\ln(A) = \ln\det(A)$ is used and $\mathcal{G}$ is the Meijer-G function. If $\Delta = 0$, the second line of Eq. (S66) is simply $\frac{dn_F}{dT}\left(\mathrm{Im}\ln\left[-G_f^{-1}\right] + \Sigma''_f G'_f\right)$. The first line of Eq. (S66) can be simplified as follows:

$$K\frac{dn_F}{dT}\left(\mathrm{Im}\ln\left[-G_c^{-1}\right] + \Sigma''_c G'_c\right) \approx \frac{dn_F}{dT}\left(K\mathrm{Im}\ln\left[-G_{c0}^{-1}\right] - \kappa N\Sigma'_c G''_{c0}\right). \qquad \text{[S68]}$$

Note that $K\mathrm{Im}\ln\left[-G_{c0}^{-1}\right]$ is proportional to N, while $\kappa N\Sigma'_c G''_{c0}$ is of order of unity since $\Sigma'_c \propto \frac{1}{N}$. The former is associated with the electron baths around each impurity site, and will be neglected in our calculations.

In conclusion, one has

$$\frac{S}{N} = -\int\frac{dz}{\pi}\left\{\frac{dn_F}{dT}\left[\Sigma''_f G'_f - \kappa N\Sigma'_c G''_{c0} + \frac{1}{2\pi}\mathrm{Im}\mathcal{G}^{2,2}_{3,3}\left(\frac{16\Delta^2}{\gamma(z)\gamma^*(-z)}\Big|^{1/2,1/2,1}_{0,0,0}\right)\right] + \kappa\frac{dn_B}{dT}\left[\mathrm{Im}\ln(-G_\chi^{-1}) + \Sigma''_\chi G'_\chi\right]\right\} \qquad \text{[S69]}$$

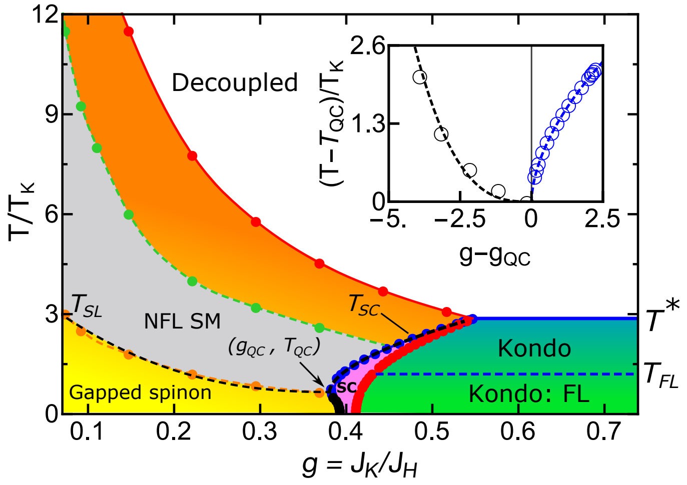

Fig. S4. The finite-temperature phase diagram for the channel-asymmetric ($J'_K = 2J_K$) large-N Kondo lattice model for $\kappa = 0.3$. Different colors correspond to regions with different behaviors of physical quantities. The black dashed lines on both sides of g_{QC} are power-law fits of $|g - g_{QC}|$ for the crossover scales, T_{SL} and T_{SC}, of the NFL strange metal (SM) region (gray). Inset: power-law vanish of the crossover scales, T_{SL} and T_{SC}, with respect to T_{QC}: $T_{SC} - T_{QC} \propto |g - g_{QC}|^{2.3}$ (black dashed line), $T_{SC} - T_{QC} \propto |g - g_{QC}|^{0.6}$ (blue dashed line), associated with the gapped spinon and coexisting superconducting phases, respectively.

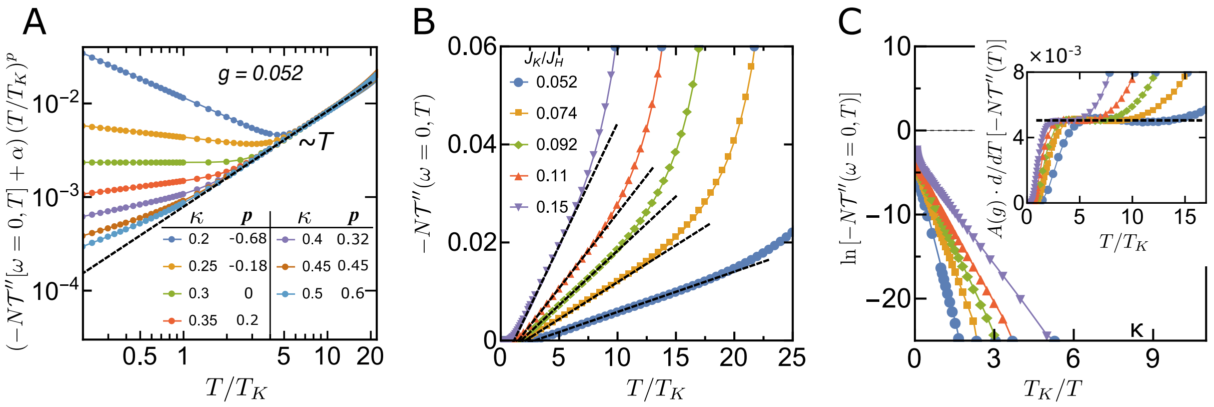

Fig. S5. Scattering T-matrix of the conduction electrons of the gapped VBS state for $\kappa = 0.3$. (*A*) The scaling of static T-matrix for different values κ with $g = 0.052$ fixed. Y is a fitting parameter. (*B*) $-N\mathcal{T}''(\omega = 0,\ T)$ with different values of g at $\kappa = 0.3$. The black dashed lines in (*A*) and (*B*) are linear fits. (*C*) displays the exponential decay of (*B*) at low temperatures. Insets of (*C*): temperature derivative of (*B*) rescaled by a non-universal factor $A(g)$.

for $\varDelta \neq 0$, and

$$\frac{S}{N} = \frac{-1}{\pi} \int_z \left\{ \frac{dn_F}{dT} \left[\mathrm{Im}\ln(-\gamma) + \Sigma''_f G'_f - K \Sigma'_c G''_{c0} \right] + \kappa \frac{dn_B}{dT} \left[\mathrm{Im}\ln(-G_\chi^{-1}) + \Sigma''_\chi G'_\chi \right] \right\} \qquad \text{[S70]}$$

for $\varDelta = 0$, respectively.

S.VII. Magnetic Susceptibility

In the large-N limit, the local moment magnetization along the z direction can be defined as

$$M_z(r_i) = \frac{1}{\beta} \int_{\tau=0}^{\beta} \sum_\sigma \tilde{\sigma} \left\langle f^*_{i\sigma}(\tau) f_{i\sigma}(\tau) \right\rangle , \qquad \text{[S71]}$$

which is zero in the absence of external magnetic field. The susceptibility can be derived by coupling the above magnetization to an external source and taking functional derivative of the magnetization with respect to the source. The susceptibility, in general, takes the following form

$$\frac{\chi(\boldsymbol{p}, \nu)}{N} = -\frac{1}{\beta} \sum_{\boldsymbol{k}\omega} \left[G_f(\boldsymbol{k}, \omega) G_f(\boldsymbol{p} + \boldsymbol{k}, \omega + \nu) \ + F_f(\boldsymbol{k}, \omega) \bar{F}_f(\boldsymbol{p} + \boldsymbol{k}, \omega + \nu) \right] . \qquad \text{[S72]}$$

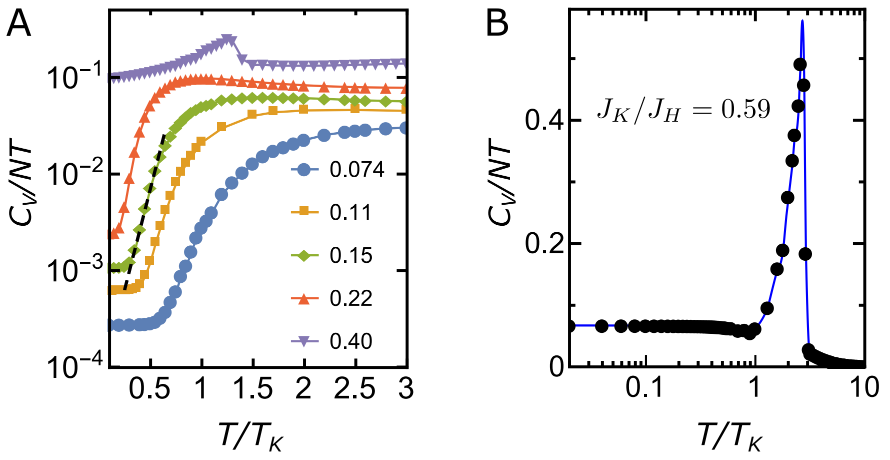

Fig. S6. Specific heat coefficient C_V/NT for $\kappa = 0.3$. (*A*) C_V/NT in the gapped VBS state side and (*B*) in the FL side. In (*A*), C_V/NT shows an exponential decay (fitted by the black dashed line) before it saturates. (*B*) shows that C_V/NT saturates at the low temperature regime, a signature of the FL phase.

The analytical form of Eq. (S72) is diagrammatically represented by Fig. S2(*B*). For $\boldsymbol{p} = 0$ and $\nu = 0$, $\chi(\mathbf{0}, 0) \equiv \chi_{uni}$ represents the *static uniform susceptibility*. For $\boldsymbol{p} = 0$ and $\nu = 0$, the momentum sum of Eq. (S72) is analytically evaluated as

$$\sum_{\boldsymbol{k}} G_f^2(\boldsymbol{k}, \omega) = \frac{\gamma(-\omega)}{\gamma(\omega)} \frac{X}{16\pi\Delta^2} \left[\frac{E_E(-X)}{1+X} + E_K(-X) \right] \qquad \text{[S73]}$$

via $-\partial I_1/\partial A$ [Eq. (S22)], where $X \equiv 16\Delta^2/(\gamma(\omega)\gamma^*(-\omega))$, and

$$\sum_{\boldsymbol{k}} F_f(\boldsymbol{k}, \omega)\bar{F}_f(\boldsymbol{k}, \omega) = \frac{1}{16\pi\Delta^2} \left[\frac{E_K(-X)}{X} - \frac{E_E(-X)}{1+X} \right] \qquad \text{[S74]}$$

via using $-\partial I_2/\partial A$.

For $\boldsymbol{p} = 0$ and $\nu \neq 0$, Eq. (S72) becomes the dynamical uniform susceptibility. In Eq. (S73), $E_E(z) = \int_0^{\pi/2} dx\sqrt{1 - z\sin^2 x}$, which is related to $E_K(z)$ through

$$\frac{\partial}{\partial z} E_K(z) = \frac{E_E(z) - (1-z)E_K(z)}{2z(1-z)}. \qquad \text{[S75]}$$

S.VIII. Correlation functions and observables under gauge transformation

In this section, we present the relevant correlation function and observables under the gauge transformation of Eq. (S4) in Section S.I.

The spinon f and the holon $\hat{\chi}$ field are not gauge invariant under the gauge transformation of Eq. (S4), and neither are their Green's functions $G_f(\boldsymbol{r}_i, \boldsymbol{r}_j; \tau) = -\langle T_\tau f(\boldsymbol{r}_i, \tau) f^\dagger(\boldsymbol{r}_j, 0)\rangle$ and $G_\chi(\boldsymbol{r}_i, \boldsymbol{r}_j; \tau) = -\langle T_\tau \hat{\chi}(\boldsymbol{r}_i, \tau)\hat{\chi}^\dagger(\boldsymbol{r}_j, 0)\rangle$.

However, the thermodynamical observables, spin susceptibility and specific heat coefficient, and the T-matrix we present in this article are all gauge-invariant: the spin susceptibility and specific heat coefficient can be obtained by taking the derivative of the free energy $F = -T\ln Z$ with Z being the partition function and thus are gauge-invariant in that the action $\mathcal{S}$ is invariant under the gauge transformation as we have shown in the previous section. Alternatively, the spin susceptibility can be proved to be gauge-invariant from its definition. In the absence of external field, the general expression of spin susceptibility, χ_{ij}, takes the form:

$$\chi_{ij} = \sum_{\nu'\alpha'} \sum_{\nu\alpha} \alpha\alpha' \left\langle f^\dagger_{i\nu\alpha} f_{i\nu\alpha} f^\dagger_{j\nu'\alpha'} f_{j\nu'\alpha'} \right\rangle_c. \qquad \text{[S76]}$$

Clearly, χ_{ij} is invariant under the gauge transformation.

In the large N limit, the T-matrix of conduction electron is expressed as $\mathcal{T}(\omega) = \Sigma_c(\omega)$ where

$$\Sigma_c(\boldsymbol{p}, i\omega) = -\frac{1}{N}\sum_{\boldsymbol{k},\nu} G_\chi(\boldsymbol{k}, i\nu) G_f(\boldsymbol{p}+\boldsymbol{k}, i\omega + i\nu). \qquad \text{[S77]}$$

To facilitate further discussion, we keep the momentum dependence in the expression of Σ_c although we can demonstrate that it is indeed momentum independent. Performing Fourier transformation for Σ_c, we reach

$$\begin{aligned} \Sigma_c(\boldsymbol{p}, i\omega) = &-\frac{1}{N}\sum_{\nu}\sum_{\boldsymbol{r}} G_\chi(\boldsymbol{r}_i - \boldsymbol{r}_j, i\nu) G_f(\boldsymbol{r}_j - \boldsymbol{r}_i, i\omega + i\nu) \times e^{i(\boldsymbol{r}_i - \boldsymbol{r}_j)\cdot\boldsymbol{p}} \\ &-\frac{1}{N}\sum_{\nu}\sum_{\boldsymbol{r}} \left\langle \hat{\chi}_{i\nu a}\hat{\chi}^\dagger_{j\nu a} f_{j,\omega+\nu,\alpha} f^\dagger_{i,\omega+\nu,\alpha} \right\rangle \times e^{i(\boldsymbol{r}_i - \boldsymbol{r}_j)\cdot\boldsymbol{p}}, \end{aligned} \qquad \text{[S78]}$$

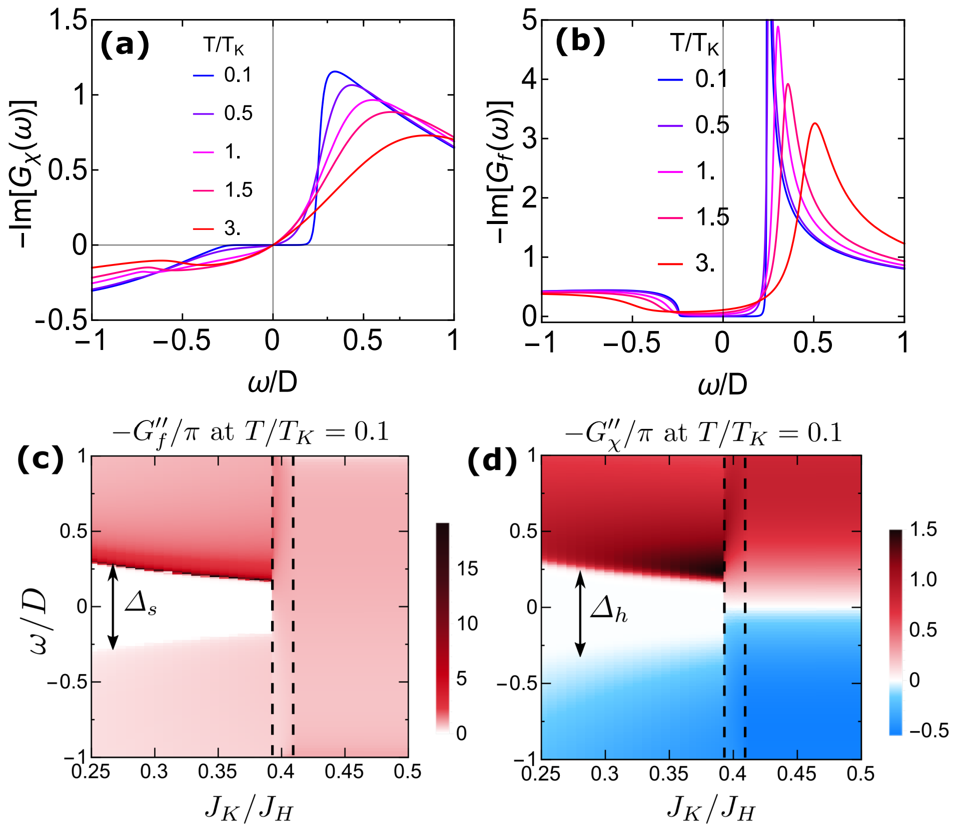

Fig. S7. Spectral weight as a function of ω/D. Spectral weight of (*A*) the Kondo hybridization field $\hat{\chi}$, $-G''_\chi$, and (*B*) the local f electron, $-G''_f$, with different temperatures for $\kappa = 0.3$ and $g = 0.29$. Density plots of the spectral weight of (*C*) the f-electron and (*D*) the $\hat{\chi}$ field for $\kappa = 0.3$ as a function of J_K/J_H. Inside the narrow region between the dashed lines is the coexisting (superconducting) phase.

where we have written $\boldsymbol{r} \equiv \boldsymbol{r}_i - \boldsymbol{r}_j$. We can verify that Eq. (S78) is invariant under the gauge transformation.

S.IX. Results of the channel-asymmetric large-N Kondo lattice model

In this section, we summarize some results for channel-asymmetric large-N Kondo lattice model with $\kappa = 1/2$ which are not included in the main text. We further present results of the channel-asymmetric large-N Kondo lattice model with $\kappa \leq 1/2$.

Finite-temperature phase diagram. The finite-temperature phase diagram of the channel-asymmetric Kondo lattice model for $\kappa = 0.3$, $J'_K = 2J_K$, and $J_K/D = 1.0$ ($T_K/D = 0.135$) is shown in Fig. S4. The metallic valence-bond-solid phase exists for small values of g ($\Delta \neq 0$, $x = 0$, the yellow, gray and orange regions), while the Kondo screened paramagnetic heavy-electron phase prevails at large g ($\Delta = 0$, $x \neq 0$, blue and green regions). A coexisting phase is found at intermediate g ($\Delta \neq 0$, $x \neq 0$, the pink region) (16, 17). This phase becomes an extended s-wave superconducting phase when electron baths are connected. A high-temperature decoupled phase is reached when $\Delta = x = 0$.

The T^* (blue) line sets the boundary between $x = 0$ and $x \neq 0$. In the FL phase, T^* corresponds to the mean-field Kondo coherence temperature below which the Bose-condensed Kondo hybridization develops phase coherence over the lattice, with a value $T^* \sim 2.8\,T_K$, consistent with experimental observation that $T_K \ll T^*$ (18). At lower temperatures $T < T_{FL} \approx 1.2\,T_K$, the system becomes a Fermi liquid where specific heat coefficient reaches a constant at $T \sim T_{FL}$. At $g < g_c \approx 0.39$ and at temperatures below T_{SL}, the system develops gaps (Δ_s, Δ_h) in both the spinon and holon spectral functions (see Fig. S4)) where the thermodynamical observables and transport show an exponential decay as $T \to 0$, see Figs. S5 and S6. Interestingly, for temperatures above T_{SL}, a NFL strange metal region (the gray area in Fig. S4) is found, characterized by a quasi-linear-in-temperature dependence of the scattering T-matrix (see detailed T-matrix calculations below).

First-order transition. Fig. S7 shows the spectral weight of the f and $\hat{\chi}$ fields at $\kappa = 0.3$. At $g < g_c$, the density plots of the spectral functions at temperature $T/T_K = 0.1$ show a gap which closes abruptly at $g = g_c$, indicating a first-order transition (19, 20). The NFL strange metal state is linked to the end-point (g_{QC}, T_{QC}) of this finite-temperature first-order transition line (the black dotted curve in Fig. S4), suggesting a quantum-critical end point associated with g_{QC} when T_{QC} is suppressed to zero (21, 22). An observed discontinuity in the entropy also reflects this first-order transition, see Fig. S8(*B*).

T-matrix and the strange metal state. The T-matrix of conduction electron, defined in Eq. (S28), reduces to $\Sigma_c(\omega) \sim \mathcal{O}(1/N)$ in the $N \to \infty$ limit. Strikingly, the static T-matrix, corresponding to the scattering rate, $\tau^{-1}(\omega = 0, T) = -N\mathcal{T}''(\omega = 0, T)$,

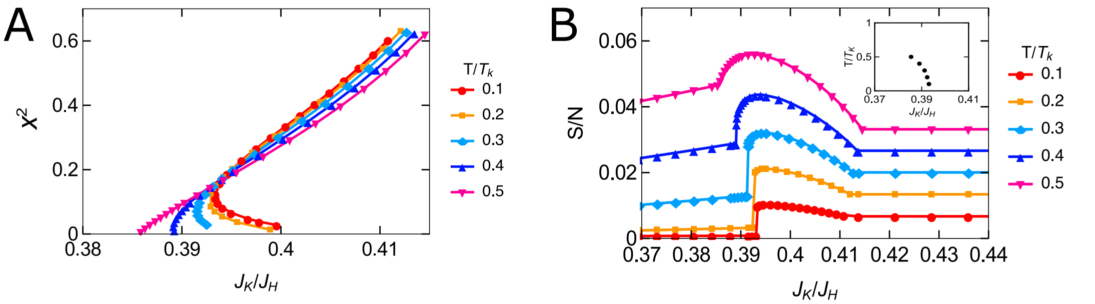

Fig. S8. Signatures of first-order transition. (*A*) x^2 and (*B*) entropy as functions of g at various temperatures with $\kappa = 0.3$ for the large-N limit.

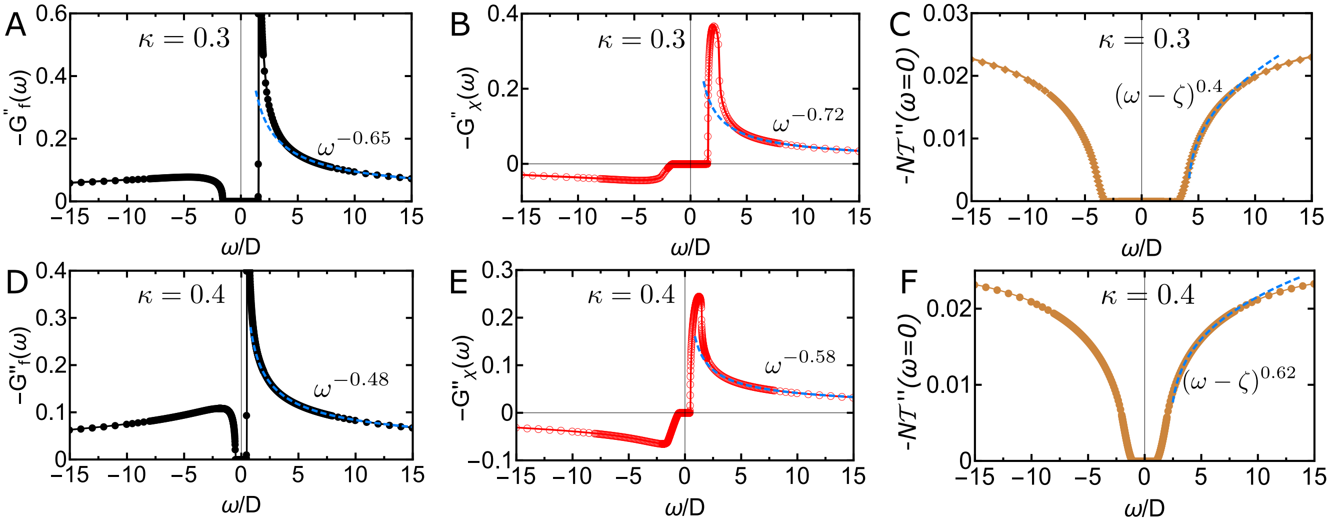

Fig. S9. Power-law behavior for spectral function and T-matrix. (*A*) $-G''_f$, (*B*) $-G''_\chi$, and (*C*) $-N\mathcal{T}''$ for $\kappa = 0.3$ exhibit power-law-in-ω behavior at frequency slightly higher than the spinon gap. Figures (*D*)-(*F*) correspond to $\kappa = 0.4$. The blue dashed lines here represent power-law fitting. Here, ζ in Figures (*C*) and (*F*) is a fitting parameter with its value close to the size of spinon gap Δ_s (see Fig. S7).

displays NFL behavior with a quasi-linear-in-T power-law scaling (see Fig. S5*A*) in the intermediate temperature range, i.e. $-N\mathcal{T}''(\omega = 0, T) \sim T^{1-p(\kappa)}$ with $0 < |p(\kappa)| < 1$ over a wide range in temperatures (see gray region of Fig. S4 for $\kappa = 0.3$). The T-super-linear to T-sub-linear power-law crossover in T-matrix with continuously increasing κ comes as a result of enhanced electron scattering at low temperatures via Kondo fluctuations with softened gaps. Following the continuous evolution of the power-law exponents of T-matrix with the change in κ, we found a T-linear dependence of T-matrix at $\kappa \approx 0.3$, a typical NFL strange metal signature (see Fig. S5*B*). This NFL region shows quantum critical behavior associated with the KB transition at (g_{QC}, T_{QC}) via an universal scaling form of $-N\mathcal{T}''(\omega = 0, T)$, indicated by the scaling of the constant (flat) temperature derivative $d/dT[-N\mathcal{T}''(\omega = 0, T)]$ (inset of Fig. S5*C*) and the power-law-in-$|g - g_{QC}|$ vanishing crossover scales with respect to T_{QC} on both sides of the transition (see black dashed lines in Fig. S4 and the inset therein), below which the NFL state disappears. Similar quasi-linear power-law feature is also found in the dynamical T-matrix: $-N\mathcal{T}''(\omega, T = 0) \propto (\omega - \Delta_s)^{1-p(\kappa)}$. We attribute this strange metal feature to the critical Kondo fluctuations coupled to gapless fermionic spinons, manifested by the quasi-linear-in-ω power-law behavior in holon and spinon spectral functions outside the gaps (see Figs. S9*A*, *B*, *D*, and *E*). In the gapped spin-liquid region as κ is away from $\kappa_c = 1/2$, however, the T-matrix decays exponentially (see Fig. S5*C*). The exponential decay in the scattering T-matrix indicates the development of spinon gap $\Delta_s > 0$ of the spin-liquid phase. However, as $|\kappa - \kappa_c|$ decreases, Δ_s is suppressed in a power-law fashion as a function of $|\kappa - \kappa_c|$, $\Delta_s \propto |\kappa - \kappa_c|^\nu$. See Fig. 4*B* of the main text. Here, we find $\nu \approx 1.55$. Suppression of Δ_s as κ approaches κ_c is indicated in the T-matrix (see Fig. 4*C* of the main text).

Here, we would like to make some remarks: (i) when the conduction-electron baths are connected, the quasi-linear-in-T strange metal feature in T-matrix (results are shown in Fig. S5 and Fig. 2*A* of the main text) indicates a quasi-linear-in-T electrical resistivity ρ via the Boltzmann equation of the electrical conductivity $\sigma(T) = 1/\rho(T) \sim \int \tau(\omega)\partial_\omega n_F(\omega) d\omega$ with $n_F(\omega)$ being the Fermi function. (ii) Moreover, we expect that including electron hopping and $O(1/N^2)$ diagrams of Σ_c in our calculation of $\mathcal{T}$-matrix will restore the Fermi-liquid behavior with T^2 electrical resistivity in the $T \to 0$ limit in the Kondo phase and in the gapped RVB phases (23).

The power-law behavior of T-matrix. For $\kappa \leq 1/2$, in the intermediate frequency regime at low temperature, we show that the spectral densities of the f electrons and holons exhibit power-law-in-ω behavior $G''_f(\omega) \sim \omega^{-a}$, $G''_\chi(\omega) \sim \omega^{-b}$, where $0 < a$, $b < 1$ (see Fig. S9). This quasi-linear power-law behavior in G''_f and G''_χ gives rise to power-law behavior in the

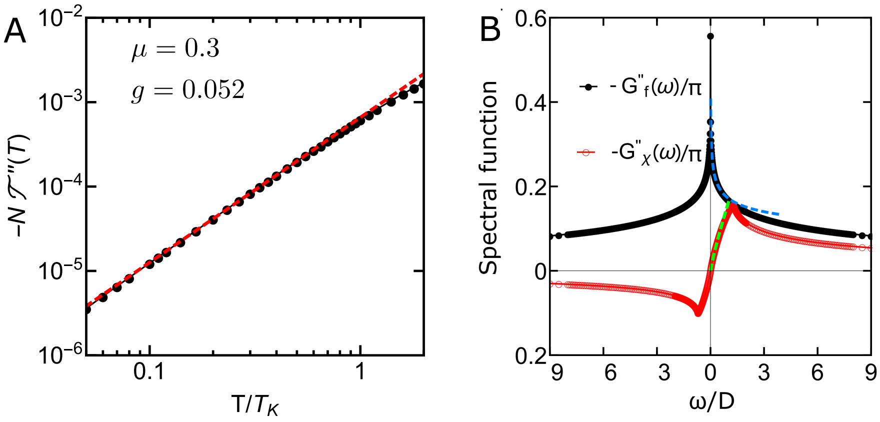

Fig. S10. Robustness of the strange metal phase against PH asymmetry of the conduction band. (*A*) Static T-matrix as a function of T/T_K for the PH asymmetric case with chemical potential of conduction electron $\mu/D = 0.3$ fixed and fixed $g = 0.052$. The low-T regime of static T-matrix shows the same power-law-m-T behavior with the PH symmetric case, i.e. $-N\mathcal{T}''(T) \sim T^{1.7}$, indicated by the red dashed line. (*B*) Spectral functions of the spinon and holon field with the PH asymmetry: $-G''_f(\omega)/\pi \sim \omega^{-0.19}$ (blue dashed line) and $-G''_\chi(\omega)/\pi \sim \omega^{0.84}$ (green dashed line) at low frequency limit for $\kappa = 1/2$ with $g = 0.052$, $\mu/D = 0.3$ and $T/T_K = 0.05$ fixed.

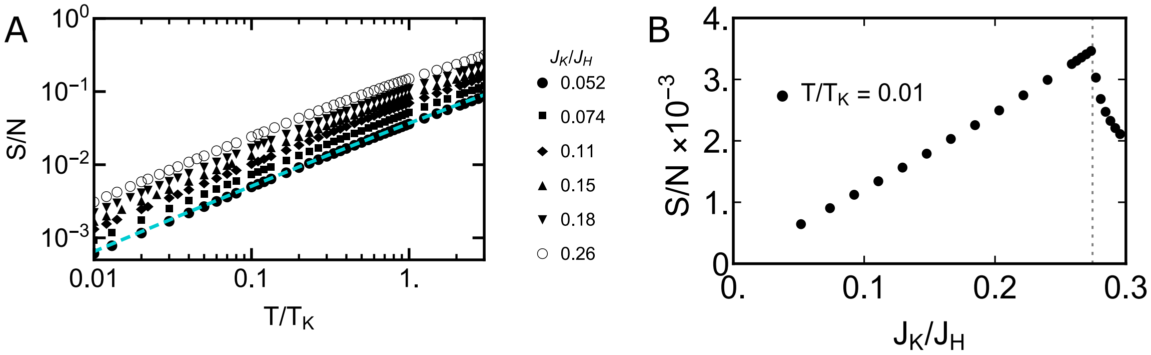

Fig. S11. Entropy for $\kappa = 1/2$. (*A*) Entropy S/N as a function of T/T_K with different values of g. The blue dashed line in (*A*) is a fit to the function of $a[T + T\ln(T_1/T)]$ with a and T_1 being non-universal constants. (*B*) Entropy versus J_K/J_H for $T/T_K = 0.01$ fixed. The vertical dashed line represents $g = g_{QC}$.

conduction electron T-matrix in certain frequency range,

$$-N\mathcal{T}''(\omega, T = 0) \sim (\omega - \zeta)^{1-p(\tau)} \quad [S79]$$

with $0 < p(\tau) < 1$ with ζ being a fitting parameter with its value close the size of the spinon gap, Δ_s (see Figs. S7 and S9), and leads to the similar quasi-linear-in-temperature behavior of T-matrix, as shown in Fig. S5(A).

For $\kappa = 1/2$, this distinct power-law behavior in the static T-matrix, $-N\mathcal{T}''(\omega = 0, T \to 0)$, at the low-temperature regime can be analytically estimated via simple power counting of the ω power-law dependence of $-G''_f(\omega \to 0)$ and $-G''_\chi(\omega \to 0)$, shown in Fig. 1(C) of the main text, as follows: For $\omega = 0$, Eq. (S30) becomes

$$-N\mathcal{T}''(\omega = 0, T) = \int \frac{dz}{\pi} \mathrm{csch}(\beta z) G''_f(z) G''_\chi(z) \quad [S80]$$

At low temperatures (large β), the function $\frac{1}{e^{\beta z} - e^{-\beta z}}$ is finite only at small z. Therefore, only $G''_f(z)$ and $G''_\chi(z)$ at small z contribute to this integral.

According to Fig. 1(C) of the main text, one has $-G''_f(z) \sim z^a$, $-G''_\chi(z) \sim z^b$ with $a = -0.19$ and $b = 0.84$ for $\kappa = 1/2$, we have

$$\begin{aligned} -N\mathcal{T}''(\omega = 0, T) &\propto \int \frac{dz}{\pi} \frac{2}{e^{\beta z} - e^{-\beta z}} z^{a+b} \\ &\propto T^{1+a+b}, \end{aligned} \quad [S81]$$

leading to $-N\mathcal{T}''(\omega = 0, T) \sim T^{1-0.19+0.84} = T^{1.65}$, in excellent agreement with the results shown in Fig. 2(A) of the main text. We would like to make a remark: the above analysis does not work at high temperatures where the whole integral range plays a role. Since $G_\chi(z)$ changes its behavior at $z = 1$, the characteristic cross temperature is estimated to be $T \sim 1/\beta \sim 1$.

Robustness of the strange metal phase against PH asymmetry of the conduction band. When only conduction bath breaks particle hole symmetry by shifting its chemical potential away from half-filling, we find the power-law behavior

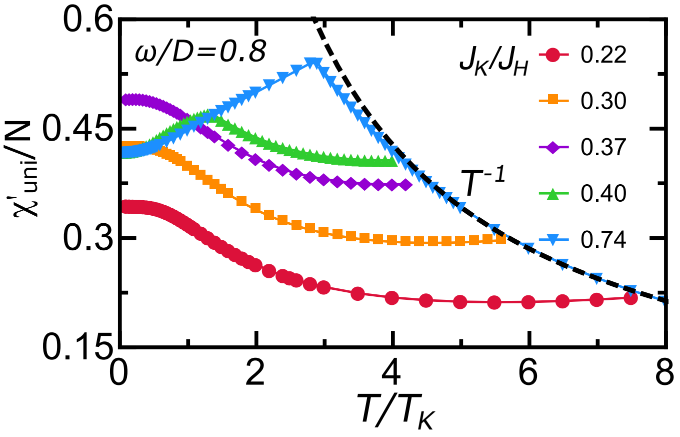

Fig. S12. Dynamical spin susceptibility χ_{uni}/N. The real part of χ'_{uni}/N versus temperature with $\kappa = 0.3$ for various values of g.

of the spinon and holon spectral functions in the low-frequency regime remain qualitatively unchanged, indicating that the power-law-in-T strange metal features of the static T-matrix still persist as $T \to 0$ (see Fig. S10). This suggests our results are robust against particle-hole asymmetry of the local conduction bath. Nevertheless, the spinon and holon spectral functions in the intermediate-frequency regime show some PH asymmetry, see Fig. S10(B).

Entropy and Specific heat coefficient. Fig. S8(B) shows our result of entropy S/N as functions of g with different temperatures. Both the entropy and Kondo hybridization condensate x between $0.38 < g < 0.4$ show a jump in the temperature range $T/T_K < 0.5$. This jump suggests a first-order transition between the RVB spin-liquid phase and the coexisting (superconducting) phase at low temperatures. The first-order transition stops at $(g,\, T/T_K) = (g_{QC},\, T_{QC})$, see the black-dotted line in Fig. S4. At temperatures above the first-order transition end-point, $T > T_{QC} \approx 0.5$, the transition becomes continuous and the two crossover scales on both sides of the end-point exhibit power-law-in-$|g - g_{QC}|$ vanishing behavior with respect to $(g_{QC},\, T_{QC})$ (see Fig. S4 and the inset therein). This first-order transition is identical to the metal-to-superconductor transition described in Ref. (20). The transition is expected to be continuous when superconductivity is suppressed by magnetic field.

In Figs. S6A and S6B, we calculate the specific heat coefficient, $C_V/(NT) = (N^{-1})\partial S/\partial T$ for $\kappa = 0.3$. In the RVB phases, with decreasing temperatures, $C_V/(NT)$ shows a Schottky-like peak, followed by an exponential decay at low temperatures due to the finite spinon gap (black-dashed line in Fig. S6A), and finally exhibits a metallic feature with a saturated value. For $g = 0.4$, a peak at $T/T_K \approx 1.2$ is observed, indicating that the system enters the coexisting phase. However, as temperature further decreases, the specific heat coefficient reaches a Fermi-liquid-like saturated value instead of decaying to zero as it does for a typical superconductor. We suspect that this is an artifact of our approach due to the independent conduction bath, such that the coexisting state is not a *true* superconducting state. In the Kondo-screened phase, however, the Fermi-liquid behavior is clearly manifested by the constant $C_V/(NT)$ with $T_{FL} \sim T_K$ (Fig. S6B). A Schottky peak at T^* is observed, which is due to an artefact of our dynamical large-N mean-field approach.

Figs. S11A and S11B show the entropy as a function of T/T_K and g, respectively, in the strange-metal phase ($g = 0.052$) for $\kappa = 1/2$. The entropy with g fixed exhibits a NFL property: it decreases as $S/N \sim a[T + T\ln(T_1/T)]$ with decreasing temperatures , where a and T_1 are fitting parameters (blue dashed line in Fig. S11A). Due to the quantum critical nature of the strange metal phase for $\kappa = 1/2$, we expect the entropy will saturate at a value much smaller than $(1/2)\ln 2$ in the $T \to 0$ limit. However, it goes beyond our present numerical capability to confirm this expectation. Description of the quantum critical nature of the strange metal phase is provided in the Section "The quantum critical strange metal phase" of the main text. The NFL behavior of entropy leads to the typical NFL logarithmic-in-T divergence in specific heat coefficient: $C_V/NT \propto -\ln(T)$ (see Figs. 3A-3C of the main text). Regarding the entropy as a function of g for $\kappa = 1/2$ at low temperature $T/T_K = 0.01$ fixed (Fig. S11B), in contrast to the $\kappa = 0.3$ case of Fig. S8B, however, we found that this entropy does not show a discontinuity (jump), consequently a continuous phase transition at g_{QC}.

Dynamical spin susceptibility. Fig. S12 shows the temperature dependence of the real part of the uniform dynamical spin susceptibility, $\chi'_{uni}(\omega, T) \equiv \mathrm{Re}[\chi_{uni}(\omega, T)]$, at a fixed frequency ω of the local moments for $\kappa = 0.3$. On the FL side and at high temperatures ($\Delta = 0 = x$), $\chi'_{uni}(\omega, T)$ shows a $1/T$ Curie law of local spin moment; while it develops a kink at $T = T^\star$ and decreases for $T < T^*$ to a saturated value as $T \to 0$ due to the formation of Kondo singlet. Interestingly, $\chi'_{uni}(\omega > \Delta_s, T)$ shows an increase with decreasing temperatures and tends to saturate at low temperatures, a signature of fermionic spin-liquid (24).

For $\kappa = 1/2$, similar $-\ln(T)$ dependence is found in both static and dynamical spin susceptibility at low temperature (see Figs. 3D and 3E of the main text). Reminiscent of the case of $\kappa = 0.3$, the dynamical spin susceptibility at a fixed frequency ω_0/D also saturates at low temperatures (see Fig. 3E of the main text for the cases), suggesting the fermionic nature of critical spin-liquid.

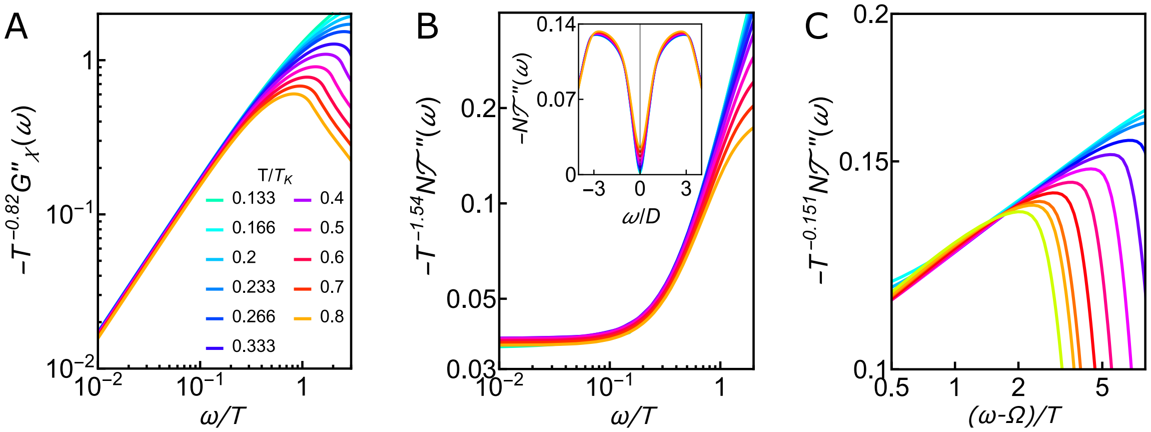

Fig. S13. ω/T scaling at the large-N limit for $\kappa = 1/2$ and $g = 0.26$. ω/T scaling of (*A*) $-G''_{\chi}(\omega)$, (*B*) $-N\mathcal{T}''(\omega)$ at the lower-frequency regime and (*C*) $-N\mathcal{T}''(\omega)$ at the intermediate-frequency. In (*C*), Ω is a fitting parameter. Inset of (*B*) shows the unscaled T-matrix.

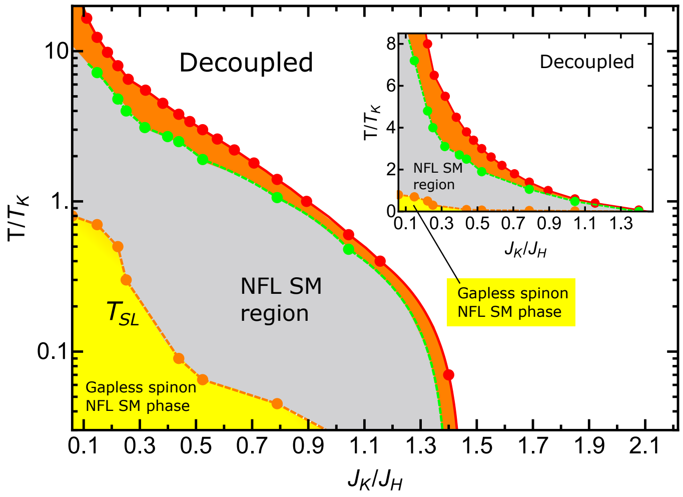

Fig. S14. The finite-temperature phase diagram of the channel-symmetric large-N Kondo lattice model for $\kappa = 0.5$. Different colors correspond to regions with different behaviors of physical quantities. Due to symmetric Kondo coupling on all channels, the Kondo-screened Fermi-liquid phase vanishes. Inset shows the same phase diagram in linear scale.

ω/T scaling. It has been known that ω/T scaling of observables often exists at a quantum critical point. However, we find that in additional to the ω/T scaling we observed deep inside the gapless RVB spin-liquid strange metal phase (see Fig. 2*B* of the main text), we also find the ω/T scaling for $-G''_{\chi}(\omega)$ and $-N\mathcal{T}''(\omega)$ near the QCP ($g = 0.26$), see Fig. S13. The fact that ω/T scaling extends over a wide range in the values of g in the gapless spin-liquid phase further supports the quantum critical nature of this entire exotic phase.

S.X. Results of the channel-symmetric large-N Kondo lattice model

In this section, we demonstrate the finite-temperature phase diagram (Fig. S14) of the channel-symmetric large-N Kondo lattice model with $\kappa = 1/2$ where, in our calculation, the Kondo couplings of all channels are set to be the same, i.e. $J'_K = J_K$. Our numerical results show that the Kondo-screened Fermi-liquid state with finite Bose-condensed Kondo hybridization ($x \neq 0$) does not exist. This result is consistent with the absence of Fermi-liquid state in the related channel-symmetric single-impurity Kondo model, discussed in Ref. (8): Therein, the Fermi-liquid phase is unstable against the overscreened non-Fermi liquid state due to channel symmetry. The finite-temperature phase diagram Fig. S14 is only consisted of a RVB spin-liquid state and a decoupled phase, but the Kondo-screened Fermi-liquid phase is absent. Since the Fermi-liquid phase vanishes, the RVB dominated regime (the RVB spin-liquid strange-metal phase and the RVB NFL region) extends to a large value of J_K/J_H and it terminates at the transition to the decoupled phase. The crossover scales of the RVB mean-field (red dotted line), the RVB NFL region (green-dotted line), and RVB strange-metal phase (orange-dotted line) converge to the same critical point as $T \to 0$ (see the inset in Fig. S14). The qualitative features of the RVB dominated regime (the RVB spin-liquid strange-metal phase and the NFL region) still remain the same as that for the channel-asymmetric case.

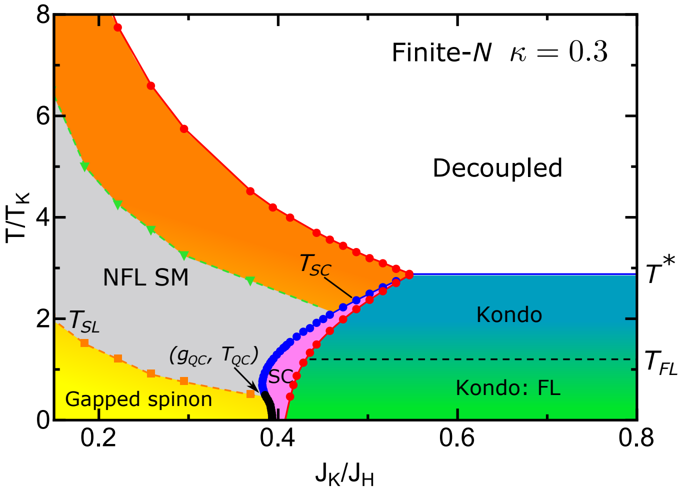

Fig. S15. Finite temperature phase diagram with inclusion of finite-N corrections of the channel-asymmetric Kondo lattice model with $J'_K = 2J_K$, $N = 10$, $K = 3$, and $\kappa = 0.3$. The yellow, gray, and orange regions are dominated by the RVB order parameter, while the blue and green regions refer to the Kondo-screened heavy electron phase (below the T^* line). The pink region is a coexisting (superconducting) phase, while white region is a paramagnetic local moment phase. $(g_{QC},\, T_{QC})$ is the end-point of the crossover of the gapped valence-bond phase. The Fermi-liquid phase exists for $T < T_{FL}$ (green area), while the NFL region is bounded by the green and orange curves (gray area).

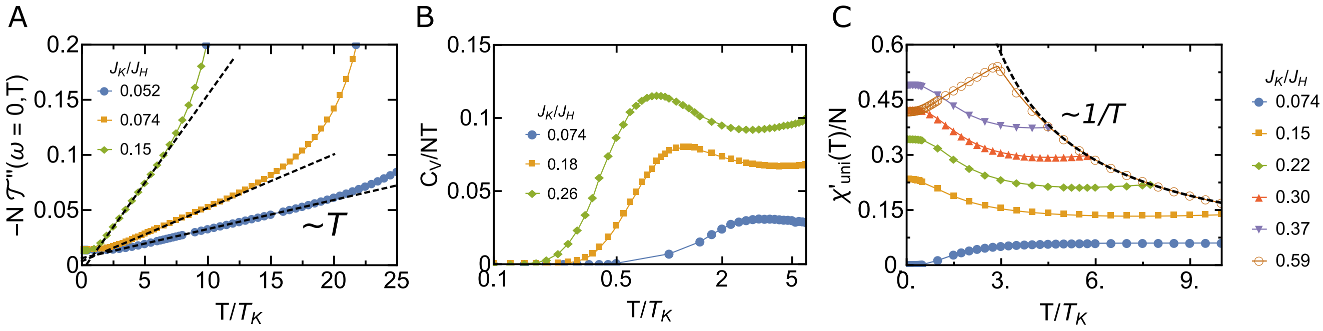

Fig. S16. Observables with the inclusion finite-N corrections for $\kappa = 0.3$, $K = 3$ and $N = 10$. (*A*) $-NT''(\omega = 0, T)$ shows a linear-in-temperature dependence (black dashed lines) in intermediate temperature range in the RVB phase. (*B*) Temperature dependence of specific heat coefficient, C_V/NT. (*C*) The real part of the dynamical susceptibility χ'/N at fixed frequency $\omega/D = 0.8$.

S.XI. Finite-N calculations

In this section, we solve the dynamical Schwinger-Dyson equations of the two-dimensional Kondo-Heisenberg lattice model in the multichannel pseudofermion representation with N and K being finite. We further compare the finite-N results with that for the infinite-N case.

For finite-N, K, in principle, one needs to consider the full Green's function and the self-energies given in Eqs. (S12)-(S20) above. But this is a technically challenging task, and will be addressed in our future work. To simplify the problem, we keep only the leading $\mathcal{O}(1)$ Green's functions and the leading order in $1/N$ correction of the self-energy Σ_c of the conduction electrons. Our approximation is sufficient to address the leading $1/N$ corrections to our large-N solutions in the spin-liquid phase which we focus here.

The equations for G_c and Σ_c can be obtained as

$$\begin{aligned} G_c^{-1}(\omega) &= G_{c0}^{-1}(\omega) - \Sigma_c(\omega), \\ \Sigma_c(\omega) &= -\frac{1}{N}\sum_{\nu} G_\chi(\nu) G_f(\omega+\nu). \end{aligned} \qquad [S82]$$

In addition to the inclusion of self-consistent equations of G_c and Σ_c, we also have to replace G_{c0} by G_c which appears in other self-consistent equations of the infinite-N case.

Results of $N = 10$, $K = 3$ ($\kappa = 0.3$). Using these self-consistent equations, we map out the finite-temperature phase diagram of the finite-N, K channel-asymmetric Kondo lattice model with $N = 10$ and $K = 3$ ($\kappa = K/N = 0.3$) and calculate various physical quantities. We choose the following parameters in this calculation: $J'_K = 2J_K$, $J_K/D = 1.0$ ($T_K/D = 0.135$). The results are briefly described in the following:

Finite-temperature phase diagram-The phase diagram at finite-N shares close resemblance with that of the large-N limit, except for some quantitative differences: for example, the crossover scale separating the gapped valence-bond-solid and the NFL state is slightly suppressed for the finite-N case [see the orange dot-dashed line in Figs. S15 and S4]. At the FL side, the two observed crossover scales $T^*/T_K \sim 2.8$ and $T_{FL}/T_K \sim 1.2$ are almost identical to the infinite-N results.

T*-matrix*- The conduction electron scattering T-matrix is obtained via Eq. (S28). The imaginary part of the static T-matrix versus temperature for the finite-N case is shown in Fig. S16(A). We observe that the linear-in-T behavior is also preserved [black dashed lines in Fig. S16(A)] in the intermediate temperature range (the gray area bounded by green and orange curves in Fig. S15), suggesting a T-linear scattering rate of conduction electrons at low temperatures for the finite-N case.

Specific heat coefficient- The specific heat coefficient is displayed in Fig S16(B). In the gapped spin-liquid phase, as temperature becomes lower, $C_V/(NT)$ exhibits a broad peak at intermediate temperature range, followed by a metallic-like saturation at low temperatures due to fermionic excitations, similar to the results for the large-N case.

Dynamical magnetic susceptibility- The real part of the dynamical uniform susceptibility $\chi'_{uni}(\omega, T)/N$ at finite-N is illustrated in Fig. S16(C). In the RVB-dominated region, $\chi'_{uni}(\omega, T)/N$ with $\omega > \Delta_s$ increases with decreasing temperatures and saturates as $T \to 0$. At large value of g (e.g. $g = 0.59$), the uniform dynamical susceptibility displays a $1/T$ Curie-law behavior in the high-temperature local moment phase and a Pauli-like saturation in the FL state.

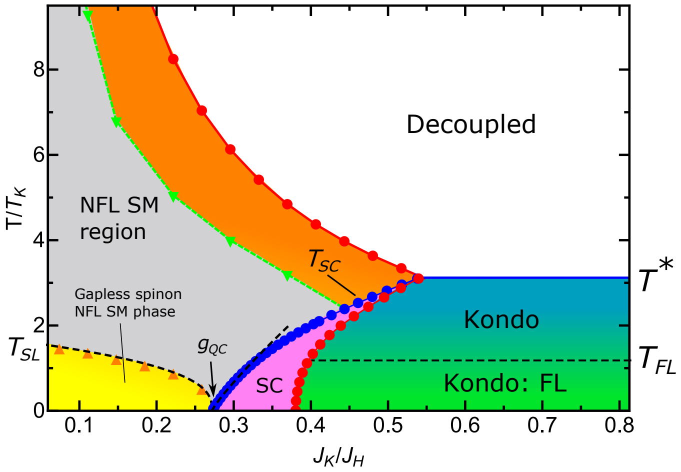

Fig. S17. The finite-temperature phase diagram of the $N = 4$, $K = 2$ ($\kappa = 1/2$) channel-asymmetric (with $J'_K = 2J_K$) Kondo lattice model. The black dashed lines on both side of g_{QC} are the power-law fit to the crossover scales.

Results for the $N = 4$, $K = 2$ ($\kappa = 1/2$) channel-asymmetric Kondo lattice. In this subsection, we summarize the results of the finite-temperature phase diagram (Fig. S17) and physical observables (Fig. S18) we obtained for the channel-asymmetric finite-N, K ($N = 4$, $K = 2$, $\kappa = K/N = 1/2$) Kondo lattice model. Here, we enforce the PH symmetry of the conduction band by eliminating the real part of the conduction electron self-energy Σ_c, defined in Eq. (S82). The finite-temperature phase diagram and all the physical observables, described in the following, show qualitatively the same feature as that for the large-N, channel-asymmetric case, $\kappa = 1/2$ case (see main text):

Finite-temperature phase diagram: The finite-temperature phase diagram for finite-N, K with $\kappa = 1/2$ is depicted in Fig. S17. In stark contrast to the finite-N, K case for $\kappa = 0.3$, the first-order transition is absent here, same with large-N, $\kappa = 1/2$ case [see Fig. 1A of the main text]. Like in the large-N, $\kappa = 1/2$ case, the signature of quantum-critical behavior near g_{QC} is also manifested by the power-law-in-$|g - g_{QC}|$ crossovers on both sides of g_{QC} (see the black dashed lines in Fig. S17).

Entropy and Specific heat coefficient: Figs. S18A and S18B show the entropy and specific heat coefficient as a function of T/T_K with different values of J_K/J_H in the RVB side for $K = 2$ and $N = 4$ ($\kappa = 1/2$), respectively. As with the result of the large-N, $\kappa = 1/2$ case, the entropy exhibits qualitatively the same NFL property with $S/N = a[T + T\ln(T_0/T)]$ with a and T_0 being the fitting parameters in the gapless spin-liquid strange-metal phase (blue dashed line in Fig. S18A). This gives rise to the typical NFL logarithmic-in-T divergence in specific heat coefficient: $C_V/NT \propto -\ln(T)$ (see the red dashed line of Fig. S18B and Figs. 3A to 3C of the main text).

T*-matrix*: Fig. S18(C) shows $-N\mathcal{T}''(\omega = 0, T)$. It exhibits a NFL sublinear-in-temperature $[-N\mathcal{T}''(T) \sim T^{0.7}]$ behavior at the intermediate temperature regime, followed by a superlinear-in-T $[-N\mathcal{T}''(T) \sim T^{1.7}]$ behavior down to very low temperatures. Similar behavior has been found in the large-N result, as shown in Fig. 2A of the main text.

Dynamical spin susceptibility: Fig. S18(D) shows the temperature-dependent real part of the uniform dynamical spin susceptibility, $\chi'_{uni}(\omega, T)/N$, of the local moments with different values of ω/D for $g = 0.074$. The dynamical spin susceptibility exhibits a NFL strange metal property with singular logarithmic-in-temperature behavior when $\omega/D \to 0$: $\chi'_{uni}(\omega, T)/N \sim -\ln(T)$ [blue dashed line in Fig. S18D]. Similar fermionic spin-liquid behavior at finite frequency, as has been found in Fig. S12, is also observed here: $\chi'_{uni}(\omega, T)$ shows an increase with decreasing temperatures and saturates at low temperatures.

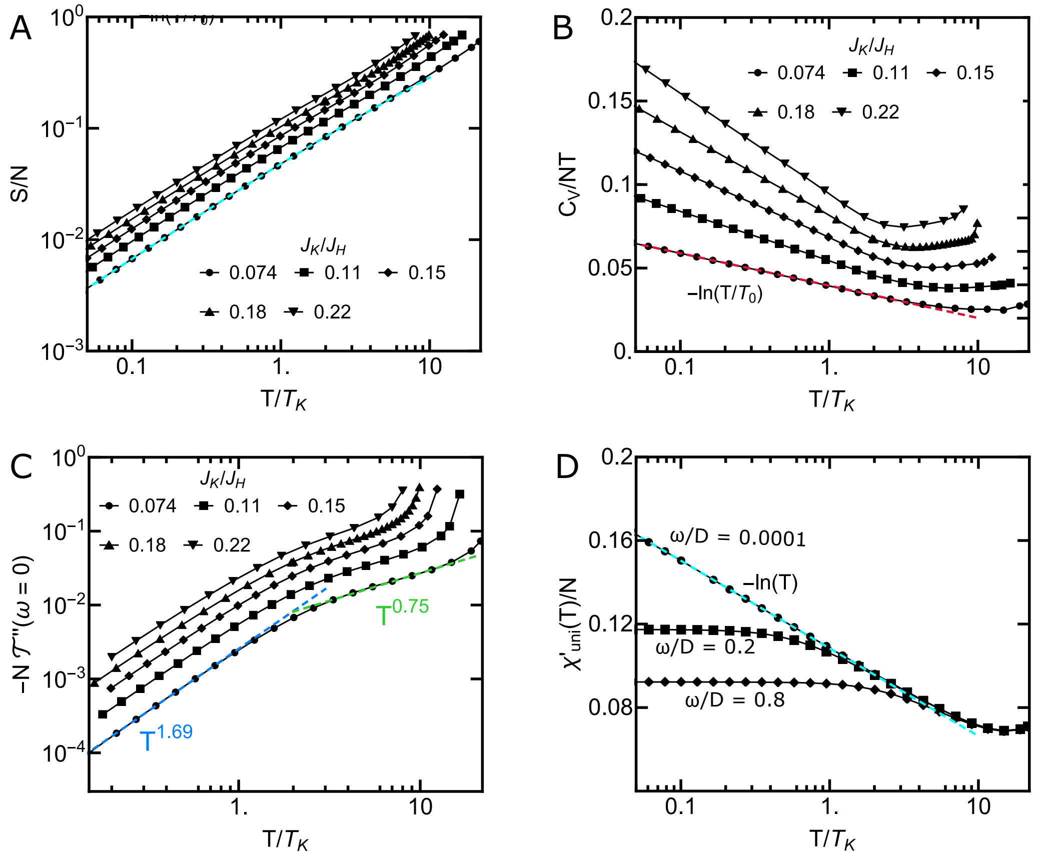

Fig. S18. Physical observables for the channel-asymmetric $N = 4$, $K = 2$ ($\kappa = 1/2$) Kondo lattice model. (*A*) Entropy S/N as a function of T/T_K with different values of g. The blue dashed line in (*A*) is a fit to the function of $a[T + T\ln(T_0/T)]$ with a and T_0 being non-universal constants, (*B*) specific heat coefficient (the red dashed line represents $-\ln(T/T_0)$ fitting), (*C*) imaginary-part of the scattering T-matrix as a function of T/T_K with different values of g, and (*D*) the real part of the uniform dynamical spin susceptibility χ'_{uni}/N with different values of ω/D for fixed $g = 0.074$. Here, the blue dashed line is a logarithmic fit.

Results for $N = 2$, $K = 1$ ($\kappa = 1/2$), physical SU(2) limit. Following a similar approach, we obtain the finite-N, K results for the single-channel Kondo-Heisenberg model ($K = 1$) in the physical limit with $N = 2$, $\kappa = 1/2$ (SU(2) and $S = 1/2$ limit), and $J_K/D = 1.0$ ($T_K/D = 0.135$).

Note that, in the physical limit, following the way we decouple the Kondo hybridization (holon) field in the multichannel case (see Eq. (S6) and *Results* in the main text), χ_{ia} field in the single-channel case here is either a Bose-condensed mean-field ($\chi_{ia} = x$) in the Kondo and coexisting phases or a pure fluctuating quantum field $\chi_{ia} = \hat{\chi}_{ia}$ in the other phases. The finite-temperature phase diagram of the $N = 2$, $K = 1$ ($\kappa = 1/2$) Kondo lattice model is shown in Fig. S19. It shows Kondo, coexisting, RVB dominated spin-liquid, and decoupled phases, qualitatively the same as that for finite N, K ($N = 4, K = 2$, see Fig. S17), and large-N, K (see Fig. 1(*A*) of the main text). We notice a minor quantitative difference near the phase boundary between the strange metal and coexisting superconducting (SC) phases compared to the above-mentioned cases with different values of N, K: The crossover line T_{SL} does not merge into the KB QCP at ground state. We think this may be due to the mean-field approximation ($\chi_{ia} = x$) we used in the Kondo and coexisting phases. The physical observables, the scattering T-matrix, the specific heat coefficient, and the dynamical spin susceptibility of the pure RVB spin-liquid phase are shown in Fig. S20. All physical observables in this case show qualitatively the same behavior as compared with the large-N and the finite-N ($N = 4$, $K = 2$) results.

In summary, we have performed self-consistent calculations of the 2D channel-asymmetric Kondo-Heisenberg lattice model for finite N and K. The finite-temperature phase diagram, $\chi'_{uni}(\omega, T)/N$, C_V/NT, and $-N\mathcal{T}''(\omega = 0, T)$ we obtained here show qualitatively the same behavior as that in the large-N, K limit. We thus conclude that our results in the RVB regions and in the coexisting superconducting phase are stable against the $\mathcal{O}(1/N)$ fluctuations of the conduction-electron self energy. Moreover, we expect that including electron hopping and the $O(1/N^2)$ diagrams of Σ_c in our self-consistent calculations will restore the Fermi-liquid behavior with T^2 electrical resistivity in $T \to 0$ limit in the Kondo phase and in the gapped valence-bond-solid phases (23).

S.XII. Benchmarking the strange metal phase

In this section, we benchmark our results in the strange metal phase by comparing it to the quantum critical point of the two-impurity Kondo model, i.e. the two-site problem of our Kondo lattice model. The Hamiltonian of two-impurity Kondo

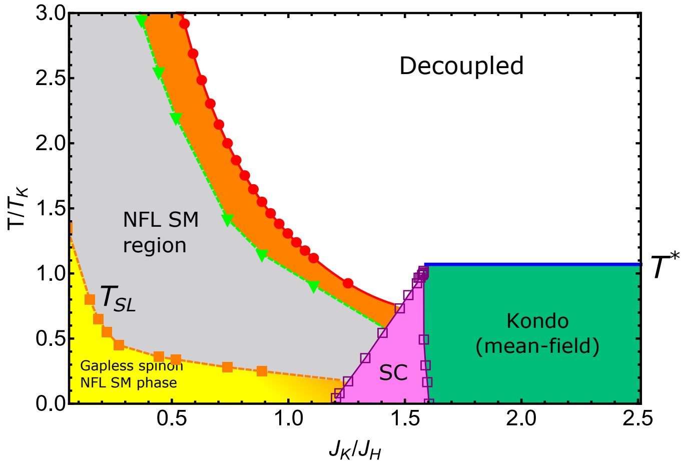

Fig. S19. The finite-temperature phase diagram of the finite-N, K ($N = 2$, $K = 1$) Kondo lattice model.

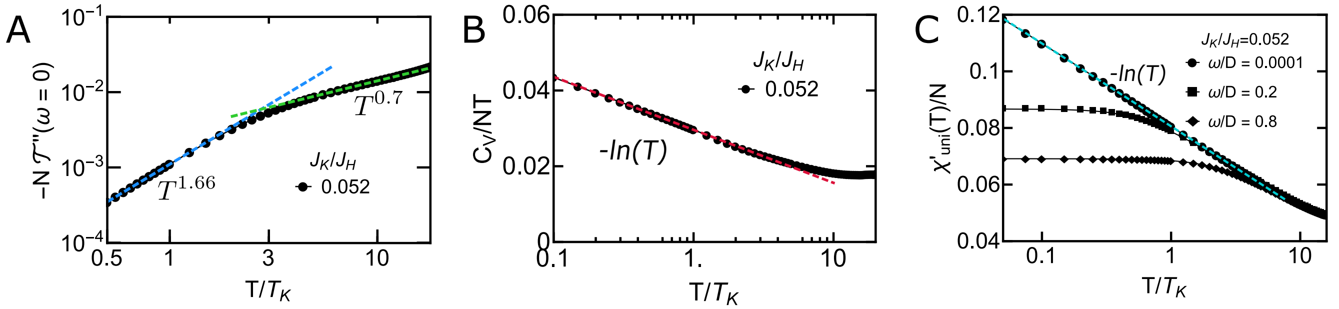

Fig. S20. Finite-N physical observables for $N = 2$, $K = 1$ ($\kappa = 1/2$) at $g = 0.052$. (*A*) Imaginary part of T-matrix (the blue and green dashed lines are power-law fits), (*B*) specific heat coefficient (the red dashed line represents $-\ln(T)$ fitting), and (*C*) Real part of the uniform dynamical spin susceptibility χ'_{uni}/N with different values of ω/D. Here, the blue dashed line is fit to $-\ln(T)$.

model can be written as

$$H_{2IK} = \sum_{i=1,2} H_0(i) + J_K \sum_{i=1,2} \mathbf{S}_i \cdot \mathbf{s}_i^c + J_H \mathbf{S}_1 \cdot \mathbf{S}_2, \tag{S83}$$

where $H_0(i)$ denotes the conduction electron bath at the impurity site i. The above model is slightly different from the original two-impurity Kondo model studied by B. A. Jones and C. M. Varma in Ref. (25, 26), where two spins are coupled to a single shared electron bath. The Jones-Varma two-impurity Kondo model shows a quantum critical point, known as the Jones-Varma QCP, separating the Kondo singlet phase from a (local singlet) valence bond phase where the two local impurity spins form a singlet with each other. The Jones-Varma QCP is known for its instability against PH asymmetry. However, it has been shown that the two-impurity Kondo model with independent electron baths has a QCP stable against PH asymmetry due to the absence of charge transfer between the two electron baths (27). We assumes two independent electron baths coupled separately to the two impurity spins in our two-impurity Kondo model, same as that in Ref. (27). This setup forbids charge transfer between the two impurity sites, allowing the QCP to persist even without particle-hole symmetry.

The Hamiltonian of our two-impurity Kondo model in the large-N, K limit reads

$$H_{2IK} = \sum_{ia} H_0^a(i) - \sum_{ia\alpha\beta} \frac{J_K^a}{N} f^\dagger_{i\alpha}\psi_{ia\alpha}\psi^\dagger_{ia\beta} f_{i\beta} - \frac{J_H}{N}\sum_{\alpha\beta}(\tilde{\beta} f^\dagger_{2,-\beta} f^\dagger_{1\beta})(\tilde{\alpha} f_{1\alpha} f_{2,-\alpha}). \tag{S84}$$

In the channel-symmetric limit, the Hamiltonian Eq. (S84) shows the Sp(N) × SU(K) symmetry. The Kondo and Heisenberg interactions can be decomposed via the Hubbard-Stratonovich transformation as

$$\begin{aligned} -\sum_{\alpha\beta} \frac{J_K^a}{N} f^\dagger_{i\alpha}\psi_{ia\alpha}\psi^\dagger_{ia\beta} f_{i\beta} &\rightarrow \frac{1}{\sqrt{N}}\sum_{ia}\left(\sum_\alpha f^\dagger_{i\alpha}\psi_{ia\alpha}\chi_{ia} + H.c. + \frac{|\chi_{ia}|^2}{J_K^a}\right), \\ -\frac{J_H}{N}\sum_{\alpha\beta}(\tilde{\beta} f^\dagger_{2,-\beta} f^\dagger_{1\beta})(\tilde{\alpha} f_{1\alpha} f_{2,-\alpha}) &\rightarrow \Delta^* \sum_\alpha\left(\tilde{\alpha} f_{1\alpha} f_{2,-\alpha} + H.c. + \frac{|\Delta|^2}{J_H}\right). \end{aligned} \tag{S85}$$

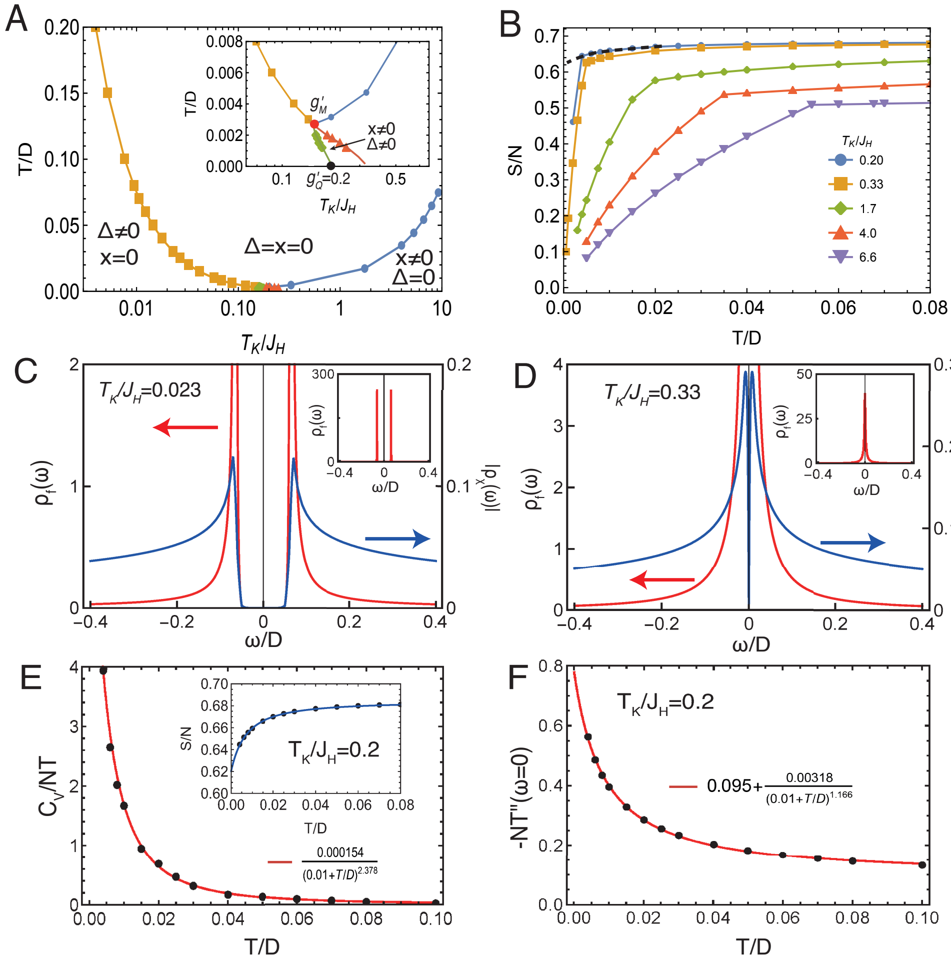

Fig. S21. Two-impurity Kondo model with $J'_K = 1.145 J_K$. (A) The phase diagram of the two-impurity Kondo model. The inset shows an enlargement of the critical region around $T_K/J_H = 0.16$. (B) Temperature dependence of the entropy for different values of T_K/J_H. The dashed curve is an extrapolation of the entropy of decoupled phase to zero temperature. (C) The density of states of f-spinons (red) and χ-holons (blue) in the valence bond phase ($T_K/J_H = 0.023$, $T/D = 0.003$). (D) The same plot as (C), but in the Kondo phase ($T_K/J_H = 0.33$, $T/D = 0.0005$). (E) The specific heat coefficient ($C_V/(NT)$) of the decoupled phase at $T_K/J_H = 0.2$. The red line shows a fit to the data points. The inset shows the entropy at the same parameter. (F) The scattering T-matrix ($-N\mathcal{T}''(\omega=0)$) of the decoupled phase at $T_K/J_H = 0.2$. The red line is a fit to the data points.

Similar to the lattice case, we explicitly break the SU(K) channel symmetry down to SU($K-1$) by setting $J_K^{a=K} = J'_K > J_K^{a\neq K} = J_K$, in order to obtain the Kondo Fermi-liquid phase. In the Kondo Fermi liquid phase, the holon field (χ_{ia}) condenses on the $a = K$ channel with $\langle \chi_{iK} \rangle \equiv x\sqrt{N} \neq 0$, while $\chi_{i,a\neq K}$ are treated as fluctuating fields.

Fig. S21(A) shows the phase diagram of our two-impurity Kondo model with $J'_K = 1.145 J_K$ and PH symmetry $\kappa = 1/2$. Similar to the lattice case, four regions can be found: a Kondo singlet phase ($x \neq 0$, $\Delta = 0$), a valence bond phase ($x = 0$, $\Delta \neq 0$), a decoupled phase ($x = \Delta = 0$) and a coexisting phase ($x \neq 0$, $\Delta \neq 0$). The coexisting phase can be significantly suppressed by fine-tuning J_K and J_H. In Fig. S21(A), we fix $J_K = 0.35$ and decrease J_H to find the finite temperature mean-field boundary of the (local singlet) valence bond phase, while we fix $J_H = 0.02$ and increase J_K to find the mean-field boundary of the Kondo singlet phase. The two boundaries merge at $J_K = 0.35$ and $J_H = 0.02$, corresponding to $T_K/J_H \approx 0.16$ (denoted as g'_M in Fig. S21A). We find a negligible coexisting phase in between the Kondo and local singlet phases (see Fig. S21(A). At zero temperature, the QCP between the valence bond phase and the coexisting phase is at $T_K/J_H \approx 0.2$ (denoted as g'_Q in Fig. S21(A)). We expect that g'_Q and g'_M will merge once the coexisting phase is fully suppressed. The temperature dependence of entropy is shown in Fig. S21(B). Extrapolating the entropy in the quantum critical region (decoupled phase) at g'_Q to zero temperature gives a residual entropy $S/N \approx 0.62$ (see the inset of Fig. S21(E)). The finite zero-temperature entropy we find at the QCP of the two-impurity Kondo model is in stark contrast to the vanishing zero-temperature entropy we found in the strange metal phase of the lattice model (see Fig. S11(A)). Within the valence bond phase, both the f-spinons and χ-holons show a gap in their density of states, as is shown in Fig. S21(C) and S21(D). This is qualitatively different from the lattice case, where the RVB strange metal phase is gapless in the presence of PH symmetry. The difference comes from the dispersive f-spinons in the lattice case, which gives rise to a Van-Hove singularity at $\kappa = 0.5$. The specific heat coefficient, $C_V/(NT)$, and the scattering T-matrix ($-N\mathcal{T}''(\omega=0)$) in the quantum critical region (decoupled phase) both show asymptotically power-law behaviors at the critical ratio $T_K/J_H = 0.2$, as shown in Figs. S21(E) and S21(F). However,

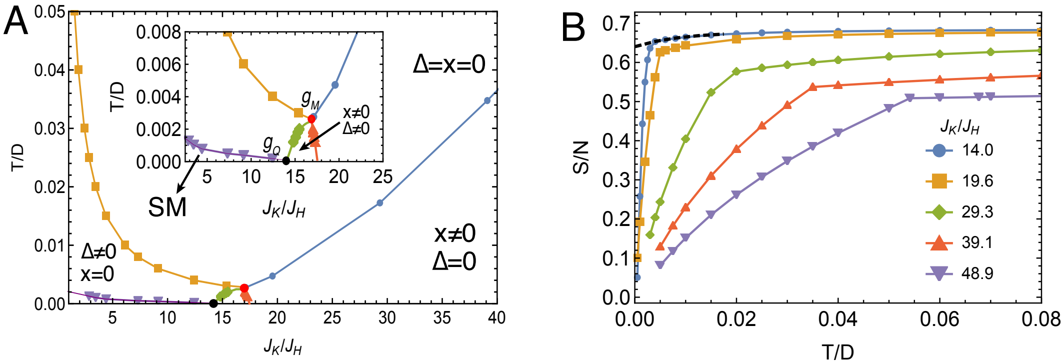

Fig. S22. The Kondo lattice model with $J_K' = 1.145J_K$. (A) The phase diagram of the Kondo lattice model under fine tuning of J_K and J_H. The RVB phase ($x = 0$, $\Delta \neq 0$) is found by fixing $J_K = 0.35$ and decreasing J_H, while the Kondo phase ($x \neq 0$, $\Delta = 0$) is found by fixing $J_H = 0.02$ and increasing J_K. Their phase boundaries merge at $J_K = 0.35$ and $J_H = 0.02$, corresponding to $J_K/J_H \approx 17$ (denoted as g_M). The scattering T-matrix shows a power-law strange metal behavior at low temperature in the RVB phase (below the blue line with triangle markers). The inset shows an enlargement of the coexisting region. (B) The temperature dependence of entropy. The dashed curve is an extrapolation of the entropy of decoupled phase to zero temperature.

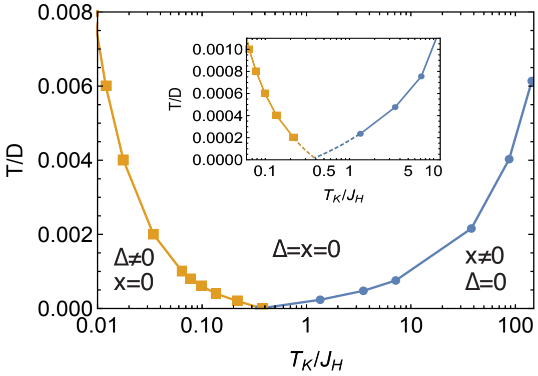

Fig. S23. Phase diagram of two impurity Kondo model with $J_K' = 1.03J_K$. Inset shows an enlargement of the critical region.

these NFL behaviors found in our two-impurity Kondo model are distinct from that of the strange metal phase we found in the lattice model. Based on the differences in thermodynamic and transport properties at $T \to 0$ and finite temperatures between the QCP of our two-impurity model and the strange metal phase of our lattice model, we conclude that the strange metal fixed points and the QCP of the two-impurity model are distinct fixed points and belong to different universality classes. We notice that the thermodynamic and transport properties at the QCP of our two-site model are somewhat different from the behavior of the Jones-Varma QCP. We suspect that this is due to the negligence of fluctuation of the RVB field Δ_{ij} in our self-consistent calculation. To fully recover the critical properties of the Jones-Varma QCP of the two-impurity Kondo model, we need to include the fluctuation of the RVB spin-singlet field, Δ_{ij}.

Via a similar fine-tuning approach of J_K and J_H, we are able to significantly suppress the coexisting phase for our lattice model. The corresponding phase diagram with $J_K' = 1.145J_K$ is shown in Fig. S22(A). The boundary of the RVB phase ($x = 0$, $\Delta \neq 0$) is identified by fixing $J_K = 0.35$ and decreasing J_H, while the boundary of the Kondo phase ($x \neq 0$, $\Delta = 0$) is found by fixing $J_H = 0.02$ and increasing J_K. The two boundaries merge at $J_K = 0.35$ and $J_H = 0.02$ [the point denoted as g_M in Fig. S22(A)]. The QCP between the RVB and coexisting phase locates at $J_K/J_H \approx 14$ (denoted as g_Q in Fig. S22(A)). We find a negligible coexisting phase as that for two-impurity Kondo model mentioned above. Inside the RVB phase, the T-matrix shows a power-law-in-temperature strange metal behavior below the crossover temperature (the purple line with triangle markers in Fig. S22(A)), similar to power-law behavior of T-matrix shown in the main text but with a different power-law exponent. Fig. S22(B) shows the temperature dependence of entropy. Extrapolating the entropy of the decoupled phase at g_Q to zero temperature gives a residual entropy $S/N \approx 0.64$.

Furthermore, following this fine-tuning approach to the QCP, with J_K'/J_K being further reduced to close to the channel-symmetric limit ($J_K'/J_K = 1^+$), a full suppression of the coexisting phase is achieved. This gives rise to a single QCP separating the valence bond phase and the Kondo singlet phase. Fig. S23 shows the phase diagram of two-impurity Kondo model with a choice of $J_K'/J_K = 1.03$ where no coexisting phase can be found within our numerical accuracy. We expect that similar situation happens to the Kondo lattice when J_K'/J_K is tuned to 1^+, where the superconducting phase becomes negligible.

References

1. S Kirchner, et al., Colloquium: Heavy-electron quantum criticality and single-particle spectroscopy. *Rev. Mod. Phys.* **92**, 011002 (2020).
2. Y Komijani, P Coleman, Model for a ferromagnetic quantum critical point in a 1D Kondo lattice. *Phys. Rev. Lett.* **120**, 157206 (2018).
3. Y Komijani, P Coleman, Emergent critical charge fluctuations at the Kondo breakdown of heavy fermions. *Phys. Rev. Lett.* **122**, 217001 (2019).
4. MA Cazalilla, Easy-axis ferromagnetic chain on a metallic surface. *J. Physics: Condens. Matter* **25**, 094008 (2013).
5. B Shen, et al., Strange-metal behaviour in a pure ferromagnetic Kondo lattice. *Nature* **579**, 51–55 (2020).
6. Q Si, JX Zhu, DR Grempel, Magnetic quantum phase transitions in Kondo lattices. *J. Physics: Condens. Matter* **17**, R1025–R1040 (2005).
7. P Coleman, *Introduction to Many-Body Physics.* (Cambridge University Press), (2015).
8. O Parcollet, A Georges, G Kotliar, A Sengupta, Overscreened multichannel SU(N) Kondo model: Large-N solution and conformal field theory. *Phys. Rev. B* **58**, 3794 (1998).
9. AC Hewson, *The Kondo problem to heavy fermions.* (Cambridge university press) Vol. 2, (1997).
10. M Hermele, et al., Stability of $U(1)$ spin liquids in two dimensions. *Phys. Rev. B* **70**, 214437 (2004).
11. SS Lee, Stability of the u(1) spin liquid with a spinon fermi surface in $2+1$ dimensions. *Phys. Rev. B* **78**, 085129 (2008).
12. A Polyakov, Compact gauge fields and the infrared catastrophe. *Phys. Lett. B* **59**, 82 – 84 (1975).
13. A Polyakov, Quark confinement and topology of gauge theories. *Nucl. Phys. B* **120**, 429 – 458 (1977).
14. N Read, S Sachdev, Valence-bond and spin-peierls ground states of low-dimensional quantum antiferromagnets. *Phys. Rev. Lett.* **62**, 1694–1697 (1989).
15. P Coleman, I Paul, J Rech, Sum rules and Ward identities in the Kondo lattice. *Phys. Rev. B* **72**, 094430 (2005).
16. P Coleman, N Andrei, Kondo-stabilised spin liquids and heavy fermion superconductivity. *J. Phys. Condens. Matter* **1**, 4057–4080 (1989).
17. YY Chang, et al., Strange superconductivity near an antiferromagnetic heavy fermion quantum critical point. *Phys. Rev. B* **99**, 094513 (2019).
18. S Jang, et al., Evolution of the kondo lattice electronic structure above the transport coherence temperature. *Proc. Natl. Acad. Sci.* **117**, 23467–23476 (2020).
19. T Senthil, S Sachdev, M Vojta, Fractionalized Fermi liquids. *Phys. Rev. Lett.* **90**, 216403 (2003).
20. J Ye, S Sachdev, Superconducting, metallic, and insulating phases in a model of CuO_2 layers. *Phys. Rev. B* **44**, 10173–10189 (1991).
21. AJ Millis, AJ Schofield, GG Lonzarich, SA Grigera, Metamagnetic quantum criticality in metals. *Phys. Rev. Lett.* **88**, 217204 (2002).
22. GM Schmiedeshoff, et al., Multiple regions of quantum criticality in YbAgGe. *Phys. Rev. B* **83**, 180408(R) (2011).
23. E Lebanon, P Coleman, Fermi liquid identities for the infinite-U multichannel Anderson model. *Phys. Rev. B* **76**, 085117 (2007).
24. L Balents, Spin liquids in frustrated magnets. *Nature* **464**, 199–208 (2010).
25. BA Jones, CM Varma, JW Wilkins, Low-temperature properties of the two-impurity kondo hamiltonian. *Phys. Rev. Lett.* **61**, 125–128 (1988).
26. BA Jones, CM Varma, Critical point in the solution of the two magnetic impurity problem. *Phys. Rev. B* **40**, 324–329 (1989).
27. G Zaránd, CH Chung, P Simon, M Vojta, Quantum criticality in a double-quantum-dot system. *Phys. Rev. Lett.* **97**, 166802 (2006).
